# Characterization of the dual functions of *Leishmania* CK1.2 in both the parasite and the macrophage

**DOI:** 10.1371/journal.pone.0356818

**Published:** 2026-10-05

**Authors:** Daniel Martel, Olivier Leclercq, Florent Dingli, Victor Laigle, Nawal Hajj Sleiman, Damarys Loew, Gerald F. Späth, Najma Rachidi

**Affiliations:** 1 Institut Pasteur, Université Paris Cité and INSERM U1347, Groupe de signalisation et interactions hôte-parasite, Paris, France; 2 Institut Pasteur, Université Paris Cité and INSERM U1347, Unité de Parasitologie moléculaire et Signalisation, Paris, France; 3 Laboratoire de Spectrométrie de Masse Protéomique (LSMP), Centre de Recherche, Institut Curie, PSL Research University, Paris, France; Indian Institute of Technology (IIT BHU) - Varanasi, INDIA

## Abstract

*Leishmania* CK1.2 (L-CK1.2) is a serine/threonine protein kinase essential for the survival of the protozoan parasite *Leishmania*, the causative agent of leishmaniasis. This study investigates the dynamic localization pattern of L-CK1.2 and the broad spectrum of its interacting partners within the parasite. Using proteomic analysis and confocal microscopy, we identified 230 L-CK1.2-associated proteins across the major parasite life stages. This analysis revealed the ubiquitous presence of L-CK1.2 in various cellular structures, including the cytoskeleton, basal body, and flagellum. Using an *in vitro* system, sixty-four host proteins, involved in critical host biological processes such as immune response, apoptosis, and purine biosynthesis, were identified as potential interacting partners of L-CK1.2. These processes are known to be regulated by *Leishmania* during infection. The study highlights the potential dual function of L-CK1.2, in the parasite (*cis*) and within the host cell (*trans*), positioning this kinase as a key player in host-pathogen interactions. This work provides a comprehensive map of L-CK1.2 interactions and suggests its potential importance in regulating intracellular *Leishmania* survival, providing potential targets for leishmaniasis therapy. Furthermore, given the evolutionary conservation of CK1.2 across other parasitic organisms, our findings may have broader implications for understanding and managing parasitic infections.

## Introduction

CK1 family members are serine/threonine protein kinases ubiquitously expressed in eukaryotic organisms [1]. They are involved in a wide range of important cellular processes, such as membrane trafficking or vesicular transport from yeast to humans [1]. Due to its broad spectrum of action, CK1 activity and expression are tightly regulated by several mechanisms, including phosphorylation and subcellular sequestration [1]. Indeed, members of the CK1 family are associated with many subcellular structures and organelles through interactions with specific partners ranging from circadian clock to cytoskeletal elements [2–7], reflecting the strong interconnection of CK1 localisation and its functions. Defects in regulation, localisation or the introduction of mutations in the CK1 coding sequence are often associated with important diseases like cancer or neurodegenerative diseases [8–10]. Regulation of CK1 pathways has also been linked to infectious diseases. For instance, inhibiting host CK1 suppresses yellow fever virus replication [11], highlighting the crucial role of CK1 in host-pathogen interactions.

The protozoan parasite *Leishmania* is the causative agent of leishmaniasis, a potentially life-threatening disease with at least three distinct clinical forms, cutaneous, mucocutaneous and the fatal visceral leishmaniasis. *Leishmania* has two developmental stages, an extracellular promastigote stage that proliferates inside the insect vector, and an intracellular amastigote stage that develops and multiplies inside the phagolysosomes of macrophages. The *Leishmania* genome encodes for six CK1 paralogs [12], of which only CK1.2 and CK1.4 are secreted by the parasite and were shown to be essential for parasite survival [13,14]. Several lines of evidence suggest that CK1.2 is released within the host cell as free protein or via extracellular vesicles [15–17]. The adaptation of L-CK1.2 to two distinct cellular environments, driven by the high conservation of its protein sequence in *Leishmania* spp. and strong identity to its human orthologs, suggests that this protein has been shaped by selective pressures acting both on its functions within the host cell [12,18]. Indeed, our previous study identified potential substrates of L-CK1.2 in the macrophage, indicating its ability to regulate multiple host biological processes, such as ‘viral and symbiotic interaction’, ‘actin cytoskeleton organization’ and ‘apoptosis’ [19]. These potentially dual functions, in *cis* in the parasite and in *trans* in the host macrophage [12,18,19], suggest *Leishmania* CK1.2 as a key player in host-pathogen interactions. Despite its importance in parasite biology and infection, the functions of L-CK1.2 remain poorly understood, beyond its known interactions with and phosphorylation of heat shock proteins within the parasite, and its ability to phosphorylate host targets such as IFNAR1/2 (interferon-α/β receptors) [12,13,19–22]. The essential nature of this kinase and its involvement in most cellular functions present major challenges for functional analysis using genetic approaches. To overcome these limitations and gain insights into the cis and trans functions of L-CK1.2, we chose a complementary approach determining and comparing its interactomes in both the parasite and the host cell. Our proteomic analysis unveils a repertoire of 230 interacting partners for L-CK1.2 across both amastigote and promastigote life stages of the parasite, with Gene Ontology enrichment analyses indicating its ubiquitous nature and multifaceted localization within various organelles. Applying confocal microscopy, we confirmed the localization of L-CK1.2 at these key subcellular sites, including the cytoskeleton, basal body, and flagellum. We used an *in vitro* approach to further identify 64 host proteins interacting with L-CK1.2, which again are implicated in a wide range of biological processes such as immune response, apoptosis and purine biosynthetic process. These biological processes are also modulated during *Leishmania* infection, strongly suggesting the involvement of L-CK1.2 in subverting host cell signalling. Our results suggest that certain pathways identified in the parasite as potential targets of L-CK1.2 are similarly found in the host. Collectively, our data provide additional evidence for the essential role of L-CK1.2, as a central component of the trans-signalling network between *Leishmania* and the macrophage. This dual biological function opens exciting avenues for future research on the co-evolution of parasite and macrophage biology, and the discovery of host-directed therapeutic interventions.

## Materials and methods

### Ethics statement

Work on animals was performed in compliance with French and European regulations on the care and protection of laboratory animals (EC Directive 2010/63; French Law 2013−118, February 6, 2013). All animal experiments were approved by the Ethics Committee and the Animal Welfare Body of the Institut Pasteur, as well as by the Ministère de l'Enseignement Supérieur, de la Recherche et de l'Innovation (Projects No. 19683 and 240013).

### *Leishmania* cell lines

All the parasite cell lines used in this study were derived from *L. donovani* axenic 1S2D (MHOM/SD/62/1S-CL2D) clone LdBob, obtained from Steve Beverley, Washington University School of Medicine, St. Louis, MO. Promastigotes were cultured and differentiated into axenic amastigotes as described previously [23]. Parasites cell lines were grown in media with 30 µg/mL hygromycin B (ThermoFisher Scientific Cat# 10687010) to maintain the pLEXSY-CK1.2-V5 or the empty pLEXSY plasmids. The transgenic *L. donovani* cell lines containing either the pLEXSY or pLEXSY-CK1.2-V5-HIS_6_ (pLEXSY-CK1.2-V5) vectors, corresponding to the mock or expressing *Leishmania major* CK1.2-V5 tagged with V5 and HIS_6_, respectively, were described previously [18]. L-CK1.2-V5 in this strain was shown to be active, functional, able to rescue a decrease in the activity of endogenous CK1.2-V5 and its ectopic expression did not alter the parasite phenotypes.

### Immunofluorescence

To confirm the localisation of CK1.2-V5 inferred from the interactome dataset, we used the *Leishmania donovani* mock and CK1.2-V5 parasites (see Mat & Met and [18]). Although, CK1.2-V5 is expressed ectopically, its abundance is half of the endogenous CK1.2-V5, precluding the risk of localisation artefact due to overexpression. Logarithmic phase promastigotes or axenic amastigotes (48h after shift at 37˚C and pH5.5) were resuspended at 2x10^6^ parasites per mL in Dulbecco’s Phosphate Buffer Saline (DPBS) (Gibco) from at least three biological replicates, and 500 µL were added to poly-L-lysine-coated coverslips placed in a 24-well plate. Plates were centrifuged 10 min at 1200 g at room temperature to settle parasites onto the coverslips. For fixation alone, cells were washed three times with DPBS and fixed in 4% paraformaldehyde (PFA) in DPBS for 15 min at room temperature. For cytoskeleton preparation, the protocol was adapted from [24]. Briefly, cells were washed three times with DPBS, treated with 0.125% Nonidet 40 (Fluka BioChemika Cat# 74385) in PIPES buffer (100 mM piperazine-N,N-bis(2-ethanesulfonic acid) (PIPES) pH6.8, 1 mM MgCl_2_) for 2 min at room temperature and washed twice for 5 min in PIPES buffer. Cells were fixed in 4% PFA in DPBS for 15 min at room temperature. After PFA fixation, cells were washed three times in DPBS, neutralised 10 min with NH_4_Cl (50 mM in DPBS), and washed again three times in DPBS. For the immuno-labelling of PFA-fixed cells or cytoskeleton preparations, the samples were blocked with 10% filtered heat-inactivated fetal calf serum (FCS) containing 0.5 mg.mL^-1^ saponin in DPBS for 30 min at room temperature and then washed for 5 min in DPBS. The cells were then incubated with primary antibodies diluted in DPBS with 0.5% Bovine Serum Albumin (BSA) and 0.5 mg.mL^-1^ saponin for 1h at room temperature. Three washes of 10 min were performed and the secondary antibody diluted in DPBS with 0.5% BSA and 0.5 mg.mL^-1^ saponin was added. After one hour incubation at room temperature in the dark, cells were washed twice for 10 min in DPBS with 0.5% BSA and 0.5 mg.mL^-1^ saponin, and then twice in DPBS. Parasites were incubated with 5 µg.mL^-1^ Hoechst 33342 in DPBS for 8 min in the dark, washed twice with DPBS, one time with distilled water, air-dried, then mounted with slides using SlowFade Gold Antifade Mountant (ThermoFisher Scientific Cat# S36937). For methanol fixation, logarithmic phase promastigotes were washed twice in DPBS and resuspended at 2x10^7^ parasites per mL. 10^6^ parasites were spread onto poly-L-lysine coated slides and allowed to settle for 30 min in a humid chamber. Parasites were then fixed in methanol at −20°C for 3 minutes and rehydrated for 10 min in DPBS at room temperature. For immuno-labelling of methanol-fixed parasites, samples were blocked with 10% filtered heat-inactivated FCS in DPBS for 15 min at room temperature and washed for 5 min in DPBS. Then the cells were treated similarly as those fixed by PFA. The antibodies used were: mouse IgG2a anti-V5 tag monoclonal antibody (Thermo Fisher Scientific Cat# R960-25, RRID:AB_2556564) diluted at 1/200 (in PFA and methanol fixed parasites) or at 1/300 (in cytoskeleton preparations); rabbit anti-V5 tag polyclonal antibody (Abcam Cat# ab9116, RRID:AB_307024) diluted at 1/400; rabbit anti-LdCentrin polyclonal antibody (kind gift from Hira L. Nakhasi) diluted at 1/2000 [25]; mouse IgG1 anti-IFT172 monoclonal antibody diluted at 1/200 [26]; mouse anti-PFR2 L8C4 clone antibody diluted at 1/10 [27]; mouse L1C6 anti-TbNucleolus monoclonal antibody diluted at 1/100 (kind gift from Keith Gull [28]); mouse IgG1 anti-α-tubulin monoclonal DM1A antibody (Sigma-Aldrich Cat# T9026, RRID:AB_477593) diluted at 1/400. IgG subclass-specific secondary antibodies coupled to different fluorochromes were used for double labelling: anti-mouse IgG (H + L) coupled to AlexaFluor488 (1/200 (PFA- or methanol-fixed parasites)) or 1/300 (cytoskeleton preparations), Thermo Fisher Scientific Cat# A-21202, RRID:AB_141607)); anti-mouse IgG2a coupled to Cy3 (1/600; Jackson ImmunoResearch Labs Cat# 115-165-206, RRID:AB_2338695); anti-mouse IgG1 coupled to AlexaFluor647 (1/600; Thermo Fisher Scientific Cat# A-21240, RRID:AB_2535809); anti-rabbit IgG (H + L) coupled to AlexaFluor488 (1/400; Thermo Fisher Scientific Cat# A-21206, RRID:AB_2535792); anti-mouse IgG (H + L) coupled to AlexaFluor594 (1/200; Thermo Fisher Scientific Cat# A-21203, RRID:AB_2535789); anti-mouse IgG2a coupled to AlexaFluor488 (1/300; Thermo Fisher Scientific Cat# A-21131, RRID:AB_2535771); anti-mouse IgG1 coupled to AlexaFluor594 (1/300; Thermo Fisher Scientific Cat# A-21125, RRID:AB_2535767) and anti-rabbit IgG (H + L) coupled to AlexaFluor594 (1/400; Thermo Fisher Scientific Cat# A-21207, RRID:AB_141637). All IFA were at least performed with three biological replicates.

### Confocal microscopy

Images were visualised using a Leica SP5 HyD resonant scanner Matrix screener inverted microscope equipped with a HCX PL APO CS 63x, 1.4 NA oil objective (Leica, Wetzlar, Germany). Triple or quadruple immunofluorescence was imaged with Leica Application Suite AF software (LAS AF; Leica Application Suite X, RRID:SCR_013673) after excitation of the Hoechst 33342 dye with a diode at a wavelength of 405 nm (452/75 Emission Filter), excitation of the AlexaFluor488 with an argon laser at a wavelength of 488 nm (525/50 Emission Filter), excitation of AlexaFluor594 with a diode DPSS at a wavelength of 561 nm (634/77 Emission Filter), excitation of Cy3 with a diode DPSS at a wavelength of 561 nm (595/49 Emission Filter), and excitation of AlexaFluor647 with a helium-neon laser at a wavelength of 633 nm (706/107 Emission Filter). Images were scanned sequentially to minimise cross excitation between channels and each line was scanned twice and averaged to increase the signal-to-noise ratio. The pinhole aperture was set to 1 airy. Images were acquired with 8x zoom at a resolution of 1024 × 1024. Z-stacks were acquired at 0.082 µm intervals, deconvolved and rendered using either Fiji (RRID:SCR_002285) or Icy (RRID:SCR_010587) software [29] (http://icy.bioimageanalysis.org/).

### Deconvolution of z-stacks and chromatic aberration correction

All confocal images were processed and analysed by using the Huygens Professional software version 19.04 (SVI). Deconvolution of confocal z-stacks was optimised using the following settings: automatic estimation of the average background with the mode “Lowest” and area radius = 0.7, deconvolution algorithm CMLE, maximum number of iterations = 40, signal to noise ratio (SNR) = 20, quality change threshold = 0.05, iteration mode = optimised, brick layout = automatic. Theoretical point spread function (PSF) values were estimated for each *z*-stack. All deconvolved images were corrected for chromatic shifts and for rotational differences between different channels using the Chromatic Aberration Corrector (CAC) from Huygens Professional software (SVI). To calibrate the image corrections, multifluorescent 0.2 µm TetraSpeck microspheres (ThermoFisher Scientific Cat#T7280) mounted on SlowFade Gold Antifade mountant (ThermoFisher Scientific Cat# S36937) were imaged with identical acquisition parameters. Images were deconvolved similarly and were used to perform the chromatic aberration estimations with the cross-correlation method in CAC software. Corrections were saved as templates and applied for correction of the similarly acquired and deconvolved images in CAC.

### Co-localisation analysis of confocal images

Co-localisation analysis was performed with the Co-localisation Analyzer plug-in of the Huygens Professional software (SVI, Huygens Software, RRID:SCR_014237, v19.04). Processed cross-section images (deconvolved and corrected for chromatic aberrations) of the parasites were opened with this plug-in and Pearson correlation coefficients (PCC) were calculated for each parasite to quantify co-localisation. A PCC above 0.5 indicates co-localisation. The PSFs were automatically calculated by Huygens, based on the microscope and acquisition parameters. The SNR of 20 was set manually. Specific areas of the parasite were cropped from the whole image (basal body area, flagella pocket area, mitotic spindle and reduced mitotic spindle areas, flagellar pocket neck area and flagellar tip area) with the “crop” tool of the Huygens Professional software. Criteria to crop specific structures from the images were: (1) basal bodies – a region containing one or two dots representing the basal bodies in the channel CEN and excluding most of the flagellar pocket and flagellar pocket neck; (2) flagellar pocket area – a region stained with CK1.2-V5 and CEN but excluding the basal bodies dots and the flagellum; (3) mitotic spindle – the entire Hoechst-unstained nucleus region in mitotic cells, containing the mitotic spindle in the red channel; (4) reduced mitotic spindle areas – only the regions containing the tubulin staining in the mitotic nuclei; (5) flagellar pocket neck – a region at the junction of the flagellum and the parasite body, excluding most of the flagellar pocket and basal bodies CK1.2-V5 staining; and [6] flagellar tip – region at the tip of the flagellum, containing only a fraction of the rest of the flagellum. Rationale: since CK1.2-V5 is present in Hoechst-unstained regions where the tubulin staining is not visible, co-localisation of the entire Hoechst-unstained region is biased by a strong staining with CK1.2-V5. We decided to crop further to analyse the colocalisation of CK1.2-V5 and the tubulin in the mitotic spindle stained by anti-tubulin antibody. Pearson coefficients were calculated for these images. Pearson coefficients of the co-localisation in the basal body and flagellar pocket areas of (i) CK1.2-V5 with Centrin (CEN), IFT172, and DNA (Hoechst 33342, H); or (i) CEN with IFT172, from 14 images, from 2 biological replicates, were plotted in scattered dot plots with the mean and standard deviation using GraphPad Prism 8.1.1 (GraphPad Software, GraphPad Prism, RRID:SCR_002798). Pearson coefficients of the co-localisation of CK1.2-V5 with tubulin in the mitotic spindle and reduced mitotic spindle areas from seven images, from 2 biological replicates, were plotted similarly. The statistical significance of the Pearson Correlation Coefficient (PCC) for paired images of L-CK1.2 with Centrin, IFT172, or tubulin (test) and L-CK1.2 with DNA (negative control) was quantified using the Wilcoxon matched-pairs signed rank test (GraphPad Prism). A p-value of < 0.05 was considered significant. P-value: *** (0.001); ** (< 0.01) and * (< 0.05).

### Epifluorescence microscopy and automated parasite detection

Images were visualised using a Zeiss upright widefield microscope equipped with Apotome2 grids and a Pln-Apo 63x, 1.4 NA oil objective (Zeiss). Light source used was a Mercury Lamp HXP 120, and following filters were used: DAPI (Excitation G365; dichroic FT 395; emission BP 420–470), FITC-A488-GFP (Excitation BP 455–495; dichroic FT 500; emission BP 505–555) and A594-TexasRed-mCherry-HcRed-mRFP (Excitation BP 542–582; dichroic FT 593; emission BP 604–644). Images were captured on an Axiocam MRm camera using ZEN Blue software. For comparison of different cell lines, identical parameters of acquisition were applied on all samples.

### Protein extraction, SDS-PAGE and Western blot analysis

Logarithmic phase promastigotes were washed in DPBS and protein extraction was performed as described previously [23]. Ten micrograms of total protein were separated by SDS-PAGE, and transferred onto polyvinylidene difluoride (PVDF) membranes (Pierce). Membranes were blocked with 5% BSA in DPBS supplemented with 0.25% Tween20 (PBST) and incubated over night at 4°C with primary antibody mouse IgG2a anti-V5 tag monoclonal antibody (1/1000; Thermo Fisher Scientific Cat# R960-25, RRID:AB_2556564) or rabbit anti-L-CK1.2 antibody (SY3535) in 2,5% BSA in PBST. Membranes were then washed in PBST and incubated with secondary antibody anti-mouse IgG (H + L) coupled to horseradish peroxidase (1/20000; ThermoFisher Scientific Cat# 32230, RRID:AB_1965958). Proteins were revealed by SuperSignal™ West Pico Chemiluminescent Substrate (ThermoFisher Scientific Cat# 34580) using the PXi image analysis system (Syngene) at various exposure times. Membranes were then stained with Bio-Safe Coomassie (Bio-Rad Cat #1610786) to serve as loading controls.

### Recombinant expression, purification of CK1.2-V5-His_6_

*Escherichia coli* Rosetta (DE3) pLysS Competent Cells (Merck Cat# 70956) containing pBAD-thio-topo-L-CK1.2-V5-His_6_ and pBADthio-V5-His6 (control thioredoxin) were grown at 37°C and induced with arabinose (0,02% final) for 4h at room temperature [18]. Cells were harvested by centrifugation at 10,000 g for 10 min at 4°C and the recombinant proteins were purified as described previously [18,30]. The eluates were supplemented with 15% glycerol and stored at −80°C.

For the SEC, full-length *Leishmania* CK1.2 was cloned into the pET23 expression vector (Novagen) by restriction-free cloning and sequence-verified by Sanger sequencing. *E. coli* BL21 cells (DE3, New England Biolabs) containing pET23-LCK1.2 was grown at 37°C and induced with 0.5 mM IPTG for overnight incubation at 16°C. Cells were harvested by centrifugation and lysed in PBS supplemented with EDTA-free protease inhibitors and Benzonase nuclease. Soluble His_6_-tagged L-CK1.2 was purified by immobilized metal affinity chromatography using cobalt agarose resin, washed with PBS containing 10 mM imidazole, and eluted with increasing imidazole concentrations (20–500 mM). Protein purity was assessed by SDS-PAGE followed by Coomassie staining, and identity confirmed by Western blot using anti-CK1.2 antibody (SY3535). Purified fractions containing L-CK1.2 were pooled and concentrated.

### Production of macrophage extracts

Bone marrow derived macrophages (BMDM) were obtained from BALB/c ByJRj mice [31]. Macrophages were lysed with RIPA buffer (150 mM NaCl, 1% Triton X-100, 20 mM Tris HCl [pH 7.4], 1% NP-40, 1 mM EDTA) supplemented with complete protease inhibitor cocktail (Roche Applied Science, IN) and with 1 mM sodium orthovanadate and 1 mM PMSF. Macrophage lysates were then vortexed and, after sonication, clarified by centrifugation.

### Immuno-precipitation

#### *Leishmania* IP.

*L. donovani* logarithmic phase promastigotes or axenic amastigotes (48h post differentiation) containing either pLEXSY (mock) or pLEXSY-CK1.2-V5-His6 (expressing CK1.2-V5-His6) were collected and total protein were extracted, as described previously [23]. Six (promastigotes) or twelve milligrams (axenic amastigotes) of total proteins were used at 2 mg/mL concentration in RIPA lysis buffer for the immuno-precipitation. The lysates were pre-cleared for 30 min with 1.5 mg of fresh magnetic beads coupled to protein G (ThermoFisher Scientific Cat# 10003D), washed with DPBS-tween buffer (DPBS with 0.02% Tween-20, adjusted at pH 7.4) and mixed with cross-linked anti-V5-coupled magnetic beads according to the manufacturer protocol. After incubation with pre-cleared lysates, beads were washed then the bound proteins were eluted with the glycine elution buffer for the PRO condition and with glycine elution buffer followed by the NuPAGE loading buffer (invitrogen) and heating at 95°C for Ax AMA condition. Elutions were kept at −80°C before mass spectrometry analysis. The experiment was performed with three biological replicates.

#### Host IP.

Purified lysates from bacteria expressing rL-CK1.2-V5, the thioredoxin control or the empty vector were incubated overnight with 142 μg BMDM protein lysates. After preclearing, the samples were incubated with magnetic beads coupled to protein G and cross-linked to anti-V5 antibody. Following washing, the immuno-precipitated proteins were eluted first with glycine elution buffer (elution 1 and 2) then with one elution of NuPAGE loading buffer (elution 3). Elutions 1 and 3 were used for MS analysis and proteins from elution 2 were separated on SDS-PAGE and stained with SYPRO Ruby to assess the IP (Figure 6A). The experiment was performed in three biological replicates.

### Size exclusion chromatography (SEC)

Parasites containing pLEXSY-V5-CK1.2-V5 were harvested from logarithmic-phase cultures by centrifugation at 1,600 × g for 10 min and washed three times in D-PBS without CaCl_2_ and MgCl_2_ (Gibco). Cells were resuspended either in 1X RIPA lysis buffer (Cell Signaling Technology) containing 1 mM Phenylmethylsulfonyl fluoride, 50 U/ml Benzonase nuclease, Purity > 90% (VWR) and protease inhibitors (cOmplete Mini, EDTA-free Protease Inhibitor Cocktail Tablets; Roche). Cells suspensions were incubated on ice for 45 min, disrupted by sonication for 5 min (20s pulse, 20s off) in ice and clarified by centrifugation at 12,000 g for 10 min at 4°C. Total protein extracts were quantified using RC DC Protein Assay Kit II (Bio-Rad) then used immediately. SEC fractionations were performed at 4°C on Superose 6 increase 10/300 GL column (Cytiva) using a ÄKTApurifier FPLC system (GE Healthcare) (P-900 pumps; INV-907 valve; M-925 mixing valves; FR-902 flow Restrictor; FRC-950 fraction collector; UV-900 detector; CU-950 controller) piloted by UNICORN 5.10 software (GE Healthcare). The column was equilibrated with 2 column volumes (CV) of elution buffer (50 mM sodium phosphate; 150 mM NaCl; pH 7.2) at a flow rate of 0.35 ml/min. Then 6.9 milligrams of total protein extract were load in 500 µl sample loop, injected onto the column, and eluted with 1 CV of elution buffer at a flow rate of 0.35 ml/min. Eluted proteins were collected in 96 fractions of 200 µl. Fifteen microliters of each fraction were separated on a Novex NuPAGE 4–12% bis-Tris gel (ThermoFischer scientific) followed by Western blotting. Blots were probed with an anti-V5 antibody and an anti-mouse HRP-conjugated antibody. Signals were revealed using SuperSignal West Pico kit (Pierce), monitored with ChemiDoc imager (Bio-Rad) and quantified using Image Lab Software (Bio-Rad). The SEC with parasite lysates were performed in three replicates and that with recombinant L-CK1.2-V5 were performed in two replicates. The Gel Filtration Calibration Kit HMW used for the SEC was purchased from Cytiva.

### Mass spectrometry analysis

MS analysis of the samples was done at the “Laboratoire de Spectrométrie de Masse Protéomique (LSMP)” platform at Institut Curie in Paris.

#### *Leishmania* IP.

Proteins were run on SDS–PAGE gels (Invitrogen) for a short time just as a clean-up step and stained with colloidal blue staining (LabSafe GEL Blue^TM^ G Biosciences). Gel slices were excised, and proteins were reduced with 10 mM DTT prior to alkylation with 55 mM iodoacetamide. In-gel digestion was performed using trypsin/Lys-C (Promega) overnight in 25 mM NH4HCO3 at 30 °C. Peptides were then extracted using 60/35/5 MeCN/H2O/HCOOH and vacuum concentrated to dryness. Samples were separated by chromatography using an RSLCnano system (Ultimate 3000, Thermo Scientific) coupled to an Orbitrap Fusion mass spectrometer (Q-OT-qIT, Thermo Fisher Scientific). We acquired Survey MS scans in the Orbitrap with the resolution set to a value of 120,000 and a 4 × 10^5^ ion count target. Each scan was recalibrated in real time by co-injecting an internal standard from ambient air into the C-trap. Tandem MS was performed by isolation at 1.6 Th with the quadrupole, HCD fragmentation with normalized collision energy of 35, and rapid scan MS analysis in the ion trap. The MS2 ion count target was set to 10^4^ and the max injection time was 100 ms. Only those precursors with charge state 2–7 were sampled for MS2. The dynamic exclusion duration was set to 60s with a 10 ppm tolerance around the selected precursor and its isotopes. The instrument was run in top speed mode with 3 sec cycles.

For identification, the data were searched against the Ld1S2D database (https://www.ncbi.nlm.nih.gov/bioproject/PRJNA396645, GCA_002243465.1) using Sequest HT through Proteome Discoverer (version 2.4). Enzyme specificity was set to trypsin and a maximum of two missed cleavages was allowed. Oxidized methionine and N-terminal acetylation were set as variable modifications and carbamidomethyl cysteine as fixed modification. The mass tolerances in MS and MS/MS were set to 10 ppm and 0.6 Da, respectively. The resulting files were further processed using myProMS v3.9.3 (https://github.com/bioinfo-pf-curie/myproms [32],). False-discovery rate (FDR) was calculated using Percolator [33] and was set to 1% at the peptide level for the whole study. Label-free quantification was performed using peptide extracted ion chromatograms (XICs), re-extracted within conditions, and computed with MassChroQ v.2.2.21 [34]. For protein quantification, XICs from proteotypic peptides shared between compared conditions (TopN matching) with missed cleavages were used. Median and scale normalization at peptide level was applied on the total signal to correct the XICs for each biological replicate (N = 3 in both conditions). To estimate the significance of the change in protein abundance, a linear model (adjusted on peptides and biological replicates) was performed, and p-values were adjusted using the Benjamini–Hochberg FDR procedure. Proteins with at least two distinct peptides in two replicates of a same state, 2-fold enrichment and an adjusted p-value ≤ 0.05 were considered significantly enriched in sample comparisons. Proteins unique to a condition were also considered if they matched the peptides criteria. The mass spectrometry proteomics raw data have been deposited to the ProteomeXchange Consortium via the PRIDE [35] partner repository with the dataset identifier: PXD033809 (https://www.ebi.ac.uk/pride/archive/projects/PXD033809).

#### Host IP.

The elutions 1 and 3 were also run on SDS-PAGE for a short time and only one gel slice containing the entire elution fraction was excised and used for nanoscale liquid chromatography coupled to tandem mass spectrometry (nanoLC-MS/MS) as described in the paragraph *Leishmania IP*. For protein identification, the data were searched against the databases containing SwissProt Mus Musculus (16745 sequences), contaminants and Uniprot *L. donovani* CK1.2 by using Sequest HT from Proteome Discoverer (version 2.4 or 2.1). The mass spectrometry proteomics raw data have been deposited to the ProteomeXchange Consortium via the PRIDE [35] partner repository with the dataset identifier: Project accession: PXD064006 (https://www.ebi.ac.uk/pride/archive/projects/PXD064006).

All proteomic analyses were performed in three biological replicates.

### Bioinformatics

Gene ontology analyses were performed using either PANTHER (http://www.pantherdb.org/) protein class [36] using *L. major* orthologs as input, or TriTrypDB (https://tritrypdb.org/tritrypdb/app) for biological process and cellular component using *L. donovani BPK282A1* as input [37]. STRING database, was used to visualize protein complexes among the L-CKAP and L-CKAPhost (https://string-db.org/) [38]. The localisation of *T. brucei* proteins was determined using TrypTag (http://tryptag.org/) [39]. The localisation of *Leishmania mexicana* proteins was determined using LeishTag (https://www.leishtag.org/). For motif determination, we used SMART (http://smart.embl-heidelberg.de/). The dataset was analysed for protein-protein interactions and visualized using the STRING plugin (string, https://string-db.org/ [40]) of the Cytoscape software package (version 3.8.2, https://cytoscape.org/[41]). Each node represents a substrate, and each edge represents a protein-protein interaction. The text was improved using ChatGPT 3.5 with the prompt, rewrite or improve (version 3.5, https://openai.com/chatgpt).

### Differential gene ontology enrichment analysis

Gene Ontology (GO) term enrichment was first performed independently for each condition using TriTrypDB [37] against the *Leishmania donovani* BPK282A1 reference annotation [42]. To identify GO terms differentially enriched between promastigotes (N1 = 84 input genes) and axenic amastigotes (N2 = 146 input genes), a pairwise comparative analysis was performed in Python 3 using a union strategy (outer join), whereby all GO terms detected in either condition was retained. For GO terms absent from one condition, the number of annotated input genes (k) was set to zero, reflecting the assumption that no gene from that input list was significantly associated with the term. For each GO term retained, a 2 × 2 contingency table was constructed as:


$$\begin{array}{c} \left[\begin{array}{cc} k_{\text{1}} & k_{\text{2}}\\ N_{\text{1}}-k_{\text{1}} & N_{\text{2}}-k_{\text{2}} \end{array}\right] \end{array}$$

where k1 and k2 denote the number of genes from the promastigote condition and the axenic amastigote condition input lists, respectively, annotated to the GO term under consideration (k = 0 when the term was absent from the corresponding enrichment output). N1 and N2 represent the total numbers of genes submitted to the GO analysis for each condition. This formulation normalises enrichment by input list size (k / N), correcting for the bias introduced when N1 ≠ N2, which would otherwise artificially inflate or deflate odds ratios independently of any biological effect. Two-sided Fisher's exact tests were applied to each GO term using the scipy.stats.fisher_exact function [43]. Odds ratios (OR) and 95% confidence intervals (CI) were estimated using the Haldane–Anscombe continuity correction, which consists of adding 0.5 to each cell of the contingency table prior to calculation, thereby stabilising variance estimates when cell counts are zero or very small [44,45]. Multiple testing correction was applied across all tested GO terms using the Benjamini–Hochberg false discovery rate (FDR) procedure [46] as implemented in the multiple tests function of the statsmodels Python package [47]. GO terms with FDR-adjusted p-values below 0.05 were considered significantly differentially enriched between the two conditions. An OR > 1 indicates preferential enrichment in promastigote condition, whereas an OR < 1 indicates preferential enrichment in axenic amastigote condition. All analyses and visualisations were performed in Python 3 using pandas [48], NumPy [49], SciPy [43], statsmodels [47], and Matplotlib [50].

The Python analysis pipeline used in this study was developed with the assistance of Claude (Anthropic, 2026, Claude 4.6 Sonnet; San Francisco, CA: Anthropic PBC. Available at: https://www.anthropic.com/claude), a large language model, for code generation and methodological guidance. All statistical methods, biological interpretations, and final decisions were reviewed and validated by the authors. The pipeline is deposited at https://doi.org/10.6084/m9.figshare.32177910 and should be opened in Collab or is accessible as Collab notebook: https://colab.research.google.com/drive/1cuGs65bMl7cSM5tJ-1tVFJRc8B1ofQ7R?usp=sharing.

### Quantification and statistical analysis

Statistical analyses were performed with GraphPad Prism 8.1.1 (GraphPad Software, GraphPad Prism, RRID:SCR_002798) using unpaired t test (parametric test). Graphs were drawn using the same software. All errors correspond to the 95% confidence interval. Statistically significant differences are indicated with three (p < 0.01), four (p < 0.001) or five asterisks (p < 0.0001). The number of samples analysed for each experiment is indicated in figure legends.

### Resource table

**Table:** 

REAGENT or RESOURCE	SOURCE	IDENTIFIER
Antibodies		
Anti-V5 tag IgG2a mouse monoclonal	Thermo Fisher Scientific	Cat# R960-25, RRID:AB_2556564
Anti-Centrin from *L. donovani* rabbit polyclonal	[25]	N/A
Anti-IFT172 from *T. brucei* IgG1 mouse monoclonal	[26]	N/A
Anti-V5 tag IgG rabbit polyclonal	Abcam	Cat# ab9116, RRID:AB_307024
Anti-PFR2 from *T. brucei* mouse polyclonal (L8C4)	[27]	N/A
Anti-nucleolus IgG from *T. brucei* mouse monoclonal (L1C6)	Keith Gull, University of Oxford, UK	N/A
Anti-alpha-tubulin IgG1 mouse monoclonal DM1A	Sigma-Aldrich	Cat# T9026, RRID:AB_477593
Anti-mouse IgG (H + L) coupled to AlexaFluor488	Thermo Fisher Scientific	Cat# A-21202, RRID:AB_141607
Anti-mouse IgG2a coupled to Cy3	Jackson ImmunoResearch Labs	Cat# 115-165-206, RRID:AB_2338695
Anti-mouse IgG1 coupled to AlexaFluor647	Thermo Fisher Scientific	Cat# A-21240, RRID:AB_2535809
Anti-rabbit IgG (H + L) coupled to AlexaFluor488	Thermo Fisher Scientific	Cat# A-21206, RRID:AB_2535792
Anti-mouse IgG (H + L) coupled to AlexaFluor594	Thermo Fisher Scientific	Cat# A-21203, RRID:AB_2535789
Anti-mouse IgG2a coupled to AlexaFluor488	Thermo Fisher Scientific	Cat# A-21131, RRID:AB_2535771
Anti-mouse IgG1 coupled to AlexaFluor594	Thermo Fisher Scientific	Cat# A-21125, RRID:AB_2535767
Anti-rabbit IgG (H + L) coupled to AlexaFluor594	Thermo Fisher Scientific	Cat# A-21207, RRID:AB_141637
Anti-mouse IgG (H + L) coupled to horseradish peroxidase	Thermo Fisher Scientific	Cat# 32230, RRID:AB_1965958
Bacterial Strains		
*Escherichia coli* Rosetta (DE3) pLysS Competent Cells	Merck	Cat# 70956
Escherichia coli BL21 (DE3)	New England Biolabs	Cat# C2527H
Experimental Models: Organisms/Cell lines		
*Ld*Bob cells with pLEXSY vector (mock)	[18]	N/A
*Ld*Bob cells with pLEXSY-CK1.2-V5-His_6_ vector, expressing *Lma*CK1.2-V5-His_6_	[18]	N/A
Chemicals, Peptides, and Recombinant Proteins		
Nonidet P40 (NP40)	Fluka BioChemika	Cat# 74385
Ammonium Chloride (NH_4_Cl)	VWR Chemicals	Cat# 21235.297
Carbobenzoxy-Leu-Leuleucinal (MG132)	Sigma-Aldrich	Cat# M7449
SlowFade Gold Anti-Fade	ThermoFisher Scientific	Cat# S36937
Recombinant thio-*Lma*CK1.2-V5-His_6_	[18]	N/A
Recombinant MBP	Sigma-Aldrich	Cat# M1891
Recombinant DNA		
Plasmid: pLEXSY-hyg2 (HygR)	Jena Bioscience	Cat# EGE-232
Plasmid: pLEXSY-CK1.2-V5-His_6_ (HygR)	[18]	N/A
Software and Algorithms		
Icy	Icy	RRID:SCR_010587
GraphPad Prism	GraphPad	RRID:SCR_002798
CytExpert Software	Beckman Coulter	RRID:SCR_017217, v2.2.0.97
Fiji	Fiji	RRID:SCR_002285
Huygens Professional	Scientific Volume Imaging (SVI)	RRID:SCR_014237
Leica Application Suite AF (LAS AF)	Leica	RRID:SCR_013673

## Results

### *Leishmania* CK1.2 interacts with numerous proteins in promastigotes and in axenic amastigotes

To assess whether L‑CK1.2 engages in protein‑protein interactions in *Leishmania* promastigotes, we subjected lysates of transgenic *L. donovani* Bob (*Ld*Bob) parasites carrying pLEXSY-CK1.2-V5-His_6_ [18] to size‑exclusion chromatography (SEC) and analysed the distribution of the kinase across the elution profile. As shown in Fig 1Aa (blue circles), L‑CK1.2-V5 was recovered in fractions spanning from 8 mL to 20 mL. The quantification of the Western blots (S1A Fig) is presented in Fig 1A–b (bottom panel). With the exception of a strong enrichment observed in 17–18 mL fraction, attributed to the free monomer based on our calibration (S1Ba and S1Bb Figs, bottom panel), L-CK1.2-V5 was distributed evenly throughout the remaining fractions. These data indicate that cellular L‑CK1.2 also exists within large complexes and not just as a free monomer. We excluded the possibility that free L-CK1.2 monomers were broadly distributed across the column, as recombinant L-CK1.2-V5 eluted strictly in later fractions (17.8 mL; S1B a–c Figs). This profile contrasts sharply with that of L-CK1.2-V5 immunoprecipitated from parasites (S1Bb Fig, upper panel). Additionally, we observed high molecular weight aggregates in the recombinant protein eluting at 7.8 mL (S1B a–c Figs), an artifact inherent to recombinant production. The presence of L-CK1.2-V5 in complexes was further supported by its migration at a high molecular weight (above 170 kDa) on non-denaturing gels (S1C Fig). Collectively, these data support the notion that *Leishmania* CK1.2 participates in multi‑protein complexes, underscoring the functional relevance of protein‑protein interactions for this kinase.

**Fig 1:**
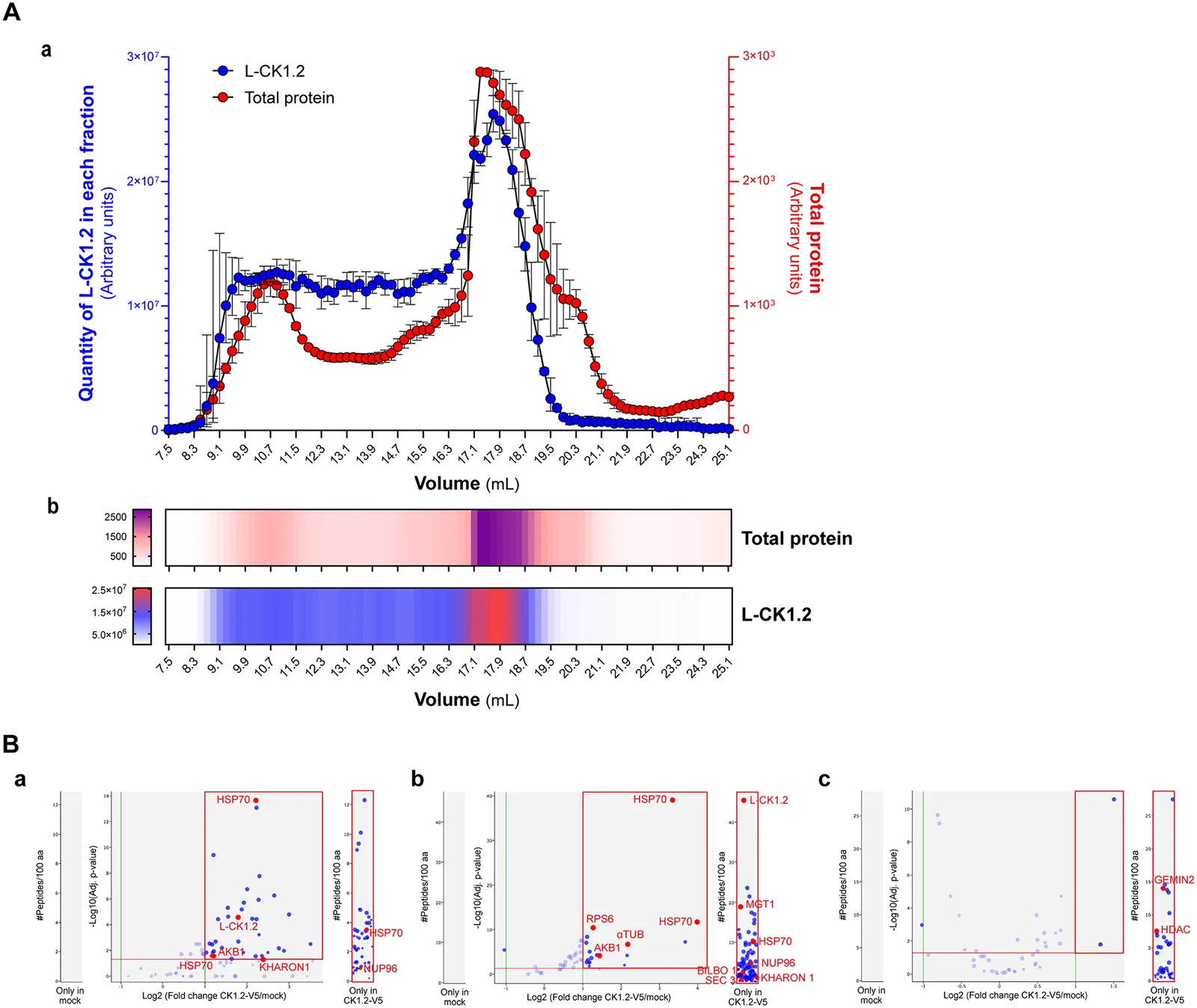
*Leishmania* CK1.2-V5 interacting partners implicated in multiples biological processes. (A) Size-exclusion chromatography (SEC) of L-CK1.2-V5-containing complexes. 3.5 mg of protein, extracted in native conditions from LdBob pLEXSY-CK1.2-V5 were separated by SEC on a superose 6 increase 10/300 GL columns (Total protein, red circles). Fractions of 200 µl were collected and 15 µl of each fraction was separated by SDS-PAGE in denaturing conditions and analyzed by Western blotting using an anti-V5 antibody. The quantification of the three biological replicates using the Biorad chemidoc imaging system (blue circle, bottom panel) and the total protein (red circle, upper panel) is presented in a and b. Each vertical line represents a fraction (from A1 to H12). MW = Molecular Weight. (B) Volcano plots representing the changes in protein abundance of LCKAPs in PRO (a), AxAMA elution E1 (b) and E2 (c) experiments (1 peptide in 1 biological replicate). E1 and E2 are from two consecutive elutions: E1 with glycine elution buffer followed by E2 with NuPAGE loading buffer. Proteins were selected if identified with at least 2 peptides in at least 2 replicates either only in the L-CK1.2-V5 conditions, or with a fold change above 2 and a p-value ≤ 0.05 compared to the mock conditions. Proteins highlighted in red are among those with at least 2 distinct peptides in at least 2 replicates. Mass spectrometry experiments were done from 3 biological replicates.

To identify L-CK1.2-V5 associated proteins (L-CKAP) influencing the regulation, functions or subcellular localisation of this kinase, we used the transgenic *L. donovani* Bob (*Ld*Bob) parasites carrying either the empty pLEXSY vector (referred to as ‘mock’) or pLEXSY-CK1.2-V5-His_6_ (referred to as ‘CK1.2-V5’[18],), previously characterised and functionally validated [18]. Three independent immuno-precipitations (IP) with logarithmic phase promastigotes (PRO) and axenic amastigotes (AxAMA Elution1, E1 & Elution 2, E2) cell lysates were performed using a mouse monoclonal anti-V5 antibody. The eluted proteins were run on SDS-PAGE and analysed by quantitative label-free mass spectrometry. We identified 1081 proteins in promastigote and 516 in amastigote, from which respectively 645 (Fig 1Ba) and 341 (Figs 1Bb and 1Bc) were quantified (for the list of all quantified proteins, see S1 Table). In addition to L-CK1.2-V5 itself, 84 proteins in PRO and 146 in AxAMA (S2 Table) were selected as potential L-CKAPs, based on the following criteria: proteins identified with 2 or more distinct peptides, a fold change ratio CK1.2-V5/mock above 2 and a p-value below 0.05 in at least two replicates (Fig 1Ba-c, red square and S2 Table), as well as those uniquely identified in CK1.2-V5 by at least 2 peptides or more in at least two replicates (Fig 1Ba-c, “only in CK1-V5” and S2 Table). Forty-two L-CKAPs were shared between both parasite stages (S2 Table, green). Half of the proteins identified in PRO and two third of those identified in AxAMA were stage-specific interactors of L-CK1.2-V5 in our conditions. The dissimilarity between the two developmental stages becomes even more apparent when considering the enrichment of Gene Ontology (GO) terms associated with protein class (see S1D Fig and S3 Table, PANTHER: http://www.pantherdb.org/) or biological processes (see Fig 2A and S4 Table, Tritrypdb (https://tritrypdb.org/tritrypdb/app). In promastigotes, L-CKAPs are significantly over-represented in terms like ‘translation’ (Fig 2A, S1D Fig, S4 and S5 Tables, see asterisks), and exclusively associated with terms such as ‘metabolic process’ (Fig 2A, S4 and S5 Tables, see asterisks). In contrast, terms such as ‘establishment of localisation’ and ‘transport’ are exclusively associated with axenic amastigotes (Fig 2A & S4 and S5 Tables, see asterisks).

**Fig 2:**
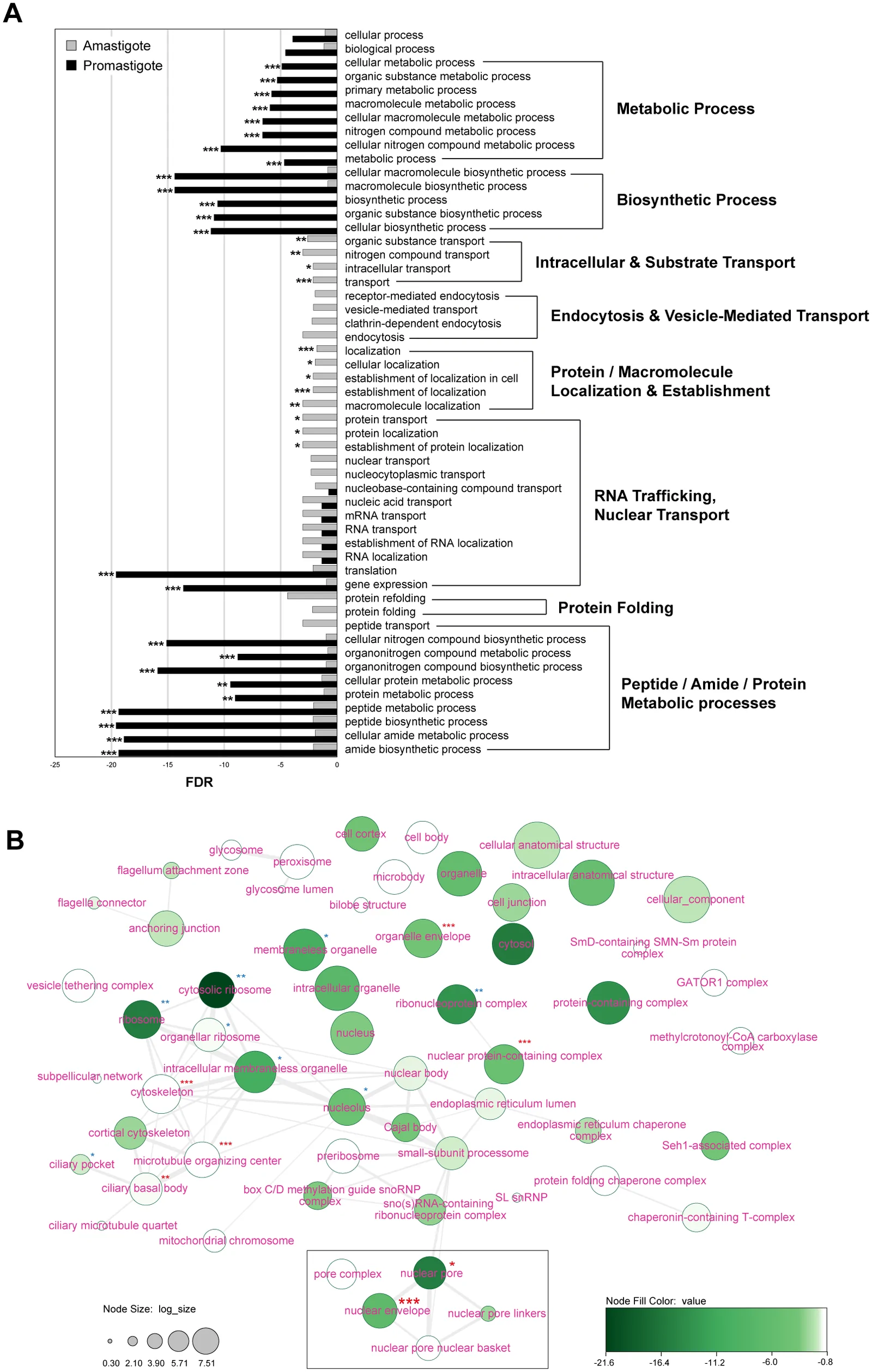
*Leishmania* CK1.2-V5 interacts with numerous proteins in promastigotes and in axenic amastigotes. **(A**) GO term enrichment analysis for the “biological process” ontology was performed for promastigotes and axenic amastigotes using TriTrypDB (https://tritrypdb.org/tritrypdb/app/; see also S4 Table). P-values were adjusted for multiple testing using the Benjamini–Hochberg false discovery rate (FDR) method. Asterisks denote statistical difference in GO term enrichment between promastigotes and axenic amastigotes: ***P < 0.001, **P < 0.01, and *P < 0.05 (see also S5 Table). **(B)** Graphical representation of the GO term enrichment for cellular component from the *Trypanosoma brucei* orthologs of both PRO and AxAMA interacting proteins using cytoscape app, Revigo [98] and tritrypdb GO enrichment (https://tritrypdb.org). The color of the node represents the (Log10(adjusted p-value), between value from −21.6 to −0.8). P-values were adjusted for multiple testing using the Benjamini–Hochberg false discovery rate (FDR) method (significant value are in green, Log10(adjusted p-value) below −1.3). The size of the nodes represents the log size, which is the Log10 (number of annotations for GO Term ID in selected species in the EBI GOA database). Three percent of the strongest GO term pairwise similarities are designated as edges (grey) in the graph [98]. The grey square highlights the nuclear pore complex. The asterisks represent significant difference between the promastigote (blue color, enriched in promastigotes) and axenic amastigote (red color, enriched in Ax. amastigotes) dataset (***P < 0.001, **P < 0.01, and *P < 0.05, see also S7 Table).

To comprehensively determine the potential localisation of these L-CKAPs, we took advantage of the TrypTag (http://tryptag.org/ [39]), a community database providing the localisation of most proteins encoded in the genome of *Trypanosoma brucei*. We hypothesized that L-CKAPs would exhibit similar localisations to those of their syntenic *T. brucei* orthologs. However, this approach is limited by the evolutionary divergence between *T. brucei* and *L. donovani*. Notably, 36 *Leishmania* proteins, identified in this study, lack *T. brucei* orthologs entirely. A prime example of this divergence is the complete absence of the AP2 complex in *T. brucei* [51]. While leveraging *T. brucei* orthologs provides a valuable framework for hypothesizing the subcellular localization of L-CKAPs, we acknowledge the inherent limitations of this cross-species inference. An ortholog localization in *T. brucei* does not guarantee an identical distribution in *Leishmania*. The localizations proposed herein based on orthologs should thus be interpreted as robust predictive models rather than definitive assignments and necessitates direct experimental validation within the *Leishmania* system. Consequently, when possible, we confirmed the localisation with the LeishTag database (https://www.leishtag.org/?pageType=landing). A total of 143 L-CKAPs were assigned subcellular localisation using TrypTag (S2 Table, column: *T. brucei* & localisation in *T. brucei* orthologs), and 20 using LeishTag (S2 Table, column: localisation in *L. mexicana* paralog & *L. mexicana*). The remaining twenty-four proteins lacked localisation data in both databases or had no ortholog in *T. brucei*. Ontology enrichment for Cellular Component of the L-CKAPs was then performed using the *T. brucei* orthologs and Tritrypdb. L-CKAPs are localised in multiples organelles, including the cytosol, the nucleolus, the basal body, the flagellum, the flagellar pocket, or the nuclear pore complex (Fig 2B & S6 Table (blue for PRO, red for AxAMA and green for both)). A closer examination of potential stage-specific complexes revealed a life-stage-specific association between CK1.2-V5 and nuclear pore complex (NPC) subunits (Fig 2B, grey square). This finding is supported by the significant enrichment of the cellular component GO terms ‘nuclear envelope’ (***, Fig 2B and S7 Table) and ‘nuclear pore’ (*, Fig 2B and S7 Table) in axenic amastigotes. Indeed, L-CK1.2-V5 interacts with only four NPC members in promastigotes (S1E Fig (blue squares) and S6 Table), in contrast, it interacts with eighteen NPC subunits in axenic amastigotes (S1E Fig (blue and red squares) and S6 Table), including three hypothetical proteins. These proteins, LdBPK_091010.1, LdBPK_100580.1, LdBPK_281740.1, might be novel components of the NPC, as judged by the localization of their orthologs in *T. brucei* [39]. While these L-CKAPs span most NPC subcomplexes, they are significantly enriched in the outer ring (*,S7 Table and S1E Fig). This specific enrichment corroborates a tight, life-stage-dependent interaction between L-CK1.2-V5 and the nuclear pore in axenic amastigotes. Similarly to L-CK1.2-V5, in *Trypanosoma brucei*, TbCK1.2 interacts with TbNUP158, a member of the outer ring [52] and Hrr25, CK1 ortholog in *S. cerevisiae*, interacts with and phosphorylates NUP53 [53]. These findings shed light on the potentially distinct functional characteristics exhibited by L-CK1.2 in the two stages.

Overall and in line with other CK1 family members [5], these findings suggest a pleiotropic subcellular localization of L-CK1.2. To support this hypothesis, we next performed co-localization studies using L-CK1.2-V5 and organelle-specific marker proteins.

### *Leishmania* CK1.2 displays a pleiotropic localisation, consistent with that of its interaction partners

To determine L-CK1.2 localisation, we used the transgenic *L. donovani* Bob (*Ld*Bob) parasites carrying either the empty pLEXSY vector (‘mock’) or pLEXSY-CK1.2-V5-His_6_ (‘CK1.2-V5’[18],). Both strains were fixed with paraformaldehyde (PFA). L-CK1.2-V5 was detected as intense punctate staining in the cytoplasm and the flagellum of PFA-fixed promastigotes expressing L-CK1.2-V5 (Fig 3Aa) but not in the corresponding mock control, which only showed weak background fluorescence (Fig 3Ab). The sum of the fluorescence intensity in the body of CK1.2-V5 promastigotes was significantly higher than that of the mock parasites with a p-value below 0.0001 (****,S2A Fig), indicating that this punctate staining is specific to CK1.2-V5. Similar localisations were also observed for axenic amastigotes (Fig 3Ac). This pleiotropic localization aligns with the diverse functions attributed to this protein kinase family. However, in the absence of a signal peptide for specific targeting, CK1 family members may primarily depend on protein-protein interactions for their localization [17].

**Fig 3:**
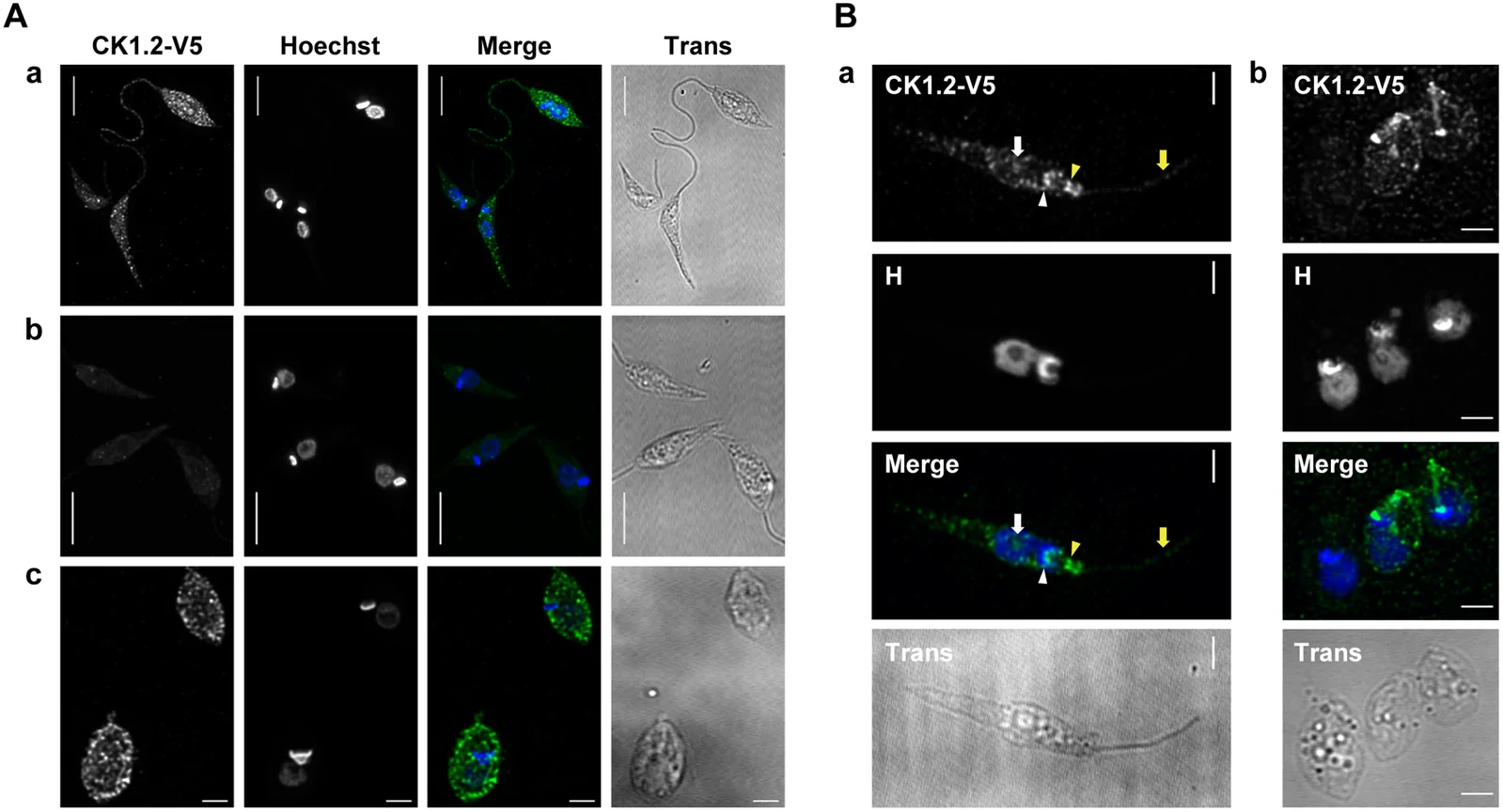
L-CK1.2-V5 displays a pleiotropic localisation in *Leishmania* parasites, consistent with that of its interaction partners. **(A)** IFA of *Ld*Bob pLEXSY-CK1.2-V5 (a) and *Ld*Bob pLEXSY (mock, b) promastigotes, fixed with PFA. The confocal images show the anti-V5 staining (CK1.2-V5 or V5, green), Hoechst 33342 staining (H, blue), a merge and the transmission image (Trans). Scale bar, 5 µm. The pictures are maximum intensity projection of the confocal stacks containing the parasites. (c) IFA of *Ld*Bob pLEXSY-CK1.2-V5 axenic amastigotes, fixed with PFA. The single channel images show CK1.2-V5 (green) and Hoechst 33342 (H, blue) signals, the merge and the transmission image (Trans). Scale bar, 2 µm. The pictures are maximum intensity projection of the confocal stacks containing the parasites. Confocal stacks were deconvolved and corrected for chromatic aberration. These images are representatives of at least 3 biological replicates. **(B)** IFA of LdBob pLEXSY-CK1.2-V5 promastigotes (a) and axenic amastigotes (b) obtained after detergent treatment followed by PFA fixation. Similar to (Fig 3A), except that the images are sum intensity projection of the confocal stacks containing the parasites. These images are representatives of at least 3 biological replicates.

As the important cytoplasmic staining of L-CK1.2-V5 might mask other more specific localisations, promastigotes were treated with 0,125% NP-40 to permeabilise membranes and facilitate the release of the cytoplasmic pool of L-CK1.2-V5, prior to PFA fixation and staining. The treatment conditions were selected after optimisation steps performed to minimise the impact of the detergent on the nucleus and kinetoplast morphology. We used organelle-specific markers for co-localisation with L-CK1.2-V5 and also to demonstrate the integrity of the organelle. We observed organelle-specific signals (i) adjacent to the kinetoplast (Fig 3Ba, CK1.2-V5 & merge, white arrowhead), (ii) in the flagellar pocket region and along the flagellum (Fig 3Ba, CK1.2-V5 & merge, yellow arrowhead and yellow arrow, respectively), as well as (iii) in the Hoechst-unstained region of the nucleus (Fig 3Ba, CK1.2-V5 & merge, white arrow). Similar localisations were observed in axenic amastigotes (Fig 3Bb, CK1.2-V5 & merge). These signals were reproducibly observed in all the samples that were analysed, including those fixed with methanol (as an example: 3 min fixation, S2B Fig), suggesting that the observed localisation is not a consequence of the fixation processes.

### CK1.2 is localised in the granular zone of the nucleolus and redistributed to the mitotic spindle during mitosis

Forty-eight L-CKAPs have a potential localisation to the nucleolus (S2 and S6 Tables). Most of L-CK1.2-V5 interacting partners in the nucleolus are involved in translation, including a wide range of 40S and 60S ribosomal proteins, as well as NOP56 [54]. L-CK1.2-V5 is also detected in the nucleolus as judged by Fig 3Ba (CK1.2-V5, white arrow), where CK1.2-V5 is detected in a Hoechst-unstained, sub-nuclear location. To confirm this result, the localisation of CK1.2-V5 in detergent-treated promastigotes was compared to that of L1C6 antibody, which recognises an unknown nucleolar protein [55]. As expected, the L1C6-targeted antigen was detected in the centre of the nucleolus corresponding to the dense fibrillar zone involved in rDNA transcription (Fig 4A, merged image, red staining) [56], confirming the structural integrity of the nucleolus. In contrast, CK1.2-V5 was detected at the periphery of the nucleolus, as dotted staining around L1C6 (Fig 4A, merged image, green staining). This region corresponds to the granular component of the nucleolus, involved in the last steps of rRNA processing and ribosome biogenesis [57]. Our results indicate that CK1.2 might be involved in rRNA processing rather than in rDNA transcription, which is consistent with its interaction with 40S and 60S ribosomal proteins, as well as the NOP56, a nucleolar protein involved in ribosome biogenesis, which orchestrates the correct assembly and the functioning of the box C/D small nucleolar ribonucleoproteins (snoRNPs) [54,58–60]. In addition to NOP56, L-CK1.2-V5 interacts with members of NOP56 complex such as Fibrillarin (box C/D snoRNPs), Hel67 (LSU assembly factors), 60S ribosomal L7 protein, GTP-binding nuclear protein rtb2 and potentially the heat shock protein DNAJ (Fig 4B, [58]). Our interactome provides additional evidence supporting the localization of L-CK1.2 to the nucleolus and suggests a role for L-CK1.2 in ribosome processing. This role is also consistent with the presence of L-CK1.2 in high molecular weight complexes (Fig 1A).

**Fig 4:**
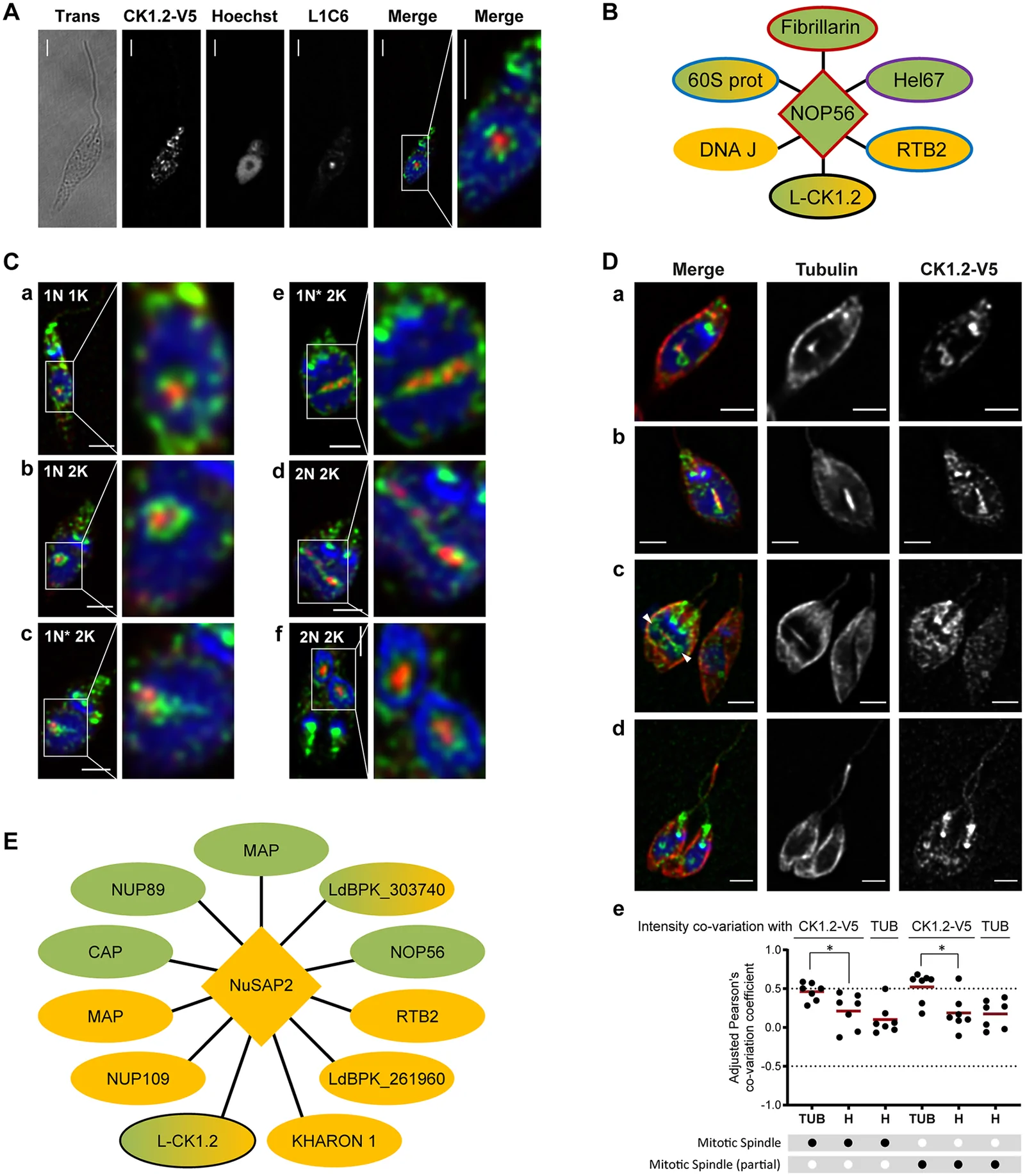
CK1.2-V5 localises in the nucleolus and to the mitotic spindle. (A) IFA pictures of LdBob pLEXSY-CK1.2-V5 promastigotes obtained after detergent treatment followed by PFA fixation. The images show the single channel images of the transmission image (Trans), CK1.2-V5, Hoechst 33342 (H, blue) and L1C6 signals (red) and merged image. The right panel show a magnification of the nucleus region. Scale bar, 2 µm. These pictures are single stacks extracted from deconvolved confocal stacks corrected for chromatic aberration. These images are representatives of at least 3 biological replicates. (B) Subunits of NOP56 complex implicated in ribosomal subunits biogenesis found in our dataset. Fill colors: Green, detected in promastigotes; Yellow, detected in axenic amastigotes; and Green/Yellow, present in both life stages. Border colors: Red, corresponds to box C/D snoRNPs; Purple, corresponds to LSU assembly factors; Blue, putative ribosome assembly factors; and Black, potential interaction found in this study. **(C)** IFA pictures of LdBob pLEXSY-CK1.2-V5 promastigotes obtained after detergent treatment followed by PFA fixation and stained with anti-V5 (CK1.2-V5) and anti-L1C6 (nucleolus, L1C6) antibodies. Confocal images representing sequential events of mitosis revealed different localisation patterns of L1C6 nucleolar marker and CK1.2-V5. (a – f) The images show the merged image containing CK1.2-V5 (green), Hoechst 33342 (H) (blue) and L1C6 (red) signals and a magnification of the nuclear region. N = nucleus, K = kinetoplast. Scale bar, 2 µm or 1 µm for magnified images. These pictures are single stacks extracted from deconvolved confocal stacks corrected for chromatic aberration. See also S3A Fig. These images are representatives of at least 3 biological replicates. (D) IFA pictures of LdBob pLEXSY-CK1.2-V5 promastigotes obtained after detergent treatment followed by PFA fixation and stained with anti-V5 and anti-α-tubulin antibodies. Sequential images of various stages of cell division (a – d) showing the single channel images for α-tubulin signals and the merged images showing CK1.2-V5 (green), H (blue) and α-tubulin (red) signals. Scale bar, 2 µm. These pictures are single stacks extracted from deconvolved confocal stacks corrected for chromatic aberration. See also S3B Fig. (e) Dot plots showing Pearson’s covariation coefficients for different combination of signals in the entire mitotic spindle region and in a reduced mitotic spindle region containing also CK1.2-V5 signal. Regions of the spindle containing both CK1.2-V5 and tubulin were selected to evaluate whether L-CK1.2-V5 localises to the spindle using Pearson’s covariation coefficients. For both regions, CK1.2-V5 signal was compared with α-tubulin (TUB) or Hoechst 33342 (H). The signal of TUB was also compared with H. Pearson’s covariation coefficients were measured from n = 7 different confocal stacks which were deconvolved and corrected for chromatic aberration with Huygens Professional software (*, < 0.05). The plot was generated with GraphPad Prism software and the mean values are represented with red bold segments. These images are representatives of at least 3 biological replicates. (E) Subunits of NuSAP2 complex, found in our dataset, implicated in chromosome segregation. Fill colors: Green, detected in promastigotes; Yellow, detected in axenic amastigotes; and Green/Yellow, present in both life stage. Border colors: Black, potential interaction found in this study.

The immunofluorescence experiment also unveils an additional function for nucleolar L-CK1.2. In dividing cells, CK1.2-V5 staining elongates from a wheel-shaped to a bar-shaped configuration that spans the entire length of the nucleus, following nucleolar elongation (Fig 4C, green staining, panels a-f, and S3A Fig). This is in contrast to the typical segregation pattern observed for nucleolar components exemplified by L1C6 antigen (Fig 4C, red staining, panels a-f, and S3A Fig [61]). The localization pattern of L-CK1.2-V5 bears a resemblance to that of the mitotic spindle, and we have evidence suggesting an interaction between L-CK1.2-V5 and α-tubulin, a component of the microtubules (S2 Table). To determine whether L-CK1.2-V5 is localised to the spindle during chromosome segregation, we asked whether L-CK1.2-V5 co-localises with tubulin, a component of the spindle. As shown in Fig 4D (merged panels a-e) and S3B Fig, CK1.2-V5 significantly co-localises with tubulin (*), and thus with the mitotic spindle in specific areas (*). During anaphase, CK1.2-V5 accumulates at each end of the elongated mitotic spindle (Fig 4Dc-merge panel and S3Bd Fig, white arrowheads), similarly to the twinfilin-like protein [62]. These findings suggest that *Leishmania* CK1.2 might be involved in chromosome segregation, similarly to TbCK1.2 [62,63]. Consistent with this hypothesis, Nucleus and spindle associated protein 2 (LdNuSAP2), was identified in L-CK1.2-V5 interactome [64] alongside eleven interacting partners of NuSAP2 (Fig 4E, [64]). LdNuSAP2 is a highly divergent ortholog of Saccharomyces ASE1/HumanPRC1/ArabidopsisMAP65 protein family that mediates spindle elongation. Further investigations will be required to ascertain the involvement of CK1.2 in the regulation of mitosis.

### CK1.2 localises to the basal body and the flagellum

Based on immunofluorescence analysis (Fig 3Ba, white arrowhead) and the GO enrichment analysis of cellular component (ciliary basal body and microtubule organising center, Fig 2B), 12 L-CKAPs (S6 Table) are predicted to locate to (i) the basal body that regulates flagellum duplication, (ii) the tripartite attachment complex (TAC) that links the segregation of the kinetoplasts to that of the basal bodies, or (iii) the transition fibers (TF), critical for flagellum assembly. Most of these interacting proteins were identified either in axenic amastigotes or in both AxAMA and PRO, none were specific of PRO. Our data suggest that ciliary basal body and MTOC are GO term significantly enriched in axenic amastigote (**, Fig 2B and S7 Table). Several of these L-CKAPs are linked to the regulation of cell division and/or cytokinesis, such as *Leishmania* AKB1 or KHAP2 [13,65,66], while other are members of the Tripartite Attachment Complex (TAC) such as TAC60 [67]. To determine whether L-CK1.2 localizes to these organelles, we selected two markers, centrin-4 to investigate the potential localisation of CK1.2-V5 to the basal body (Fig 5A, CEN, white arrow) and to the bilobe structure (Fig 5A, CEN, yellow arrow); as well as IFT172 to investigate the potential localisation of CK1.2-V5 to the transition fibers (Fig 5A, IFT172) [68–70]. As shown by Fig 5A panel a and by the mean Pearson correlation coefficient below 0.5 (mPc = 0.27 ± 0.164, Fig 5B), CK1.2-V5 does not co-localise with the kinetoplast DNA. Instead, it significantly co-localises with centrin-4 to the basal bodies (Figs 5A panel a, white arrows and mPc of 0.741 ± 0.050, ***, and 5B) and to the bilobe structure (Figs 5A panel a, yellow arrow and mPc of 0.47 ± 0.074, ***, and 5B); and with IFT172 to the transition fibers, as judged by Fig 5A panel b, white arrow and confirmed by an mPc of 0.805 ± 0.06 (***, Fig 5B). These findings suggest that L-CK1.2 might be involved with flagellum, bilobe structure and kinetoplast duplication as well as be loaded onto the flagellum [71]. Consistent with this finding, we confirmed the localisation of CK1.2-V5 to the axoneme (IFT172, Fig 5C, Merge and 3D-view), but not to the paraflagellar rod (PFR2, Fig 5D, panel 3). Noticeably, the abundance of L-CK1.2-V5 in the flagellum is lower than that in the body of the parasite, which is consistent with the existence of a filter that regulates selective uptake into the flagellum [72]. The flagellar localisation of L-CK1.2-V5, which aligns with published proteomic data from *T. brucei* and *Leishmania mexicana* flagellum [73,74], as well as the identification of 19 flagellar L-CKAP (S5 Table, section flagellum [74],) indicates a potential function for L-CK1.2 in the flagellar biology*.* Additionally, L‑CK1.2-V5 phosphorylates HSP90 at Ser‑289; blocking this modification, by introducing an S289A mutation, produces mutant parasites whose flagella are shortened by roughly 50% [20]. This finding suggests a regulatory role of L-CK1.2 in flagellar length.

**Fig 5:**
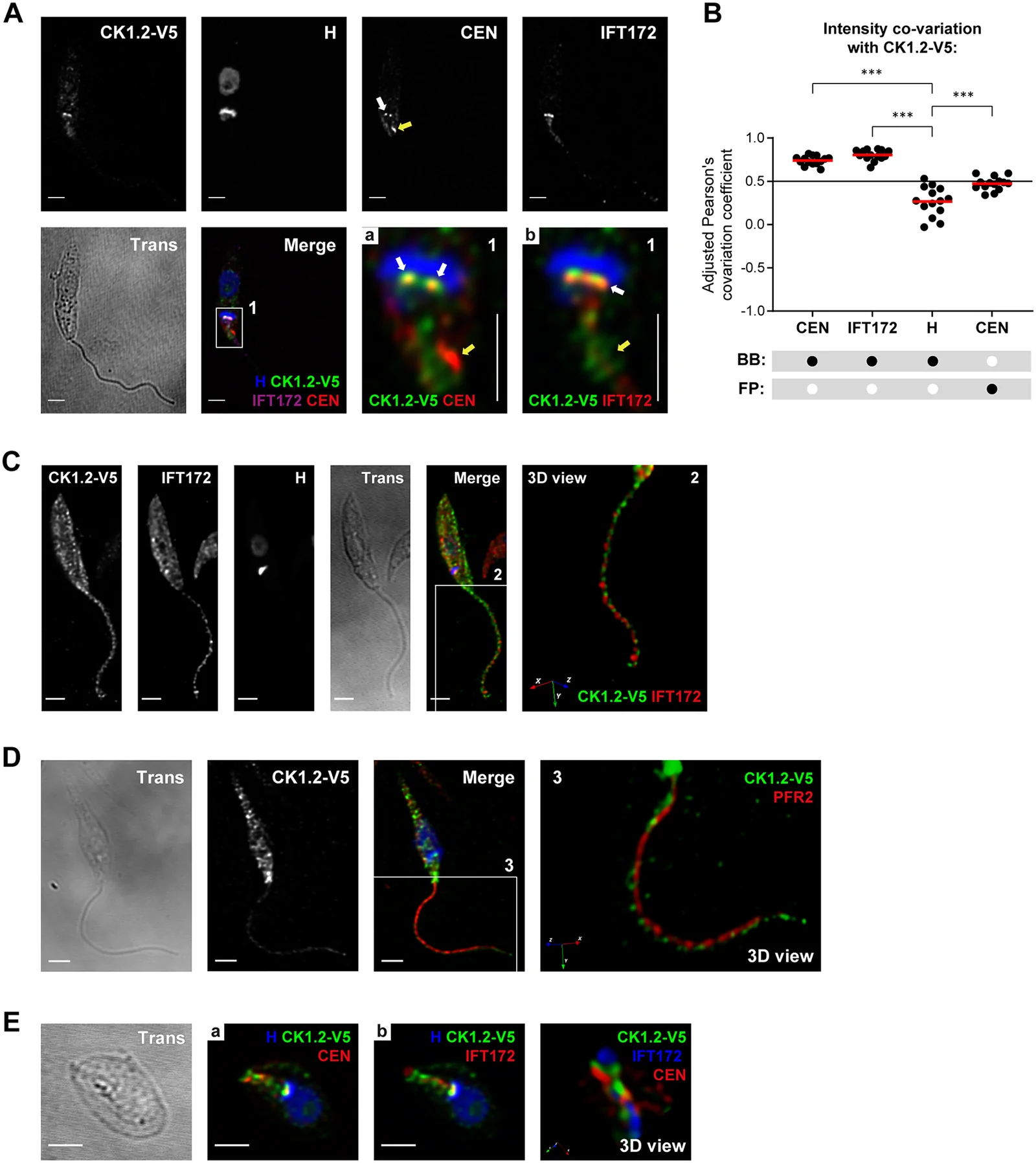
CK1.2-V5 is localised to the basal bodies and the flagellum. (A) IFA of LdBob pLEXSY-CK1.2-V5 promastigotes obtained after detergent treatment followed by PFA fixation. Single channel images of the CK1.2-V5, Hoechst 33342 (H), centrin 4 (CEN) or IFT172 signals and the transmission image (Trans). The white arrows highlight the basal bodies and the yellow arrow the bilobe region. The merge panel shows CK1.2-V5 signal (green) merged with H (Hoechst 33342, blue), CEN (centrin 4, red) and IFT172 (purple). The two right panels show a magnification of region 1 with H signal (blue) merged with (a) CK1.2-V5 (green) and CEN (red) and (b) IFT172 (red) and CK1.2-V5 (green) signals. Scale bar, 2 µm or 1 µm for magnified images. These pictures are single stacks extracted from deconvolved confocal stacks corrected for chromatic aberration. (B) Dot plots showing Pearson’s covariation coefficients in the basal body (BB) or the flagellar pocket (FP) regions for different combination of signals. Pearson’s covariation coefficients were measured from n = 14 different confocal stacks which were deconvolved and corrected for chromatic aberration with Huygens Professional software. The plot was generated with GraphPad Prism software and the mean values are represented with red bold segments. *** (0.001). (C) IFA of LdBob pLEXSY-CK1.2-V5 promastigotes fixed by PFA and stained with the anti-V5 and anti-IFT172 antibodies. The left panels display the single channel images of the CK1.2-V5, IFT172, Hoechst 33342 (H) signals, the transmission image (Trans), and a merge of CK1.2-V5 (green), IFT172 (red) and H (blue) signals. The right panel shows a 3D-reconstruction (3D view) of the flagellum (image 2), with CK1.2-V5 signal (green) merged with IFT172 (red). (D) IFA of LdBob pLEXSY-CK1.2-V5 promastigotes obtained after detergent treatment followed by PFA fixation and staining with the anti-V5 and anti-PFR2 antibodies. The following images show the transmission image (Trans), CK1.2-V5, and finally the merge of CK1.2-V5 (green), PFR2 (red) and H (blue) signals. (image 3) 3D-reconstruction (3D view) of the flagellum (white square in (merge)), showing CK1.2-V5 signal (green) merged with PFR2 (red). Scale bars, 2 µm for 2D images. Pictures in (E) and (D, left panels) are single stacks extracted from deconvolved confocal stacks corrected for chromatic aberration. All confocal stacks containing the parasite were used for pictures (C, image 2 and D image 3). (E) IFA of LdBob pLEXSY-CK1.2-V5 axenic amastigotes obtained after detergent treatment followed by PFA fixation. The merge images show CK1.2-V5 (green), Hoechst 33342 (H, blue), centrin 4 (CEN, red) or IFT172 (red) signals and the transmission image (Trans). Scale bar, 2 µm. These pictures are single stacks extracted from deconvolved confocal stacks corrected for chromatic aberration. Panel a shows a merged of CK1.2-V5, Hoechst 33342 (H, blue) and centrin 4 (CEN, red); Panel b shows CK1.2-V5, Hoechst 33342 (H, blue), and IFT172 (cyan) signals; 3D-reconstruction of the flagellar pocket region and its neck from image (Figs 5Ea and 5Eb), which has been rotated. All confocal z-stacks containing the parasite were used for the 3D view. All the images presented in Fig 5 are representatives of at least 3 biological replicates.

Based on the literature and our findings, *Leishmania*, alongside *Chlamydomonas reinhardtii* and *Trypanosoma brucei*, is one of the few eukaryotes exhibiting flagellar localization of CK1 [75]. In axenic amastigotes, however, CK1.2-V5 does not colocalise with IFT172, but instead localises around the flagellum (Fig 5E, panel b and 3D view). These findings suggest that the role of CK1.2 in the flagellum is more likely associated with motility or flagellum assembly than in sensing.

### *Leishmania* CK1.2 interacts with host proteins.

Several lines of evidence suggest that L‑CK1.2 is exported from the parasite in extracellular vesicles (EVs) and can phosphorylate host substrates, thereby contributing to the parasite ability to subvert macrophage biology [15,19,22]. Because L‑CK1.2-V5 is expressed at low levels in both promastigotes and axenic amastigotes [18], conventional immunoprecipitation (IP) of the kinase from infected macrophages would not yield sufficient material for a comprehensive interactome analysis. Furthermore, the amount of host‑localised pool of L‑CK1.2-V5 that can be harvested from *Leishmania*-infected BMDMs is well under the threshold needed for a robust IP‑MS analysis. To circumvent these limitations, we adopted an *in‑vitro* pull‑down strategy that has previously been validated for mapping host–pathogen protein interactions in *Mycobacterium* [76].

Recombinant L-CK1.2-V5, produced in *E. coli*, was incubated overnight with macrophage protein lysates and immunoprecipitated using an anti-V5 antibody [18,76] to identify the host L-CK1.2-V5-associated proteins (L-CKAP*host*) by mass spectrometry analysis. Two controls were added, an empty vector, and a control-thioredoxin (an empty plasmid expressing thioredoxin-V5), as recombinant L-CK1.2-V5 is fused to thioredoxin at the N-terminus to improve its solubility. Three independent immuno-precipitations were performed using a mouse monoclonal anti-V5 antibody. The proteins were run on SDS-PAGE and the binding partners revealed by quantitative label-free mass spectrometry (Fig 6Aa-c). We identified 64 L-CKAP*host* in at least two replicates with at least two distinct peptides detected (S8 Table). L-CK1.2-V5 interacts with a wide range of host protein classes, including chaperones and transporters, similar to the L-CKAPs identified in *Leishmania* (Fig 6B). This finding suggests that L-CK1.2 may perform similar functions in both the parasite and its host cell. The GO term analysis for Biological Process (S4A Fig) revealed a significant enrichment for the terms ‘purine biosynthetic process’, ‘glycolytic process’ (ATP-dependent 6-phosphofructokinase, Pfkl and Glyceraldehyde-3-phosphate dehydrogenase, Gapdh), ‘mitochondrial electron transport’ (mitochondrial ATP synthase subunit, Atp5f1a, Atp5f1c and Atp5po, Cytochrome b-c1 complex subunit 8) and ‘mitochondrial membrane respiratory chain’ (Ndufa1, Ndufa10). We also identified an enrichment of GO terms associated with infection, such as “immune response activation,” “apoptotic cell death,” and “response to stress.”

**Fig 6:**
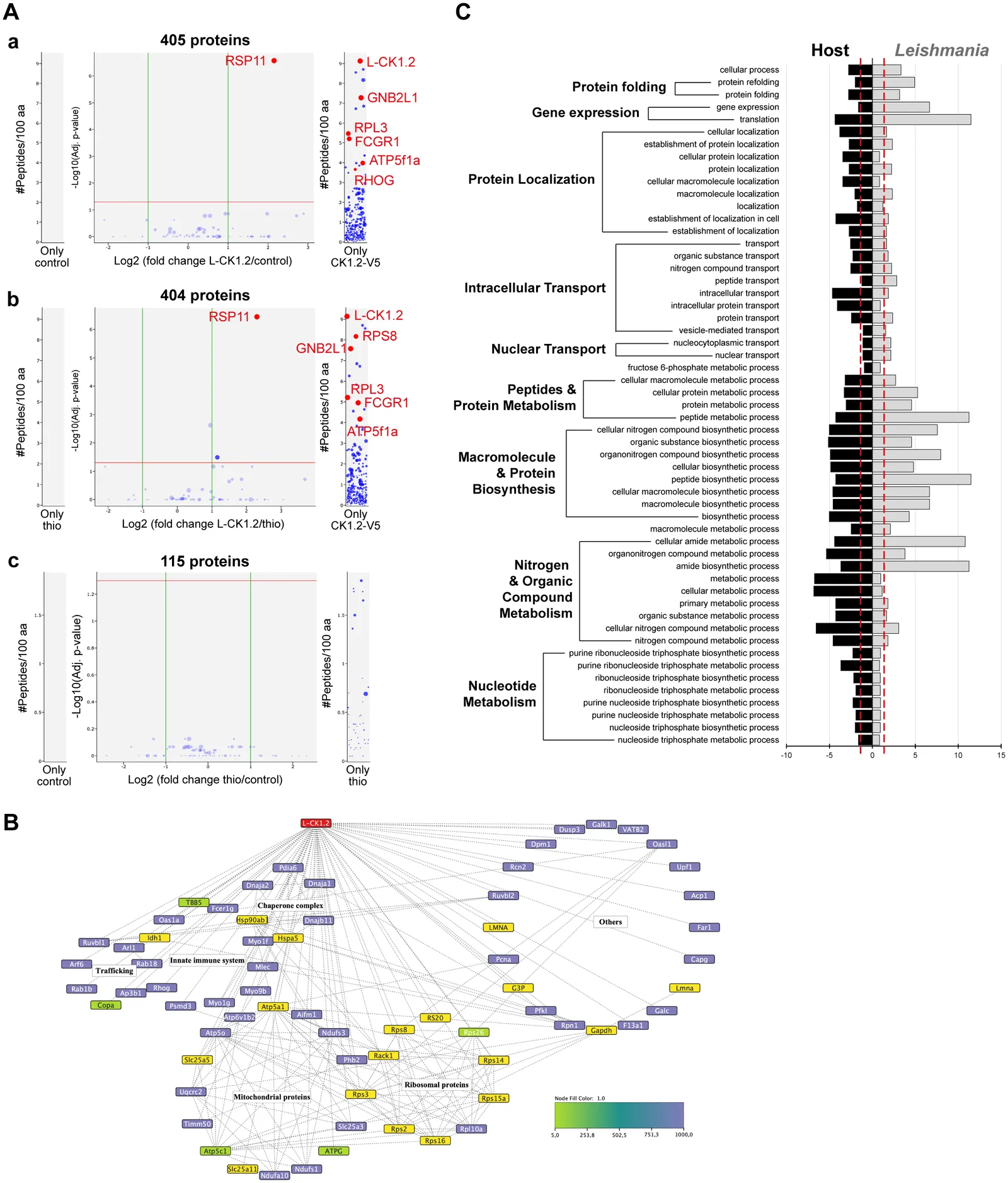
L-CK1.2-V5 interacts with macrophage proteins. (A) The recombinant L-CK1.2-V5, the control-thioredoxin and the control were incubated overnight with BMDM protein lysates and immunoprecipitated using an anti-V5 antibody. The proteins were eluted by two successive elutions with glycine elution buffer (elution 1 and 2) followed by one elution with NuPAGE loading buffer (elution 3). Proteins from elutions 1 and 3 were used for MS analysis. Volcano plots representing the changes in protein abundance of L-CK1.2-V5 vs control (a), of L-CK1.2-V5 vs control thioredoxin (b) and of control thioredoxin vs control (c), including all the proteins identified with 1 peptide in at least 1 replicate. Proteins highlighted in red are among those with at least 2 distinct peptides in at least 2 replicates. (B) Graphical representation of the affinity purification merged with the string app to visualise protein-protein interactions for host proteins using the cytoscape app. The classification in different categories is based on GO terms enrichment. The colour of the nodes represents the Fold change (see the legend on the figure). (C) GO term enrichment analysis for the ‘biological process’ ontology, identifying terms common to both the host cell and *Leishmania donovani*, was performed using TriTrypDB (see also S10 Table). Data are presented as the negative base-10 logarithm of the Benjamini–Hochberg false discovery rate (–log_10_ FDR). For visualization purposes, the log-transformed FDR values for *L. donovani* were inverted to positive values. The red vertical lines indicate the significance threshold of –log_10_(0.05) ≈ −1.3 / 1.3.

Notably, many of the biological processes enriched in the interactome are also present in the L-CK1.2-V5-related host substratome we previously published (S4B Fig, S9 Table [19]). Because of its potential dual role, L-CK1.2 has the ability to function in both the parasite and the macrophage. To determine whether L-CK1.2 targets similar pathways in both organisms, we analysed the intersection between biological processes associated with L-CK1.2-V5 in the parasite and the host cell by combining the L-CKAP*host* and substratome datasets (this work and [19], Fig 6C, S10 Table). Several biological processes were conserved in both organisms, which might represent the core of L-CK1.2 functions: (i) biosynthetic metabolic processes; (ii) translation; (iii) transport; (iv) peptide and protein metabolism (Fig 6C). Another characteristic, conserved in both organisms, is protein folding, through the interaction of L-CK1.2-V5 with chaperone proteins (Fig 6B). We have shown that L-CK1.2-V5 interacts with *Leishmania* chaperone proteins, including HSP70s, HSP60, DNAJs, HSP110. Similarly in the macrophage, L-CK1.2-V5 interacts with DNAJs, Hspa5 (Hsp70) and Hsp90. Interestingly, while *Leishmania* HSP90 is phosphorylated by L-CK1.2-V5 [20], only host Hsp90 seems to interact with L-CK1.2-V5.

Overall, although this experiment was conducted *in vitro* and may therefore capture interactions that do not occur under physiological conditions, it generated a comprehensive catalogue of potential host interacting partners of L‑CK1.2. Notably, four proteins, Rcn2, Myo1f, Myo9b, and Hspa5, were identified in both L‑CK1.2 host interactome and substratome, making them particularly attractive targets [19]. Future functional studies will be necessary to corroborate these results by performing co-immunoprecipitations with selected host targets to validate L-CK1.2 interaction in macrophages.

## Discussion

Currently, little is known about *Leishmania* CK1.2, despite its crucial role in parasite survival and its validation as a drug target [13,18,30]. This might be linked to the challenges associated with studying an essential signalling kinase. Our findings shed light on the versatile and pivotal role of L-CK1.2, demonstrated by its diverse subcellular localization and broad spectrum of interacting partners. Our data demonstrate that L-CK1.2 participates in multiple high-molecular-weight complexes within the parasite. This observation is supported by L-CK1.2 interaction with components of large complexes, including ribosomal proteins and the AP2 complex. The diversity and abundance of L-CK1.2 interacting partners are consistent with the presence of Low Complexity Regions within its C-terminal domain. LCRs are known to be enriched in highly connected proteins, such as signalling kinases, where they facilitate protein-protein interactions [77]. Notably, LCRs are also conserved in the C-terminal domains of all human CK1 isoforms (S5 Fig, SMART database [78],). Since L-CK1.2 lacks a signal peptide or other canonical targeting domains [12,18], these interactions appear crucial to reach its specific subcellular localisations. In humans, Fulcher *et al.* demonstrated that FAM83 family members act as privileged partners for CK1s, ensuring their precise subcellular localization (Fulcher et al., 2018). Although *Leishmania* lacks orthologs of the FAM83 family, we hypothesize that a distinct set of proteins fulfils this role, mediating the subcellular localization of L-CK1.2 through similar interaction mechanisms. This spatial coordination is particularly important given that L-CK1.2 is constitutively active [18].

We identified hundreds of potential L-CK1.2 interactors involved in diverse pathways, ranging from nuclear transport to ribosomal biogenesis. This broad interactome aligns with the pleiotropic subcellular localization observed for L-CK1.2. While the specific partners governing L-CK1.2 trafficking remain to be fully elucidated, our data allow us to infer several key functions for this kinase in the parasite, which will be the subject of future investigation. **1)** Cell Cycle. L-CK1.2 localizes to the basal body, nucleolus, mitotic spindle, structures central to cell division [69] and interacts with several known cell cycle regulators, such as AKB1 (the associated kinase of Tb14-3-3 [65],). Moreover, knockdown of TbCK1.2, L-CK1.2 ortholog, leads to multinucleation, likely due to cytokinesis defects [63,79], affects basal body duplication and kDNA scission [79]. Overall, these findings suggest a role in cell cycle control for L-CK1.2. **2)** Nucleolar processes. The detection of CK1.2 in the nucleolus points to roles in both ribosomal processing and chromosome segregation. Regarding ribosome biogenesis, the localization of L-CK1.2 in the granular zone of the nucleolus, indicate that CK1.2 might be involved in rRNA processing rather than in rDNA transcription [57]. This is consistent with its interaction with 40S and 60S ribosomal proteins, as well as with members of the NOP56 complex [54,58–60]. This potential role contrasts with that of yeast and human CK1, which are primarily involved in the cytoplasmic maturation of pre-40S ribosomes [80,81]. Nucleolar L-CK1.2 co-localizes with tubulin from the onset of mitotic spindle assembly through its elongation, which aligns with evidence that the nucleolus serves as a hub for spindle elongation factors [62,64]. This colocalization is also consistent with L-CK1.2 interaction with LdNuSAP2. This protein is detected in the central spindle during metaphase and anaphase in *T. brucei*, as well as ten other proteins within the NuSAP2 complex [64]. These findings suggest that L-CK1.2 may be yet another nucleolar protein that dynamically relocates to the mitotic spindle during mitosis, similarly to TbNOP86 [82] and LdTWF, an actin-binding protein controlling spindle elongation in *Leishmania* [62]. **3)** Flagellar motility. L-CK1.2 localizes to the flagellum. Along with *Chlamydomonas reinhardtii* and *Trypanosoma brucei*, *Leishmania* represents one of the few eukaryotes exhibiting flagellar localization of CK1 [75]. This localization is supported by the interaction of L-CK1.2 with several flagellar-related proteins, including a dynein light chain. In *C. reinhardtii*, CK1 regulates dynein activity and flagellar motility by phosphorylating the Inner Dynein Arm I1 Intermediate Chain 138 [83,84]. These findings suggest that, in addition to its involvement in flagellum duplication, L-CK1.2 may also be involved in motility [75]. Supporting this hypothesis, phosphorylation of Hsp90 on Ser289 by L-CK1.2 is important for parasite flagella length [20,21].

Despite the limitations inherent to the incomplete annotations of *Leishmania* genome, we observed distinct differences in the L-CK1.2 interactome between promastigotes and axenic amastigotes. By cross-referencing our data with the total proteome profile obtained by Pescher *et al.* [85], we found that 50% of promastigote-specific and 24% of axenic amastigote-specific interactors are proteins mostly expressed in those respective stages. This suggests that differential protein availability, likely regulated by environmental cues such as pH and temperature, accounts for a significant fraction of the stage-specific interactions. For instance, the enrichment of ribosome biogenesis factors in promastigotes and vesicle transport proteins in splenic amastigotes [85] mirrors our GO enrichment results. However, the case of nucleoporins presents a more complex picture. Although Pescher *et al.* noted a stage-specific trend for nucleoporins, the statistical significance was weak. Therefore, we cannot definitively attribute the stage-specific binding of L-CK1.2 to nucleoporins solely to protein abundance. It is equally plausible that L-CK1.2 exhibits a regulated affinity for nucleoporins or interacts specifically with cargo (proteins or mRNAs) transiting through the nuclear pore complex in a stage-dependent manner. The remaining stage-specific interactors may result from intrinsic changes in L-CK1.2 affinity. Given that elevated temperature (37°C) is known to modulate L-CK1.2 enzymatic activity [18], it is plausible that thermal stress also alters its protein binding affinity. Further experiments are required to test this hypothesis.

We identified host biological processes that may be regulated by L-CK1.2 through interactions with host proteins. Despite the inherent limitations of *in vitro* assays, which can yield non-physiological interactions, this study provides a comprehensive list of candidate host proteins that interact with L-CK1.2 and first insights into its potential role during infection. Although additional studies are needed to confirm these functions and elucidate these mechanisms, our study provides a comprehensive catalogue of high-priority candidates to guide future research. Protein class ontology analysis reveals that the host dataset aligns well with the parasite dataset, which was derived under more physiologically relevant conditions. Future research will focus on validating these interactions in more physiological contexts, such as a cellular model or infected macrophages. Many of the enriched host processes in our dataset, including cell death, immune response, and transport, are known to be modulated during *Leishmania* infection [86–89]. The two main host biological processes (BPs) targeted by L-CK1.2 *in vitro* are ‘purine biosynthesis’ (which is closely linked to the ‘glycolytic process’) and ‘mitochondrial electron transport’. Collectively, these BPs suggest a potential role for L-CK1.2 in regulating host respiratory metabolism. This finding aligns with recent data from Zhang *et al.*, who demonstrated that *Leishmania amazonensis*-infected macrophages shift toward aerobic glycolysis for ATP production [90]. Furthermore, we identified numerous proteins associated with mitochondrial electron transport, specifically subunits of Complex I (NADH dehydrogenase), including Ndufa10, Ndufs1, and Ndufs3. These interactions support the model of *Leishmania*-mediated mitochondrial modulation for survival and bioenergetics [91], and parallel the known role of human CK1δ/ε in regulating electron transport chain Complexes I and IV [92]. Finally, we identified a substantial number of host ribosomal proteins interacting with L-CK1.2, mirroring our findings with *Leishmania* ribosomal proteins. This conservation supports a role for L-CK1.2 in regulating translation and aligns with the *Leishmania* reprogramming host translation [93].

The essential nature of L-CK1.2, its secretion via extracellular vesicles and its ability to phosphorylate host proteins suggest a dual role in the parasite and in the host (this work [15,19,22],). Unlike pathogens that hijack host CK1, *Leishmania* secretes its own, likely to functionally mimic mammalian counterparts. We hypothesize that during *Leishmania* infection, the functions targeted by L-CK1.2 might be attenuated by the inhibition or the reduced abundance of the host CK1s [94]. This is supported by the exceptional conservation of L-CK1.2 catalytic and C-terminal domains across human-infecting species [18], a trait driven by strong selective pressure from the mammalian host [12,18]. Consequently, L-CK1.2 targets conserved pathways in both organisms, including translation, transport, and protein folding. Notably, its interaction with heat shock proteins in both compartments suggests a critical role in adapting to environmental stress. We, and others, clearly showed a strong link between L-CK1.2 and heat shock proteins in the parasite. L-CK1.2 is in complex with the *Leishmania* HSP70s and phosphorylates HSP23, P23 and HSP90, (this study [20,21],). Noticeably, while *Leishmania* HSP90 was not identified among the L-CKAPs, host Hsp90ab1 was identified among the L-CKAP*host* (this work and [19]), suggesting that its HSP90-related functions may differ between the parasite and the macrophage, potentially resembling those of mammalian CK1s more closely. The importance of L-CK1.2 interaction with *Leishmania* HSP70 is unclear.

## Conclusion

Studying signalling kinases is inherently challenging, as their impairment affects multiple pathways, making it difficult to dissect precise regulatory mechanisms. Our study addresses this challenge by identifying specific interacting partners of L-CK1.2, which will be instrumental in elucidating its diverse functions in the parasite. These findings, together with previous studies, underscore CK1.2 as a pivotal enzyme essential for *Leishmania* survival and suggest its requirement for adaptation to the host cell environment [18,19]. We propose that this parasite-encoded serine/threonine kinase exerts dual functionality, acting in cis within the parasite itself while simultaneously operating in trans within the macrophage. In doing so, CK1.2 might establish itself as a bidirectional signalling hub that coordinates communication between pathogen and host. This dual functionality underscores the exceptional potential of L-CK1.2, positioning it as a unique therapeutic target [95–97]. Further characterization is now required to confirm these specific interactions and decipher their precise mechanisms of action. Given the close relationship between *Leishmania* CK1.2 and its orthologs in *Trypanosoma brucei* (TbCK1.2), *Trypanosoma cruzi* (TcCK1.2), *Toxoplasma gondii* (TgCK1α), and *Plasmodium falciparum* (PfCK1) [18], our findings are likely to have broader implications for other parasitic infections.

## Supporting information

S1 Fig L-CK1.2 interacts with a diverse range of *Leishmania* proteins. (**A**) Distribution of L-CK1.2 across SEC fractions. Fifteen microliters of each fraction collected from the SEC run (see Figure 1A) were analyzed by Western blot, using an anti-V5 antibody. The blot shown is representative of three independent biological replicates. (**B**) SEC profiling of recombinant L-CK1.2 and molecular weight markers. 500 μg of recombinant V5-tagged L-CK1.2 was separated on a Superose 6 Increase 10/300 GL column. Fractions (200 μL) were collected, and 15 μL of each fraction was resolved by SDS-PAGE under denaturing conditions and probed with an anti-V5 antibody (bottom panel). The presence of recombinant L-CK1.2 in the 7.8 mL fraction, which correspond to the void fraction (Blue dextran), might be due to aggregate. Peak fractions were quantified using a Bio-Rad ChemiDoc imaging system. Data represent the average of two biological replicates. The elution profile of L-CK1.2 was compared against standard protein markers (MW: Molecular Weight). (**C**) Analysis of the endogenous L-CK1.2 oligomeric state. Six micrograms of total protein lysates extracted from promastigotes were separated by native PAGE and subjected to Western blotting using the anti-L-CK1.2 antibody (SY3535). The blot shown is representative of three biological replicates. (**D**) Network visualization of L-CK1.2 protein–protein interactions. Affinity purification data were merged with known interactions from the STRING database and visualized using Cytoscape. Nodes represent proteins; edges represent interactions. Blue nodes with zigzag lines indicate interactions specific to promastigotes (PRO), while light blue nodes with straight lines indicate those specific to axenic amastigotes (AxAMA). Red lines denote interactions identified in this study, whereas black lines represent interactions retrieved from the STRING database. Node size corresponds to the fold-change ratio of L-CK1.2 versus mock control (ranging from 2 to 1000), reflecting peptide abundance. (**E**) Schematic of L-CK1.2 association with nuclear pore complexes (NPCs). The diagram is adapted from Obado et al. {Obado, 2016 #526}. Proteins identified exclusively in axenic amastigotes are outlined in red; those identified in promastigotes are outlined in blue. The grey square with a red border indicates a novel protein component added to the original schematic, based on findings from this study.
(PDF)

S2 Fig L-CK1.2 has a pleiotropic localisation in the parasite. (A) Analysis of different parameters extracted from ROI of the promastigotes parasite bodies. Scatter dot plots showing the sum of fluorescence intensity (for the V5 signal) of CK1.2-V5-expressing (quadruplicate) or mock control (duplicate) cell lines. The red line corresponds to the mean intensity. (B) IFA of LdBob pLEXSY-CK1.2-V5 (A) and LdBob pLEXSY (mock, B) promastigotes, fixed in ice-cold methanol for 3 minutes and stained with anti-V5 antibody to detect CK1.2-V5 localisation. The epifluorescence images were acquired under the same conditions and show the anti-V5 staining (CK1.2-V5 or V5), Hoechst 33342 staining (H), a merge of the anti-V5 (green) and H (red) signals and the transmission image (Trans). Scale bar, 5 μm. The pictures are maximum intensity projection of the z-stacks containing the parasites. All the images presented in Figure S2B are representatives of at least 3 replicates.
(PDF)

S3 Fig CK1.2 localises to the nucleolus and the mitotic spindle in the parasite. (A) IFA pictures of LdBob pLEXSY-CK1.2-V5 promastigotes obtained after detergent treatment followed by PFA fixation and stained with anti-V5 (CK1.2-V5) and anti-L1C6 (nucleolus, L1C6) antibodies. Confocal images representing sequential events of mitosis revealed different localisation patterns of L1C6 nucleolar marker and CK1.2-V5. (a – f) The images correspond to the transmission (Trans), the merged containing CK1.2-V5 (green), Hoechst 33342 (H) (blue) and L1C6 (red) signals. The following four images show a magnification of the nuclear region with the merged and single channel images. N = nucleus, K = kinetoplast. Scale bar, 2 µm or 1 µm for magnified images. These pictures are single stacks extracted from deconvolved confocal stacks corrected for chromatic aberration. (B) IFA pictures of LdBob pLEXSY-CK1.2-V5 promastigotes obtained after detergent treatment followed by PFA fixation and stained with anti-V5 and anti-α-tubulin antibodies. Sequential images of various stages of cell division (a – e) showing the single channel images for CK1.2-V5, H and α-tubulin signals, the merged images showing CK1.2-V5 (green), H (blue) and α-tubulin (red) signals, and the transmission image (Trans). Scale bar, 2 µm. These pictures are single stacks extracted from deconvolved confocal stacks corrected for chromatic aberration. All the images presented in Figure S3 are representatives of at least 3 replicates.
(PDF)

S4 Fig L-CK1.2 interacts with macrophage proteins. (A) GO ‘biological process’ enrichment network for the L-CKAP*Host* dataset. The enrichment map was generated using g:Profiler with the g:SCS multiple testing correction algorithm. In the network, node size corresponds to the gene set size (number of genes per term), node color indicates the statistical significance (p-value), and label font size is scaled according to the cluster size. (B) GO enrichment for ‘biological process’ terms shared between host-derived L-CKAP (this study) and the L-CK1.2 host substratome [19]. Enrichment analysis was performed using TriTrypDB. Significance is represented as the negative Log10 of the Benjamini-Hochberg False Discovery Rate.
(PDF)

S5 Fig Position of the LCR in human CK1s. Cartoon representing the low complexity region (LCR, dark grey) on the protein sequence (light grey) of human CK1. CK1A (P48729), CK1D (P48730), CK1E (P49674), CK1G1 (Q9HCP0), CK1G2 (P78368) and CK1G3 (Q9Y6M4).
(PDF)

S1 Table List of all quantified proteins in promastigotes and axenic amastigotes. (XLSX)

S2 Table List of potential L-CKAPs in promastigotes and axenic amastigotes. Selected proteins on the following criteria: proteins identified with 2 or more distinct peptides, a fold change ratio CK1.2-V5/mock above 2 and a p-value below 0.05 in at least two replicates.
(XLSX)

S3 Table GO term enrichment in protein class for promastigotes and axenic amastigotes L-CKAPs. (PDF)

S4 Table GO term enrichment in biological process for promastigotes and axenic amastigotes L-CKAPs. (PDF)

S5 Table Differential Biological process GO term enrichment in promastigote and axenic amastigote L-CKAPs. In grey are the GO term below the FDR threshold of 0.05.
(PDF)

S6 Table Ontology enrichment for Cellular Component of the promastigotes and axenic amastigotes L-CKAPs. (PDF)

S7 Table Differential Cellular Component GO term enrichment in promastigote and axenic amastigote L-CKAPs. In grey are the GO term below the FDR threshold of 0.05 [98].
(PDF)

S8 Table List of potential L-CKAP*host* in murine BMDM macrophages. Sheet all data: List of all quantified proteins in promastigotes and axenic amastigotes. CK1.2 binding partners: Selected proteins followed the following criteria: proteins identified with 2 or more distinct peptides, a fold change ratio CK1.2-V5/mock above 2 and a p-value below 0.05 in at least two replicates.
(XLSX)

S9 Table GO term enrichment for biological process shared between host-derived L-CKAP and the L-CK1.2 host substratome. (PDF)

S10 Table Common GO term enrichment of biological process in *Leishmania* and macrophage. (PDF)
