# Supplementary material for: Characterization of the dual functions of Leishmania CK1.2 in both the parasite and the macrophage
Source: PLoS One. 2026 Oct 5;21(10):e0356818. doi: 10.1371/journal.pone.0356818 (PMC13638044; doi:10.1371/journal.pone.0356818)

Figure S1

A

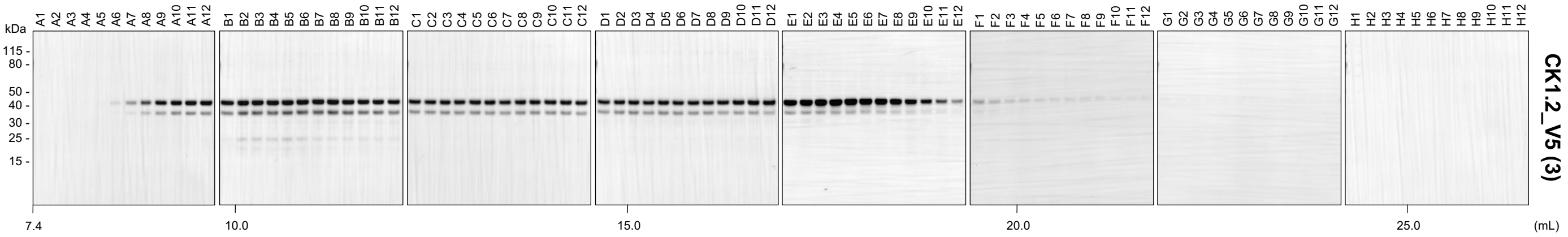

B

a

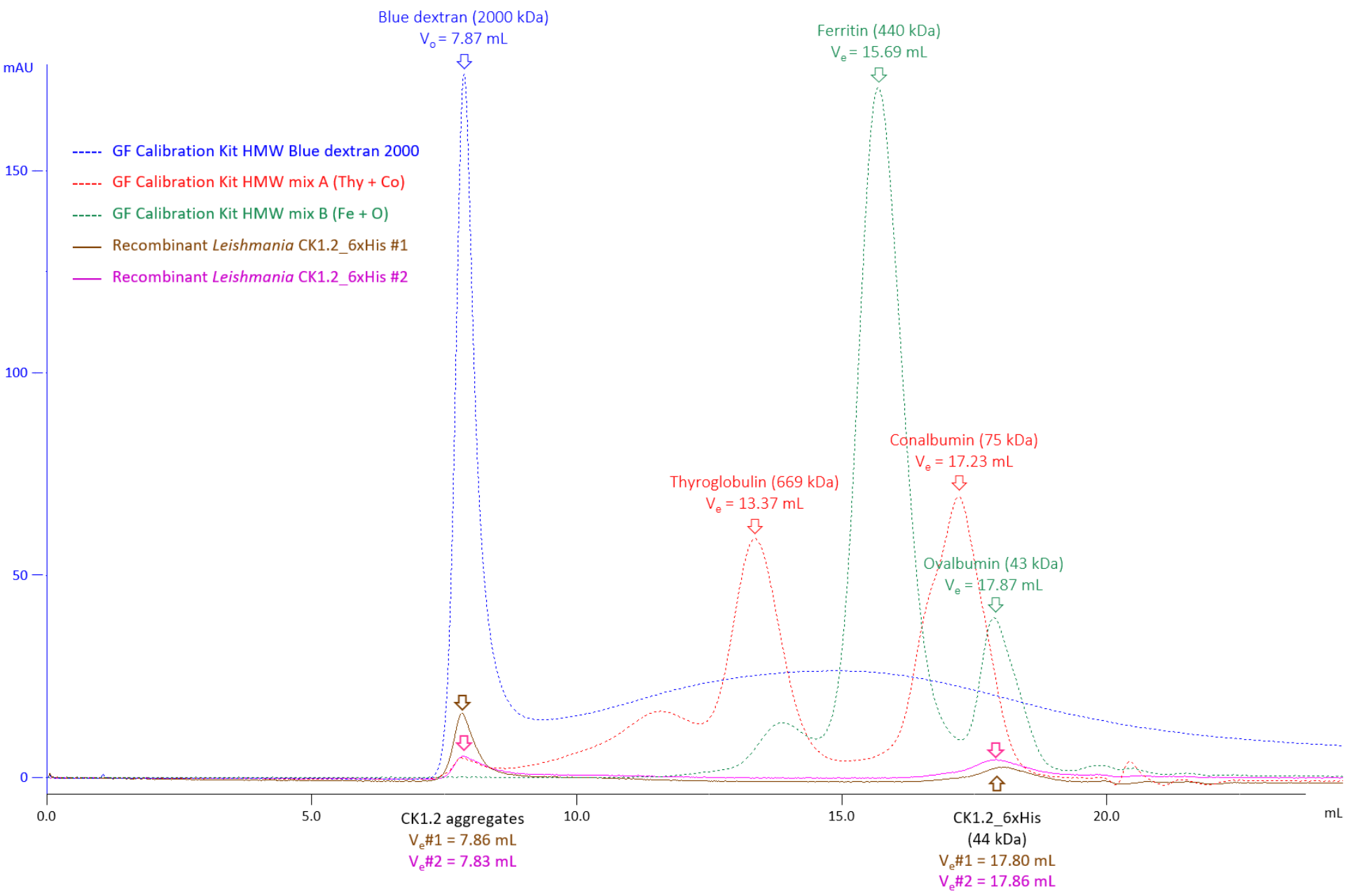

b

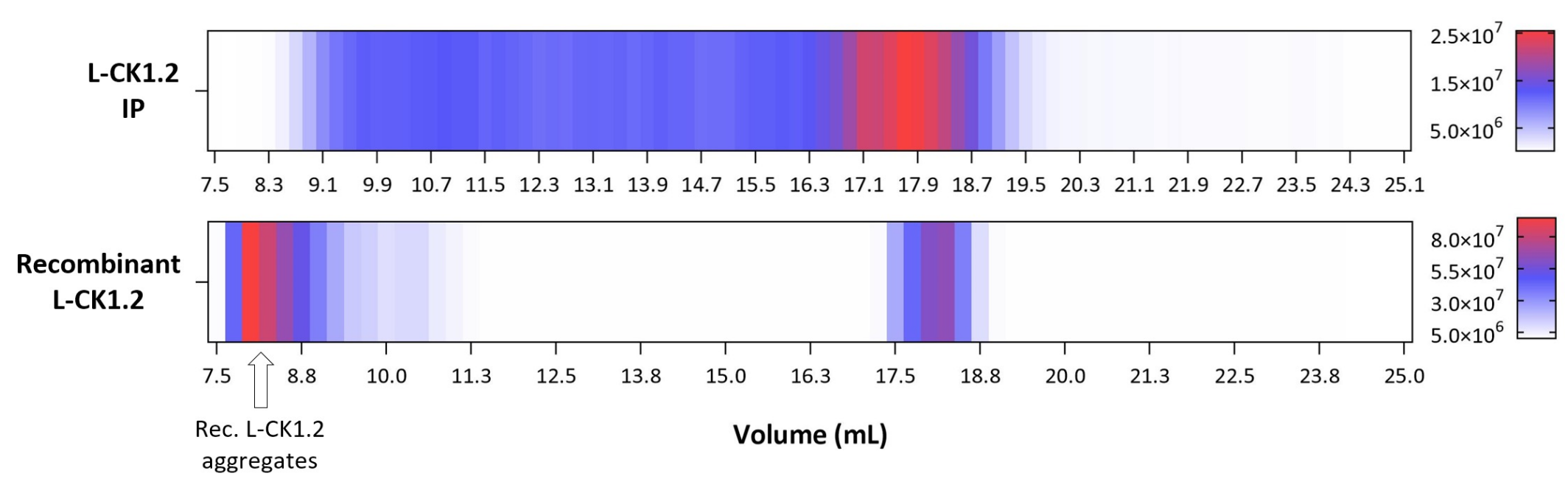

c

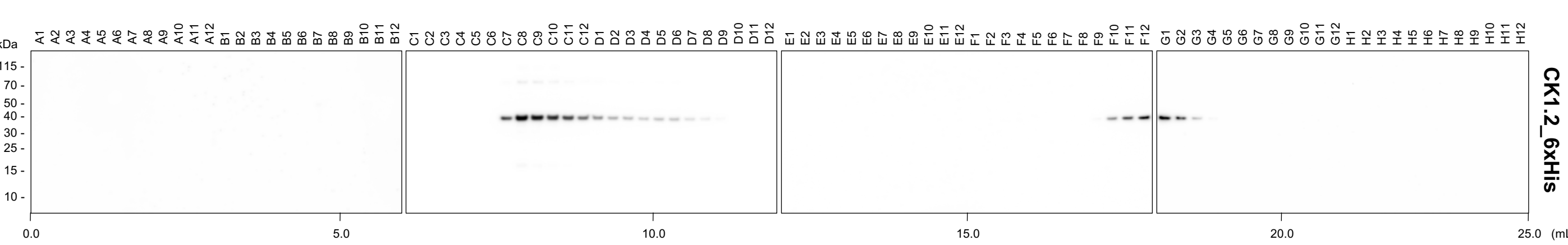

Figure S1

C

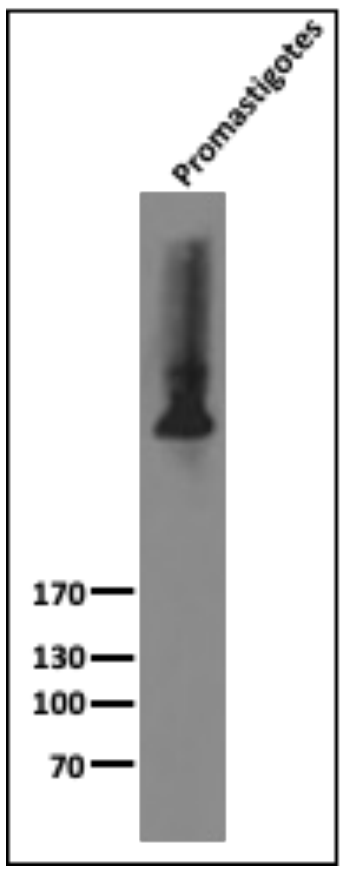

D

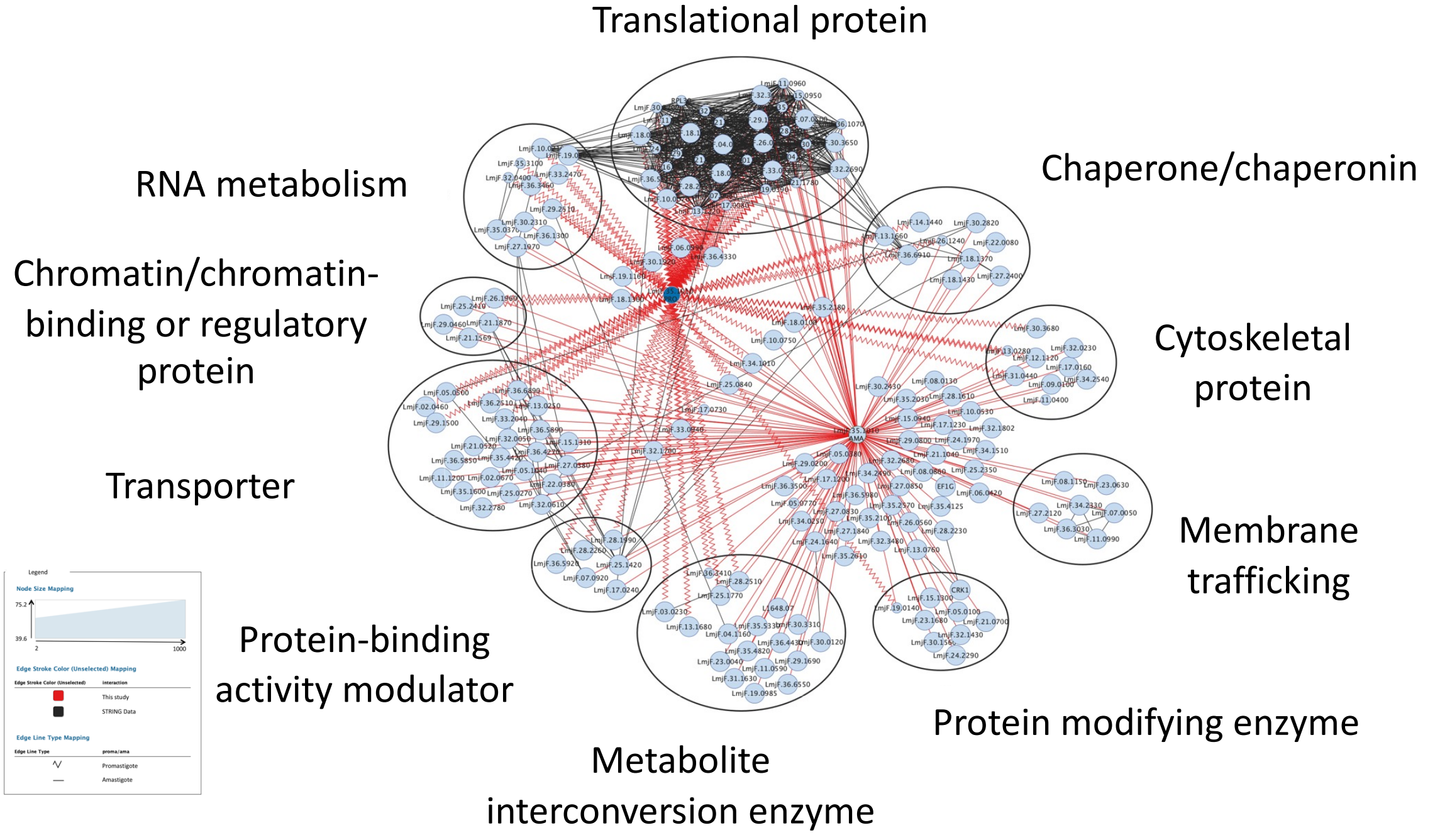

E

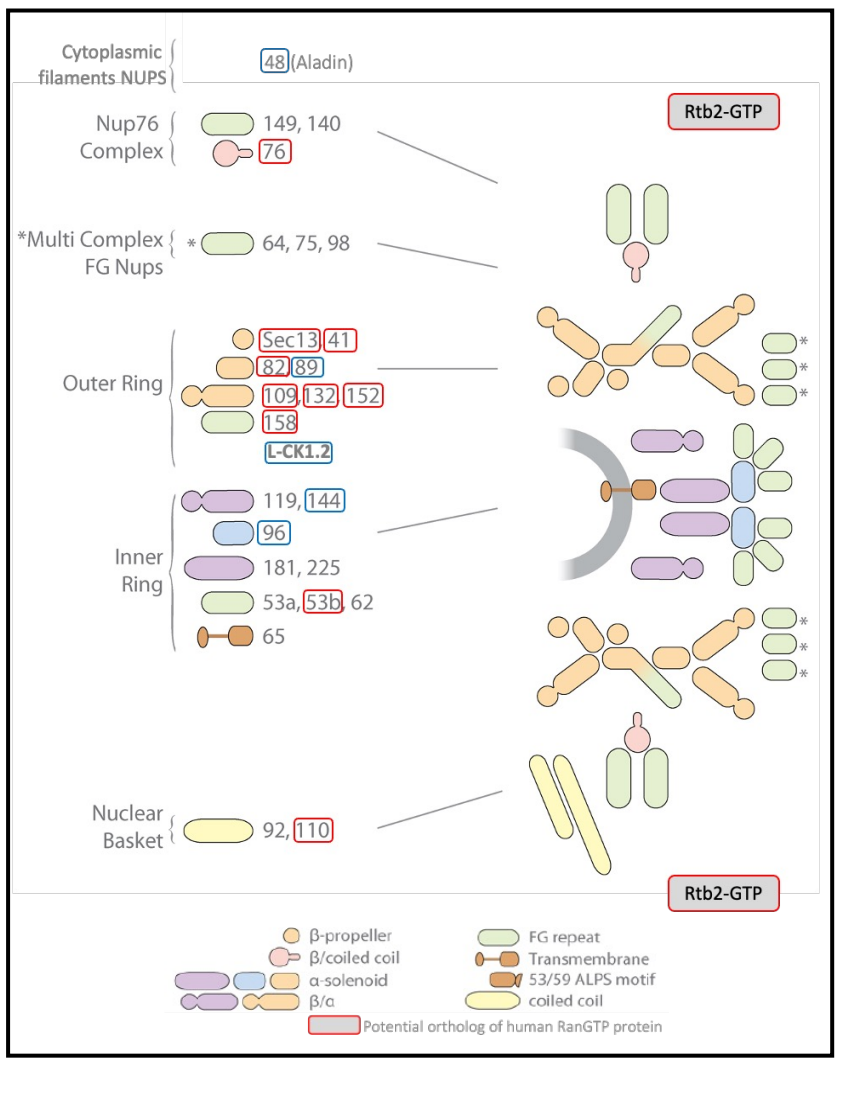

Supplement: Supplementary file 1 [file pone.0356818.s001.pdf]
