# Supplementary material for: Characterization of the dual functions of Leishmania CK1.2 in both the parasite and the macrophage
Source: PLoS One. 2026 Oct 5;21(10):e0356818. doi: 10.1371/journal.pone.0356818 (PMC13638044; doi:10.1371/journal.pone.0356818)
Supplement: Supplementary file 2 [file pone.0356818.s002.pdf]

Figure S2

A

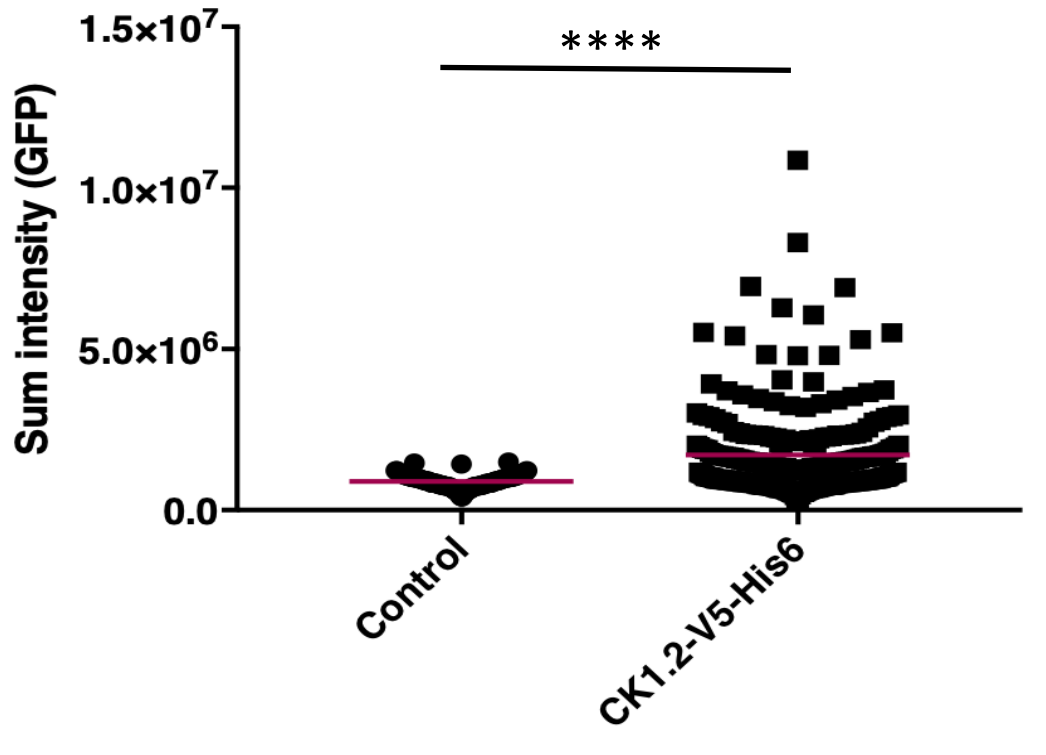

Sum intensity (GFP)
1.5×10^7
1.0×10^7
5.0×10^6
0.0
****
Control
CK1.2-V5-His6

B

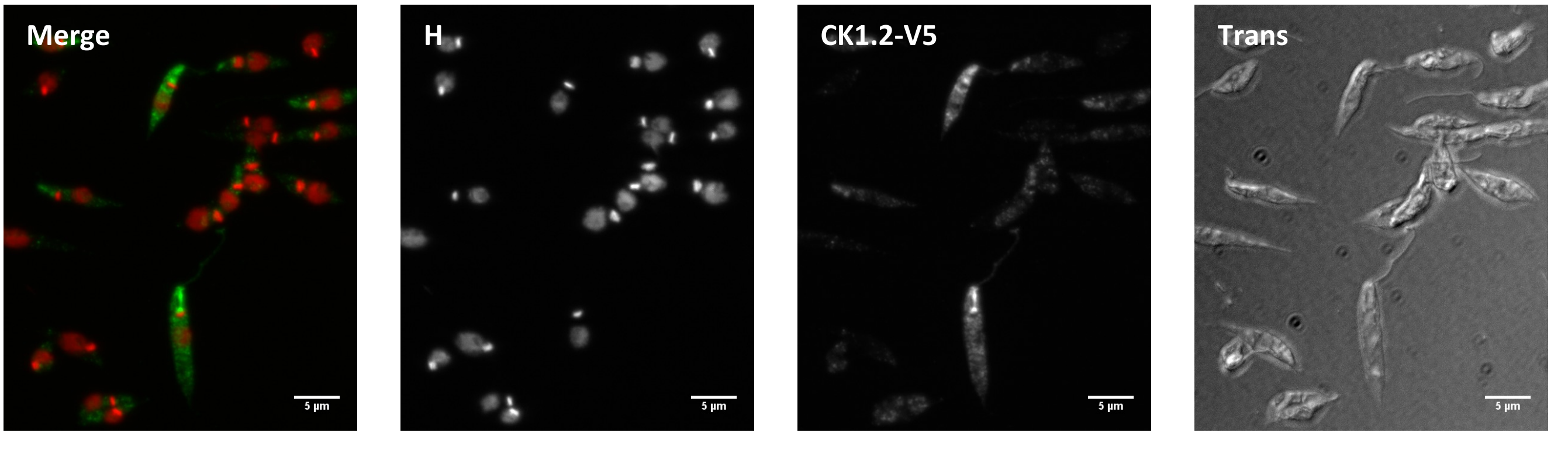

Merge
H
CK1.2-V5
Trans
5 µm
