# Supplementary material for: Characterization of the dual functions of Leishmania CK1.2 in both the parasite and the macrophage
Source: PLoS One. 2026 Oct 5;21(10):e0356818. doi: 10.1371/journal.pone.0356818 (PMC13638044; doi:10.1371/journal.pone.0356818)
Supplement: Supplementary file 3 [file pone.0356818.s003.pdf]

Figure S3

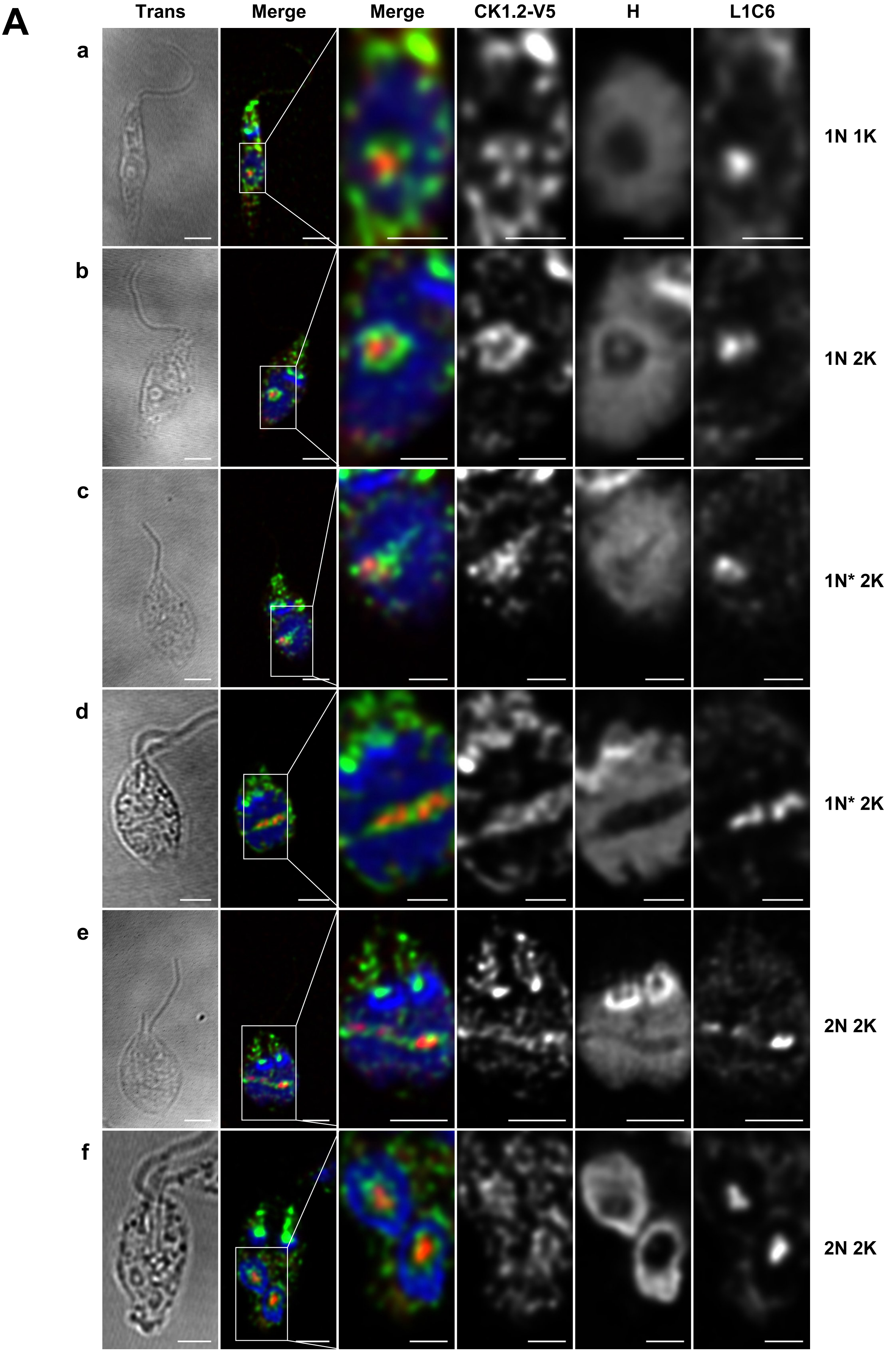
A
Trans
Merge
Merge
CK1.2-V5
H
L1C6
a
b
c
d
e
f
1N 1K
1N 2K
1N* 2K
1N* 2K
2N 2K
2N 2K

Figure S3

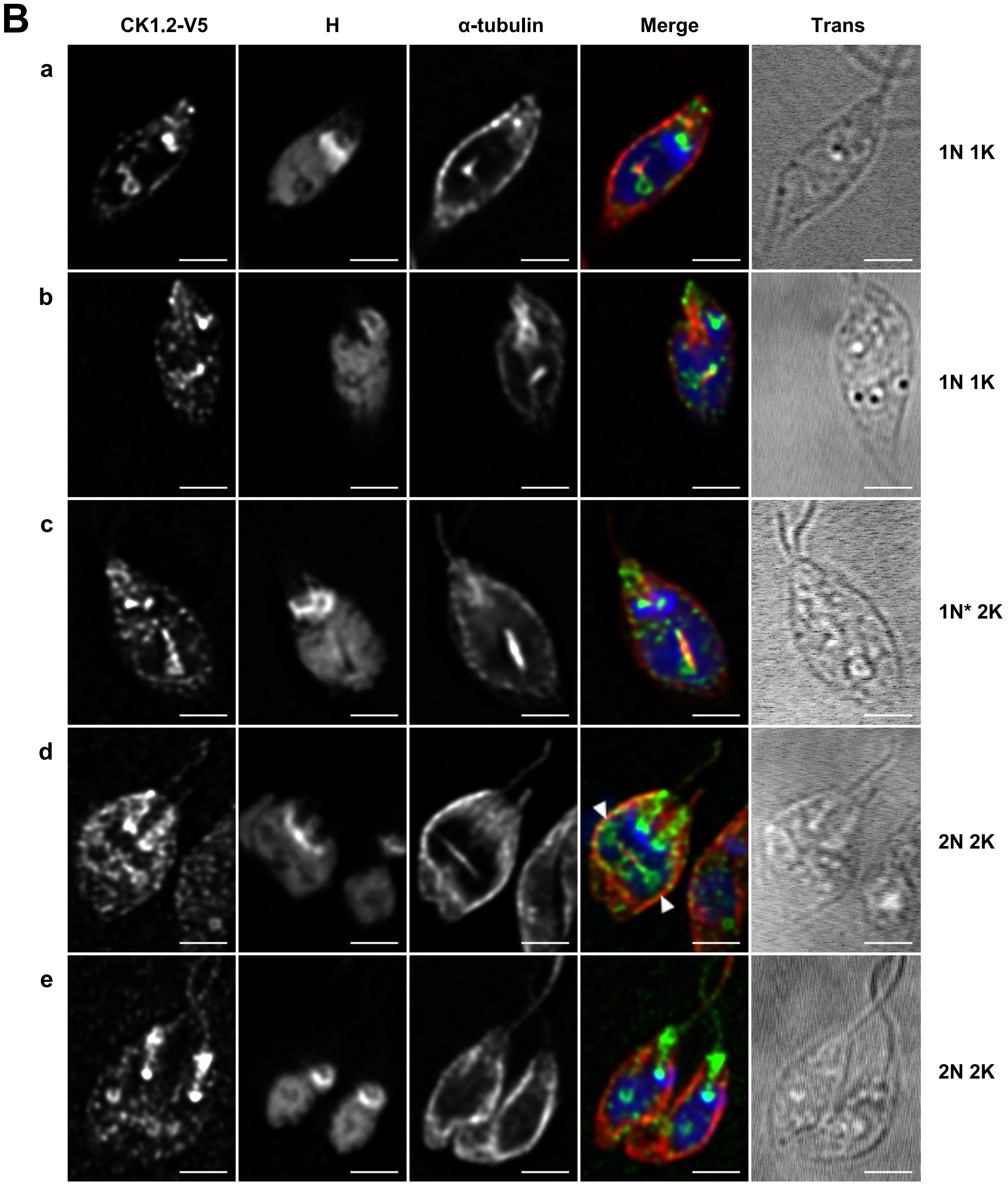
B
CK1.2-V5
H
α-tubulin
Merge
Trans
a
b
c
d
e
1N 1K
1N 1K
1N* 2K
2N 2K
2N 2K
