# Supplementary material for: Characterization of the dual functions of Leishmania CK1.2 in both the parasite and the macrophage
Source: PLoS One. 2026 Oct 5;21(10):e0356818. doi: 10.1371/journal.pone.0356818 (PMC13638044; doi:10.1371/journal.pone.0356818)
Supplement: Supplementary file 4 [file pone.0356818.s004.pdf]

Figure S4

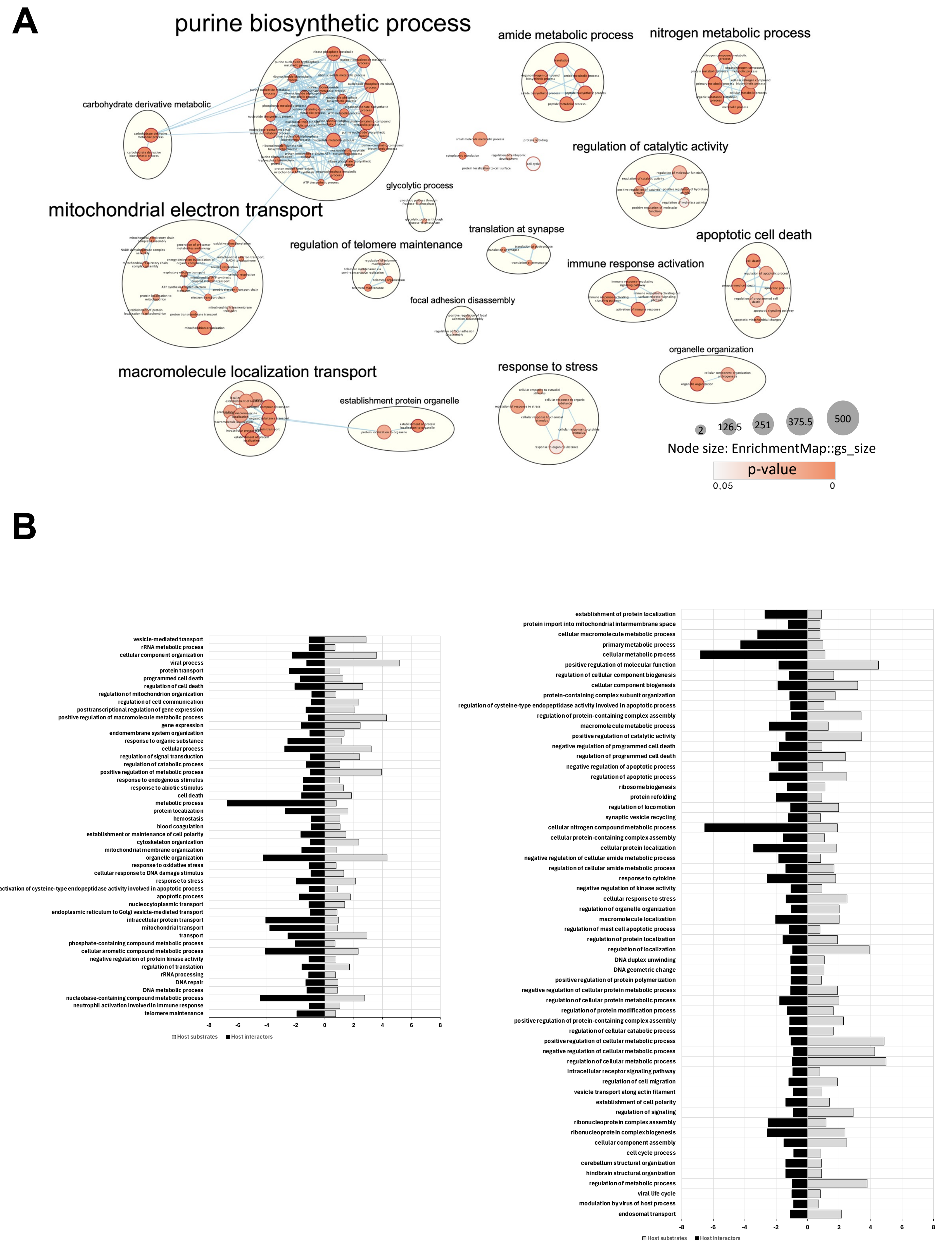
A
purine biosynthetic process
amide metabolic process
nitrogen metabolic process
carbohydrate derivative metabolic
regulation of catalytic activity
glycolytic process
mitochondrial electron transport
translation at synapse
regulation of telomere maintenance
apoptotic cell death
immune response activation
focal adhesion disassembly
organelle organization
macromolecule localization transport
response to stress
establishment protein organelle
2
126.5
251
375.5
500
Node size: EnrichmentMap::gs_size
p-value
0,05
0
B
vesicle-mediated transport
rRNA metabolic process
cellular component organization
viral process
protein transport
programmed cell death
regulation of cell death
regulation of mitochondrion organization
regulation of cell communication
posttranscriptional regulation of gene expression
positive regulation of macromolecule metabolic process
gene expression
endomembrane system organization
response to organic substance
cellular process
regulation of signal transduction
regulation of catabolic process
positive regulation of metabolic process
response to endogenous stimulus
response to abiotic stimulus
cell death
metabolic process
protein localization
hemostasis
blood coagulation
establishment or maintenance of cell polarity
cytoskeleton organization
mitochondrial membrane organization
organelle organization
response to oxidative stress
cellular response to DNA damage stimulus
response to stress
activation of cysteine-type endopeptidase activity involved in apoptotic process
apoptotic process
nucleocytoplasmic transport
endoplasmic reticulum to Golgi vesicle-mediated transport
intracellular protein transport
mitochondrial transport
transport
phosphate-containing compound metabolic process
cellular aromatic compound metabolic process
negative regulation of protein kinase activity
regulation of translation
rRNA processing
DNA repair
DNA metabolic process
nucleobase-containing compound metabolic process
neutrophil activation involved in immune response
telomere maintenance
-8
-6
-4
-2
0
2
4
6
8
Host substrates
Host interactors
establishment of protein localization
protein import into mitochondrial intermembrane space
cellular macromolecule metabolic process
primary metabolic process
cellular metabolic process
positive regulation of molecular function
regulation of cellular component biogenesis
cellular component biogenesis
protein-containing complex subunit organization
regulation of cysteine-type endopeptidase activity involved in apoptotic process
regulation of protein-containing complex assembly
macromolecule metabolic process
positive regulation of catalytic activity
negative regulation of programmed cell death
regulation of programmed cell death
negative regulation of apoptotic process
regulation of apoptotic process
ribosome biogenesis
protein refolding
regulation of locomotion
synaptic vesicle recycling
cellular nitrogen compound metabolic process
cellular protein-containing complex assembly
cellular protein localization
negative regulation of cellular amide metabolic process
regulation of cellular amide metabolic process
response to cytokine
negative regulation of kinase activity
cellular response to stress
regulation of organelle organization
macromolecule localization
regulation of mast cell apoptotic process
regulation of protein localization
regulation of localization
DNA duplex unwinding
DNA geometric change
positive regulation of protein polymerization
negative regulation of cellular protein metabolic process
regulation of cellular protein metabolic process
regulation of protein modification process
positive regulation of protein-containing complex assembly
regulation of cellular catabolic process
positive regulation of cellular metabolic process
negative regulation of cellular metabolic process
regulation of cellular metabolic process
intracellular receptor signaling pathway
regulation of cell migration
vesicle transport along actin filament
establishment of cell polarity
regulation of signaling
ribonucleoprotein complex assembly
ribonucleoprotein complex biogenesis
cellular component assembly
cell cycle process
cerebellum structural organization
hindbrain structural organization
regulation of metabolic process
viral life cycle
modulation by virus of host process
endosomal transport
-8
-6
-4
-2
0
2
4
6
8
Host substrates
Host interactors

Figure S4

B

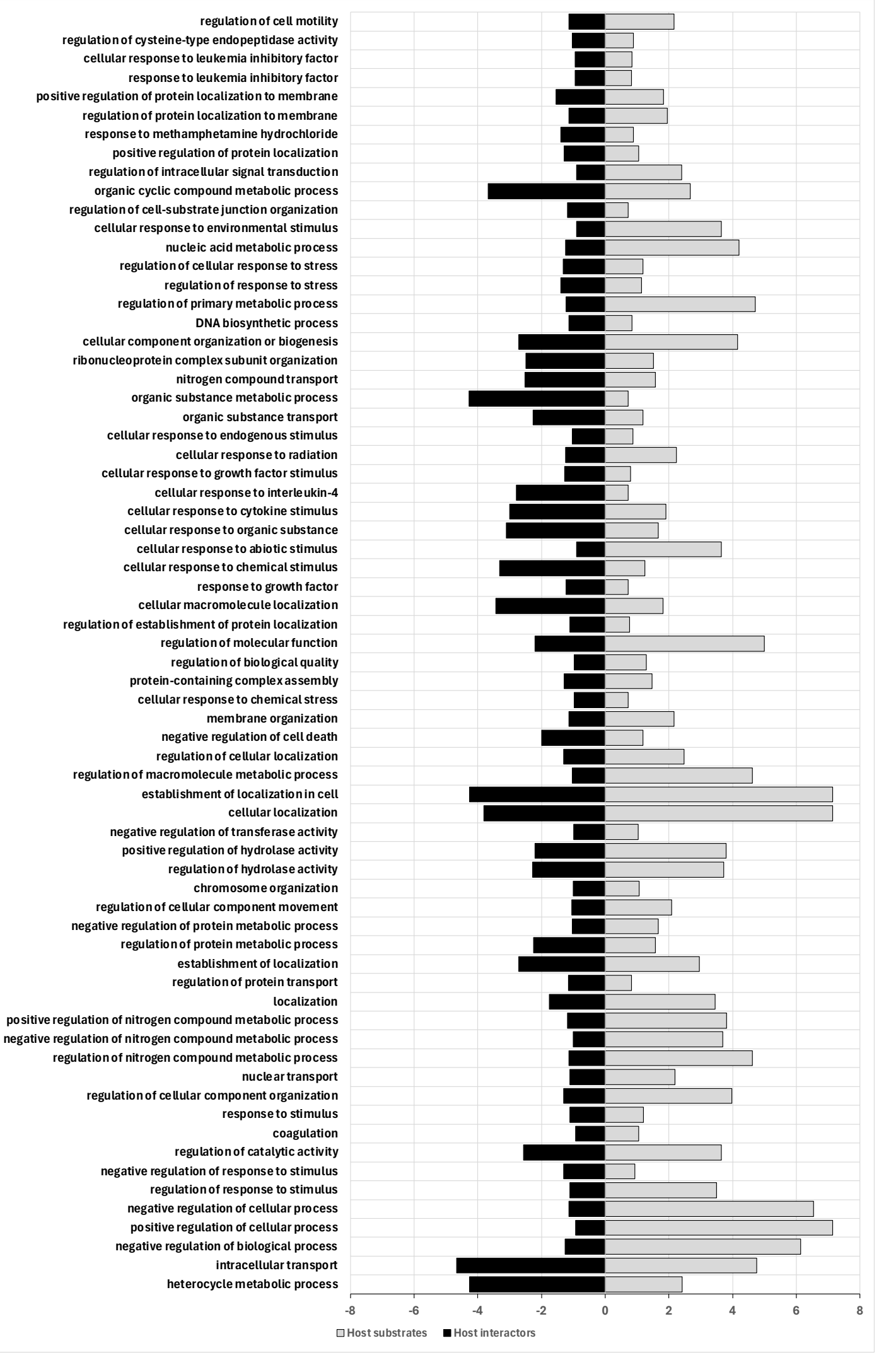
regulation of cell motility
regulation of cysteine-type endopeptidase activity
cellular response to leukemia inhibitory factor
response to leukemia inhibitory factor
positive regulation of protein localization to membrane
regulation of protein localization to membrane
response to methamphetamine hydrochloride
positive regulation of protein localization
regulation of intracellular signal transduction
organic cyclic compound metabolic process
regulation of cell-substrate junction organization
cellular response to environmental stimulus
nucleic acid metabolic process
regulation of cellular response to stress
regulation of response to stress
regulation of primary metabolic process
DNA biosynthetic process
cellular component organization or biogenesis
ribonucleoprotein complex subunit organization
nitrogen compound transport
organic substance metabolic process
organic substance transport
cellular response to endogenous stimulus
cellular response to radiation
cellular response to growth factor stimulus
cellular response to interleukin-4
cellular response to cytokine stimulus
cellular response to organic substance
cellular response to abiotic stimulus
cellular response to chemical stimulus
response to growth factor
cellular macromolecule localization
regulation of establishment of protein localization
regulation of molecular function
regulation of biological quality
protein-containing complex assembly
cellular response to chemical stress
membrane organization
negative regulation of cell death
regulation of cellular localization
regulation of macromolecule metabolic process
establishment of localization in cell
cellular localization
negative regulation of transferase activity
positive regulation of hydrolase activity
regulation of hydrolase activity
chromosome organization
regulation of cellular component movement
negative regulation of protein metabolic process
regulation of protein metabolic process
establishment of localization
regulation of protein transport
localization
positive regulation of nitrogen compound metabolic process
negative regulation of nitrogen compound metabolic process
regulation of nitrogen compound metabolic process
nuclear transport
regulation of cellular component organization
response to stimulus
coagulation
regulation of catalytic activity
negative regulation of response to stimulus
regulation of response to stimulus
negative regulation of cellular process
positive regulation of cellular process
negative regulation of biological process
intracellular transport
heterocycle metabolic process
-8
-6
-4
-2
0
2
4
6
8
Host substrates
Host interactors
