# Supplementary material for: Characterization of the dual functions of Leishmania CK1.2 in both the parasite and the macrophage
Source: PLoS One. 2026 Oct 5;21(10):e0356818. doi: 10.1371/journal.pone.0356818 (PMC13638044; doi:10.1371/journal.pone.0356818)
Supplement: Supplementary file 6 [file pone.0356818.s008.pdf]

Table S3: protein class

1st level	Gene number	
	PRO	AxAMA
protein modifying enzyme (PC00260)	2	10
transporter (PC00227)	5	11
membrane traffic protein (PC00150)	0	7
chaperone (PC00072)	6	7
DNA metabolism protein (PC00009)	0	1
protein-binding activity modulator (PC00095)	1	4
RNA metabolism protein (PC00171)	4	4
cytoskeletal protein (PC00085)	1	5
gene-specific transcriptional regulator (PC00264)	1	1
translational protein (PC00263)	33	17
metabolite interconversion enzyme (PC00262)	5	14
chromatin-binding, or -regulatory protein (PC00077)	1	4
