# Supplementary material for: Characterization of the dual functions of Leishmania CK1.2 in both the parasite and the macrophage
Source: PLoS One. 2026 Oct 5;21(10):e0356818. doi: 10.1371/journal.pone.0356818 (PMC13638044; doi:10.1371/journal.pone.0356818)
Supplement: Supplementary file 7 [file pone.0356818.s009.pdf]

# Table S4

| GO term I.D | Name | Promastigote | Amastigote |
|---|---|---|---|
| GO:0043604 | amide biosynthetic process | -19,36370396 | -2,0897205 |
| GO:0043603 | cellular amide metabolic process | -18,90042635 | -1,93957796 |
| GO:0043043 | peptide biosynthetic process | -19,59387427 | -2,11950817 |
| GO:0006518 | peptide metabolic process | -19,36370396 | -2,07733366 |
| GO:0019538 | protein metabolic process | -9,042518047 | -1,14377057 |
| GO:0044267 | cellular protein metabolic process | -9,460530479 | -1,34478212 |
| GO:1901566 | organonitrogen compound biosynthetic process | -15,91173771 | -0,96153052 |
| GO:1901564 | organonitrogen compound metabolic process | -8,806121901 | -0,81234035 |
| GO:0044271 | cellular nitrogen compound biosynthetic process | -15,11475796 | -0,97152353 |
| GO:0015833 | peptide transport | 0 | -3,03206351 |
| GO:0006457 | protein folding | 0 | -2,17762793 |
| GO:0042026 | protein refolding | 0 | -4,38858026 |
| GO:0010467 | gene expression | -13,62659037 | -0,95129354 |
| GO:0006412 | translation | -19,59387427 | -2,13709376 |
| GO:0006403 | RNA localization | -1,356609534 | -3,03206351 |
| GO:0051236 | establishment of RNA localization | -1,356609534 | -3,03206351 |
| GO:0050658 | RNA transport | -1,356609534 | -3,03206351 |
| GO:0051028 | mRNA transport | -1,380343105 | -3,03206351 |
| GO:0050657 | nucleic acid transport | -1,356609534 | -3,03206351 |
| GO:0015931 | nucleobase-containing compound transport | -0,76709067 | -1,92145543 |
| GO:0006913 | nucleocytoplasmic transport | 0 | -2,30522021 |
| GO:0051169 | nuclear transport | 0 | -2,30522021 |
| GO:0045184 | establishment of protein localization | 0 | -3,03206351 |
| GO:0008104 | protein localization | 0 | -3,03206351 |
| GO:0015031 | protein transport | 0 | -3,03206351 |
| GO:0033036 | macromolecule localization | 0 | -3,03206351 |
| GO:0051234 | establishment of localization | 0 | -2,10911542 |
| GO:0051649 | establishment of localization in cell | 0 | -2,10518879 |
| GO:0051641 | cellular localization | 0 | -1,93957796 |
| GO:0051179 | localization | 0 | -1,79907691 |
| GO:0006897 | endocytosis | 0 | -3,03206351 |
| GO:0072583 | clathrin-dependent endocytosis | 0 | -2,21599558 |
| GO:0016192 | vesicle-mediated transport | 0 | -2,0897205 |
| GO:0006898 | receptor-mediated endocytosis | 0 | -1,96140305 |
| GO:0006810 | transport | 0 | -2,11452444 |
| GO:0046907 | intracellular transport | 0 | -2,10518879 |
| GO:0071705 | nitrogen compound transport | 0 | -3,03206351 |
| GO:0071702 | organic substance transport | 0 | -2,60647099 |
| GO:0044249 | cellular biosynthetic process | -11,1836697 | 0 |
| GO:1901576 | organic substance biosynthetic process | -10,90494066 | 0 |
| GO:0009058 | biosynthetic process | -10,59364159 | 0 |
| GO:0009059 | macromolecule biosynthetic process | -14,406672 | -0,81234035 |
| GO:0034645 | cellular macromolecule biosynthetic process | -14,406672 | -0,81234035 |
| GO:0008152 | metabolic process | -4,688003775 | 0 |
| GO:0034641 | cellular nitrogen compound metabolic process | -10,3117029 | 0 |
| GO:0006807 | nitrogen compound metabolic process | -6,621445842 | 0 |
| GO:0044260 | cellular macromolecule metabolic process | -6,601021851 | 0 |
| GO:0043170 | macromolecule metabolic process | -5,955637792 | 0 |
| GO:0044238 | primary metabolic process | -5,803469345 | 0 |
| GO:0071704 | organic substance metabolic process | -5,310895053 | 0 |
| GO:0044237 | cellular metabolic process | -4,895035639 | 0 |
| GO:0008150 | biological process | -4,571641988 | -1,15925941 |
| GO:0009987 | cellular process | -3,939577983 | -1,07442949 |
