# Supplementary material for: Characterization of the dual functions of Leishmania CK1.2 in both the parasite and the macrophage
Source: PLoS One. 2026 Oct 5;21(10):e0356818. doi: 10.1371/journal.pone.0356818 (PMC13638044; doi:10.1371/journal.pone.0356818)
Supplement: Supplementary file 8 [file pone.0356818.s010.pdf]

Table S5

GO	Name	Count1	Count2	Rate1	Rate2	OR	CI_ lower	CI_ upper	p_value	FDR_BH	Direction
GO:0008152	metabolic process	40	0	0,47	0,00	256,94	15,49	4260,55	3,11369E-21	2,74004E-19	▲ Promastigote
GO:0044238	primary metabolic process	38	0	0,44	0,00	234,18	14,12	3884,19	5,22122E-20	1,53156E-18	▲ Promastigote
GO:0071704	organic substance metabolic process	38	0	0,44	0,00	234,18	14,12	3884,19	5,22122E-20	1,53156E-18	▲ Promastigote
GO:0006807	nitrogen compound metabolic process	37	0	0,43	0,00	223,48	13,47	3707,57	2,08849E-19	4,59467E-18	▲ Promastigote
GO:0044237	cellular metabolic process	36	0	0,42	0,00	213,22	12,85	3538,04	8,22864E-19	1,44824E-17	▲ Promastigote
GO:0034641	cellular nitrogen compound metabolic process	33	0	0,38	0,00	184,72	11,12	3067,97	4,61346E-17	5,79978E-16	▲ Promastigote
GO:0043170	macromolecule metabolic process	33	0	0,38	0,00	184,72	11,12	3067,97	4,61346E-17	5,79978E-16	▲ Promastigote
GO:0009058	biosynthetic process	29	0	0,34	0,00	151,35	9,10	2518,43	8,18241E-15	6,54593E-14	▲ Promastigote
GO:0044260	cellular macromolecule metabolic process	29	0	0,34	0,00	151,35	9,10	2518,43	8,18241E-15	6,54593E-14	▲ Promastigote
GO:0044249	cellular biosynthetic process	29	0	0,34	0,00	151,35	9,10	2518,43	8,18241E-15	6,54593E-14	▲ Promastigote
GO:1901576	organic substance biosynthetic process	29	0	0,34	0,00	151,35	9,10	2518,43	8,18241E-15	6,54593E-14	▲ Promastigote
GO:1901566	organonitrogen compound biosynthetic process	29	13	0,34	0,09	5,11	2,50	10,44	5,00404E-06	3,66963E-05	▲ Promastigote
GO:0006518	peptide metabolic process	27	12	0,31	0,08	5,01	2,40	10,45	8,20889E-06	3,80201E-05	▲ Promastigote
GO:0043043	peptide biosynthetic process	27	12	0,31	0,08	5,01	2,40	10,45	8,20889E-06	3,80201E-05	▲ Promastigote
GO:0034645	cellular macromolecule biosynthetic process	27	12	0,31	0,08	5,01	2,40	10,45	8,20889E-06	3,80201E-05	▲ Promastigote
GO:0006412	translation	27	12	0,31	0,08	5,01	2,40	10,45	8,20889E-06	3,80201E-05	▲ Promastigote
GO:0043603	cellular amide metabolic process	27	12	0,31	0,08	5,01	2,40	10,45	8,20889E-06	3,80201E-05	▲ Promastigote
GO:0009059	macromolecule biosynthetic process	27	12	0,31	0,08	5,01	2,40	10,45	8,20889E-06	3,80201E-05	▲ Promastigote
GO:0043604	amide biosynthetic process	27	12	0,31	0,08	5,01	2,40	10,45	8,20889E-06	3,80201E-05	▲ Promastigote
GO:0044271	cellular nitrogen compound biosynthetic process	28	13	0,33	0,09	4,85	2,37	9,94	1,02696E-05	4,51862E-05	▲ Promastigote
GO:0010467	gene expression	30	16	0,35	0,11	4,30	2,19	8,45	1,51649E-05	6,35483E-05	▲ Promastigote
GO:0051179	localization	0	19	0,00	0,13	0,04	0,00	0,64	0,000114562	0,000420059	▼ Amastigote
GO:0006810	transport	0	19	0,00	0,13	0,04	0,00	0,64	0,000114562	0,000420059	▼ Amastigote
GO:0051234	establishment of localization	0	19	0,00	0,13	0,04	0,00	0,64	0,000114562	0,000420059	▼ Amastigote
GO:1901564	organonitrogen compound metabolic process	33	25	0,38	0,17	3,01	1,64	5,52	0,000481194	0,001693801	▲ Promastigote
GO:0019538	protein metabolic process	30	23	0,35	0,16	2,86	1,53	5,33	0,001098777	0,003718936	▲ Promastigote
GO:0044267	cellular protein metabolic process	29	22	0,34	0,15	2,86	1,52	5,38	0,001613193	0,005257814	▲ Promastigote
GO:0071705	nitrogen compound transport	0	13	0,00	0,09	0,06	0,00	0,98	0,002462752	0,007224073	▼ Amastigote
GO:0071702	organic substance transport	0	13	0,00	0,09	0,06	0,00	0,98	0,002462752	0,007224073	▼ Amastigote
GO:0033036	macromolecule localization	0	13	0,00	0,09	0,06	0,00	0,98	0,002462752	0,007224073	▼ Amastigote
GO:0015031	protein transport	0	12	0,00	0,08	0,06	0,00	1,07	0,004386426	0,011697137	▼ Amastigote
GO:0008104	protein localization	0	12	0,00	0,08	0,06	0,00	1,07	0,004386426	0,011697137	▼ Amastigote
GO:0045184	establishment of protein localization	0	12	0,00	0,08	0,06	0,00	1,07	0,004386426	0,011697137	▼ Amastigote
GO:0051641	cellular localization	0	10	0,00	0,07	0,08	0,00	1,31	0,014881765	0,034463035	▼ Amastigote
GO:0051649	establishment of localization in cell	0	10	0,00	0,07	0,08	0,00	1,31	0,014881765	0,034463035	▼ Amastigote
GO:0046907	intracellular transport	0	10	0,00	0,07	0,08	0,00	1,31	0,014881765	0,034463035	▼ Amastigote
GO:0015833	peptide transport	0	8	0,00	0,05	0,09	0,01	1,66	0,027767769	0,061089093	▼ Amastigote
GO:0016192	vesicle-mediated transport	0	8	0,00	0,05	0,09	0,01	1,66	0,027767769	0,061089093	▼ Amastigote
GO:0008150	biological process	51	66	0,59	0,45	1,78	1,04	3,04	0,041669084	0,089436083	▲ Promastigote
GO:0009987	cellular process	44	56	0,51	0,38	1,70	0,99	2,90	0,056106417	0,112212834	▲ Promastigote
GO:0006457	protein folding	0	6	0,00	0,04	0,13	0,01	2,26	0,087522216	0,160457396	▼ Amastigote
GO:0042026	protein refolding	0	4	0,00	0,03	0,18	0,01	3,47	0,299494712	0,506837205	▼ Amastigote
GO:0006913	nucleocytoplasmic transport	0	4	0,00	0,03	0,18	0,01	3,47	0,299494712	0,506837205	▼ Amastigote
GO:0051169	nuclear transport	0	4	0,00	0,03	0,18	0,01	3,47	0,299494712	0,506837205	▼ Amastigote
GO:0006897	endocytosis	0	4	0,00	0,03	0,18	0,01	3,47	0,299494712	0,506837205	▼ Amastigote
GO:0006898	receptor-mediated endocytosis	0	2	0,00	0,01	0,34	0,02	7,09	0,532262839	0,720601997	▼ Amastigote
GO:0072583	clathrin-dependent endocytosis	0	2	0,00	0,01	0,34	0,02	7,09	0,532262839	0,720601997	▼ Amastigote
GO:0015931	nucleobase-containing compound transport	2	4	0,02	0,03	0,94	0,20	4,53	1	1	▼ Amastigote
GO:0006403	RNA localization	2	4	0,02	0,03	0,94	0,20	4,53	1	1	▼ Amastigote
GO:0050658	RNA transport	2	4	0,02	0,03	0,94	0,20	4,53	1	1	▼ Amastigote
GO:0051028	mRNA transport	2	4	0,02	0,03	0,94	0,20	4,53	1	1	▼ Amastigote
GO:0051236	establishment of RNA localization	2	4	0,02	0,03	0,94	0,20	4,53	1	1	▼ Amastigote
GO:0050657	nucleic acid transport	2	4	0,02	0,03	0,94	0,20	4,53	1	1	▼ Amastigote
