# Supplementary material for: Characterization of the dual functions of Leishmania CK1.2 in both the parasite and the macrophage
Source: PLoS One. 2026 Oct 5;21(10):e0356818. doi: 10.1371/journal.pone.0356818 (PMC13638044; doi:10.1371/journal.pone.0356818)
Supplement: Supplementary file 9 [file pone.0356818.s011.pdf]

Table S6

Localisation	Corresponding genes
**Protein/vesicular trafficking**	60S ribosomal protein L21 (LdBPK_343440.1); Nodulin-like/ Pyruvate transporter (LdBPK_291600.1); LdBPK_071070.1; Organic solute transporter Ostalpha (LdBPK_111190.1); C2 domain containing protein (LdBPK_130650.1); LdBPK_171330.1; heat shock protein DNAJ (LdBPK_272350.1); cyclin dependent kinase-binding protein (LdBPK_282390.1); Gem-associated protein 2 (LdBPK_361350.1).
**Nucleolus**	40S ribosomal protein S7 (LdBPK_010430.1), S5 (LdBPK_110960.1), S4 (LdBPK_131120.1), S6 (LmjF.21.1780), S11 (LdBPK_211790.1), S13 (LdBPK_333300.1), S9 (LdBPK_361310.1) ; 60S ribosomal protein L11 (LdBPK_220004.1), L21 (LdBPK_343440.1), L7 (LdBPK_180230.1/LdBPK_260160.1), L10a (LdBPK_363950.1), L34 (LdBPK_363930.1), L26 (LdBPK_242140.1), L1a (LdBPK_291160.1), L13 (LdBPK_292570.1,LdBPK_292580.1), L18a (LdBPK_350600.1), L6 (LdBPK_330770.1), L30 (LdBPK_350240.1), L7a (LdBPK_070550.1), L27 (LdBPK_322830.1); nucleolar protein 56 (LdBPK_100210.1); alpha tubulin (LdBPK_130330.1); fibrillarin (LdBPK_190090.1,LdBPK_363220.1); LdBPK_252520.1; T-complex protein 1, theta subunit (LdBPK_367240.1); histone deacetylase (LdBPK_081300.1); Nucleoporin NUP41 (LdBPK_210580.1); heat shock protein DNAJ (LdBPK_220009.1); class I transcription factor A, subunit 1 (LdBPK_270681.1).
**Nucleus / kinetoplast / antipodal sites**	nucleolar protein 56 (LdBPK_100210.1); LdBPK_180100.1; fibrillarin (LdBPK_190090.1, LdBPK_363220.1); 40S ribosomal protein S3A(LdBPK_350400.1), S9 (LdBPK_361310.1); 60S ribosomal protein L26 (LdBPK_242140.1); heat shock protein 70, putative,heat-shock protein hsp70, putative,heat shock protein 70-related protein,luminal binding protein 1 (LdBPK_261220.1,LdBPK_281310.1,LdBPK_283000.1,LdBPK_283040.1); Nodulin-like, putative / Pyruvate transporter (LdBPK_291600.1); LdBPK_341080.1; casein kinase I (LdBPK_351030.1); kinetoplast DNA-associated protein (LdBPK_366180.1); ATP synthase F1, alpha subunit (LdBPK_050510.1); Microtubule associated protein (MAP65/ASE1 family, LdBPK_151000.1); histone deacetylase (LdBPK_081300.1); Nucleoporin NUP41 (LdBPK_210580.1); heat shock protein DNAJ (LdBPK_220009.1); class I transcription factor A, subunit 1 (LdBPK_270700.1); Small nuclear ribonucleoprotein-associated protein B (snRNP-B, LdBPK_271890.1); MSP domain containing protein (LdBPK_272040.1); heat shock protein DNAJ (LdBPK_272350.1); SNF2 family N-terminal domain/Helicase conserved C-terminal domain containing protein (LdBPK_290210.1); dynein light chain (LdBPK_320240.1); nucleoside diphosphate kinase b (LdBPK_323110.1); LdBPK_363660.1; Mitoribosomal LSU assembly factor 24 (LdBPK_302320.1); mitochondrial phosphate transporter (LdBPK_354490.1); mitochondrial carrier protein (LdBPK_020640.1).
**Nuclear pore**	Nucleoporin NUP48 (LdBPK_130250.1); NUP89 (LdBPK_332160.1); NUP96 (LdBPK_362640.1); NUP144 (NUP155-like, LdBPK_367220.1); LdBPK_091010.1; LdBPK_100580.1; NUP41 (LdBPK_210580.1); NUP132 (LdBPK_220260.1); NUP76 (LdBPK_242050.1); NUP110 (LdBPK_250270.1); GTP-binding nuclear protein rtb2 (LdBPK_251460.1); NUP158 (LdBPK_270390.1); NUP53b (LdBPK_290830.1); NUP109 (LdBPK_322920.1); NUP82 (LdBPK_351600.1); LdBPK_281740.1; protein transport protein Sec13 (LdBPK_320050.1); NUP152 (LdBPK_364480.1).
**Flagella connector/hook complex/FAZ/micro tubule quartet/flagellar pocket collar**	LdBPK_061020.1; LdBPK_181340.1; LdBPK_282120.1; FAZ 2 (LdBPK_120720.1); Associated kinase of Tb14-3-3 (LdBPK_190130.1); predicted C2 domain protein (LdBPK_364540.1); phosphoprotein phosphatase (KPP1, LdBPK_050100.1); Acyl-coenzyme A thioesterase (LdBPK_291820.1); dynein light chain, flagellar outer arm (LdBPK_320240.1); dual specificity protein phosphatase (LdBPK_210760.1); LdBPK_322820.1; Coiled-coil and C2 domain-containing protein (LdBPK_342370.1); MORN repeat-containing protein 1 (LdBPK_303360.1); LdBPK_352610.1; SUMO-interacting motif-containing protein (LdBPK_321490.1); bilbo1 (LdBPK_090120.1).
**Cortical cytoskeleton/Cell tip/membrane/pellicular membrane**	LdBPK_061020.1; alpha tubulin (LdBPK_130330.1); cytoskeleton associated protein (LdBPK_141540.1); LdBPK_191150.1; LdBPK_303740.1; cytoskeleton-associated protein CAP5.5 (LdBPK_310450.1); long chain fatty Acyl CoA synthetase (LdBPK_030220.1); FAZ 2 (LdBPK_120720.1); LdBPK_252520.1; casein kinase I (CK1.2, LdBPK_351030.1); microtubule-associated protein (LdBPK_050380.1) ; Dual specificity phosphatase, catalytic domain containing protein (LdBPK_271740.1); kharon1 (LdBPK_366110.1); dynein light chain, flagellar outer arm (LdBPK_320240.1); LdBPK_352610.1.
**Glycosome/acidocalcisome/ER/Golgi/lipid droplet**	glycosomal membrane protein (LdBPK_282430.1); ATP-dependent 6-phosphofructokinase (LdBPK_292620.1); long chain fatty Acyl CoA synthetase (LdBPK_030220.1); 3-oxo-5-alpha-steroid 4-dehydrogenase (LdBPK_251850.1); heat shock protein 70 (LdBPK_261220.1, LdBPK_281310.1, LdBPK_283000.1, LdBPK_283040.1); Nodulin-like, putative / Pyruvate transporter (LdBPK_291600.1); fructose-1,6-bisphosphatase (LdBPK_041170.1); alkyldihydroxyacetonephosphate synthase (LdBPK_300120.1); isopentenyl-diphosphate delta-isomerase (LdBPK_355300.1); MGT1 magnesium transporter (LdBPK_151330.1); protein transport protein SEC13 (LdBPK_320050.1); Histidine phosphatase superfamily (LdBPK_354880.1).
**Mitochondrion**	Mitochondrial outer membrane protein porin (LdBPK_020430.1); ATP synthase F1, alpha subunit (LdBPK_050510.1); LdBPK_061020.1; POMP11 / Present in the outer mitochondrial membrane proteome 11 (LdBPK_332600.1); mitochondrial carrier protein (LdBPK_020640.1); stomatin-like protein (LdBPK_051040.1); cell division protein kinase 2 (LdBPK_211320.1); Mitoribosomal LSU assembly factor 24 (LdBPK_302320.1); mitochondrial phosphate transporter (LdBPK_354490.1).
**Spindle**	Nucleus and spindle associated protein 2 (MAP65/ASE1 family, LdBPK_151000.1).

Table S6

Localisation	Corresponding genes
**Basal body, TAC and TF**	FAZ 2 (LdBPK_120720.1); Associated kinase of Tb14-3-3 (LdBPK_190130.1); casein kinase I (LdBPK_351030.1); microtubule-associated protein (LdBPK_050380.1); Regulator of chromosome condensation (RCC1) repeat (LdBPK_170290.1); dual specificity protein phosphatase (LdBPK_210760.1); Nucleoporin NUP41 (LdBPK_210580.1); peroxidoxin (LdBPK_230050.1); dynein light chain, flagellar outer arm (LdBPK_320240.1); LdBPK_323680.1; Tripartite Attachment Complex Protein 60 (LdBPK_260530.1); heat shock protein DNAJ (LdBPK_220009.1).
**Flagellar pocket**	long chain fatty Acyl CoA synthetase (LdBPK_030220.1); LdBPK_252520.1; Exocyst complex component Sec3 (LdBPK_081060.1); Exocyst complex component EXO99 (LdBPK_211910.1); exocyst complex component Sec10 (LdBPK_230800.1); MSP (Major sperm protein) domain containing protein (LdBPK_272040.1); LdBPK_323680.1; ; alpha-adaptin-like protein (LdBPK_070060.1); adaptin-related protein-like protein (LdBPK_110990.1); clathrin coat assembly protein AP17 (LdBPK_342100.1); clathrin coat assembly protein-like protein (LdBPK_363180.1).
**Flagellum/Flagellar tip/paraflagellar rod**	LdBPK_180100.1; Associated kinase of Tb14-3-3 (LdBPK_190130.1); 60S ribosomal protein L26 (LdBPK_242140.1); 40S ribosomal protein S9 (LdBPK_361310.1); Nucleoporin NUP96 (LdBPK_362640.1); 40S ribosomal protein SA (LdBPK_365353.1); kinetoplast DNA-associated protein (LdBPK_366180.1); heat shock protein 70 (LdBPK_261220.1, LdBPK_281310.1, LdBPK_283000.1, LdBPK_283040.1); alpha tubulin (LdBPK_130330.1); Nucleoporin NUP41 (LdBPK_210580.1); peroxidoxin (LdBPK_230050.1); MSP (Major sperm protein) domain containing protein (LdBPK_272040.1); heat shock protein DNAJ (LdBPK_272350.1); dynein light chain, flagellar outer arm (LdBPK_320240.1); nucleoside diphosphate kinase b (LdBPK_323110.1); Chaperonin HSP60 (LdBPK_302830.1,LdBPK_321940.1,LdBPK_362130.1,LdBPK_362140.1); Inositol hexakisphosphate (LdBPK_231720.1).
**Cytoplasm**	40S ribosomal protein S3 (LdBPK_330960.1), S4 (LdBPK_131120.1), S5 (LdBPK_110960.1), S6 (LmjF.21.1780), S7 (LdBPK_010430.1), S9 (LdBPK_361310.1), S11 (LdBPK_211790.1), S13 (LdBPK_333300.1), S17 (LdBPK_282750.1), S20 (LdBPK_281100.1); SA (LdBPK_365353.1); 60S ribosomal protein L1a (LdBPK_291160.1), L6 (LdBPK_330770.1), L7 (LdBPK_260160.1), L7a (LdBPK_070550.1), L10 (LdBPK_040750.1), L10a (LdBPK_363950.1); L11 (LdBPK_220004.1), L18a (LdBPK_350600.1), L21 (LdBPK_343440.1), L26 (LdBPK_242140.1), L24 (LdBPK_361130.1); L27 (LdBPK_322830.1); L28 (LdBPK_111110.1), L30 (LdBPK_350240.1), L34 (LdBPK_363930.1); alpha tubulin (LdBPK_130330.1); chaperonin TCP20 (LdBPK_131400.1); pyrroline-5-carboxylate reductase (LdBPK_131420.1); elongation factor 1-alpha (LdBPK_170170.1); LdBPK_180100.1; Associated kinase of Tb14-3-3 (LdBPK_190130.1); 3-oxo-5-alpha-steroid 4-dehydrogenase (LdBPK_251850.1); heat shock protein 70 (LdBPK_261220.1,LdBPK_281310.1,LdBPK_283000.1,LdBPK_283040.1); LdBPK_261960.1; acyl-CoA dehydrogenase (LdBPK_282700.1); Nodulin-like/ Pyruvate transporter (LdBPK_291600.1); ATP-dependent RNA helicase HEL67 (LdBPK_320410.1); casein kinase I (LdBPK_351030.1); Nucleoporin NUP96 (LdBPK_362640.1); C2 domain protein (LdBPK_130650.1, LdBPK_364540.1); kinetoplast DNA-associated protein (LdBPK_366180.1); LdBPK_071070.1; Microtubule associated protein (MAP65/ASE1 family, LdBPK_151000.1); ubiquitin hydrolase (LdBPK_151320.1); MGT1 magnesium transporter (LdBPK_151330.1); Regulator of chromosome condensation (RCC1) repeat (LdBPK_170290.1); LdBPK_171330.1; heat shock protein 110 (LdBPK_181350.1); kinesin (LdBPK_211280.1); dual specificity protein phosphatase (LdBPK_210760.1); Nucleoporin NUP41 (LdBPK_210580.1); heat shock protein DNAJ (LdBPK_220009.1); peroxidoxin (LdBPK_230050.1); exocyst complex component Sec10 (LdBPK_230800.1); Male sterility protein (LdBPK_241710.1); Nucleoporin NUP76 (LdBPK_242050.1); Nucleoporin NUP110 (LdBPK_250270.1); GTP-binding nuclear protein rtb2 (LdBPK_251460.1); LdBPK_252520.1; Dual specificity phosphatase (LdBPK_271740.1); MSP (Major sperm protein) domain containing protein (LdBPK_272040.1); heat shock protein DNAJ (LdBPK_272350.1); LdBPK_281740.1; cyclin dependent kinase-binding protein (LdBPK_282390.1); Acyl-coenzyme A thioesterase (LdBPK_291820.1); alkyldihydroxyacetonephosphate synthase (LdBPK_300120.1); Mitoribosomal LSU assembly factor 24 (LdBPK_302320.1); dynein light chain, flagellar outer arm (LdBPK_320240.1); LdBPK_320640.1; LdBPK_322820.1; nucleoside diphosphate kinase b (LdBPK_323110.1); LdBPK_323680.1; ATP-dependent DEAD-box RNA helicase (LdBPK_350370.1); LdBPK_352610.1; Gem-associated protein 2 (LdBPK_361350.1); Chaperonin HSP60 (LdBPK_302830.1, LdBPK_321940.1, LdBPK_362130.1, LdBPK_362140.1); flagellum targeting protein kharon1 (LdBPK_366110.1).
**Unknown localisation**	LdBPK_100800.1; GRAM domain containing protein (LdBPK_170750.1); 40S ribosomal protein S23 (LdBPK_211300.1), S14 (LdBPK_303650.1), S26 (LdBPK_280570.1); LdBPK_250870.1; LdBPK_301900.1; LdBPK_321780.1; LdBPK_330990.1; 60S ribosomal protein L3 (LdBPK_323320.1), L15 (LdBPK_303710.1), L35a (LdBPK_342240.1); LdBPK_352220.1; ATP-dependent DEAD/H RNA helicase (LdBPK_353150.1); short chain dehydrogenase-like protein (LdBPK_363570.1); NIPSNAP (LdBPK_363620.1); LdBPK_050770.1; LdBPK_060440.1; LdBPK_080130.1; LdBPK_080800.1; LdBPK_100800.1; tubulin-tyrosine ligase-like protein(LdBPK_110400.1); 3-methylcrotonoyl-CoA carboxylase beta subunit (LdBPK_110600.1); kinesin motor domain containing protein (LdBPK_170270.1); LdBPK_171300.1; chaperone DNAJ protein (LdBPK_181410.1); 4-coumarate:coa ligase-like protein (LdBPK_190940.1); cullin-like protein-like protein (LdBPK_242380.1); LdBPK_252460.1; LdBPK_290470.1; protein kinase (LdBPK_301580.1); LdBPK_302440.1; 3-ketoacyl-CoA thiolase-like protein (LmjF.31.1630); LdBPK_321890.1; LdBPK_340270.1; LdBPK_341610.1; LdBPK_342300.1; LdBPK_352020.1; LdBPK_352100.1; LdBPK_352650.1 LdBPK_354190.1; asparaginase-like protein (LdBPK_364650.1); LdBPK_366150.1; LdBPK_366240.1; protein-l-isoaspartate o-methyltransferase (LdBPK_366860.1).
