# Supplementary material for: Characterization of the dual functions of Leishmania CK1.2 in both the parasite and the macrophage
Source: PLoS One. 2026 Oct 5;21(10):e0356818. doi: 10.1371/journal.pone.0356818 (PMC13638044; doi:10.1371/journal.pone.0356818)
Supplement: Supplementary file 10 [file pone.0356818.s012.pdf]

Table S7

GO	Name	Count1	Count2	Rate1	Rate2	OR	CI_lower	CI_upper	p_value	FDR_BH	Signif	Direction
GO:0005737	cytoplasm	68	0	0,81	0,00	959,00	56,63	16240,55	3,40E-38	2,96E-36	True	▲ pro-Tb-CC
GO:0043227	membrane-bounded organelle	0	95	0,00	0,83	0,00	0,00	0,02	3,19E-37	1,39E-35	True	▼ ama-Tb-CC
GO:0043231	intracellular membrane-bounded organelle	0	82	0,00	0,71	0,00	0,00	0,04	3,80E-29	1,10E-27	True	▼ ama-Tb-CC
GO:0031981	nuclear lumen	31	0	0,37	0,00	136,01	8,17	2264,79	4,97E-14	1,08E-12	True	▲ pro-Tb-CC
GO:0044464	obsolete cell part	0	44	0,00	0,38	0,01	0,00	0,16	4,53E-13	7,88E-12	True	▼ ama-Tb-CC
GO:0044428	obsolete nuclear part	25	0	0,30	0,00	99,00	5,92	1654,81	3,89E-11	5,65E-10	True	▲ pro-Tb-CC
GO:0012505	endomembrane system	0	33	0,00	0,29	0,01	0,00	0,24	1,68E-09	2,09E-08	True	▼ ama-Tb-CC
GO:0031967	organelle envelope	0	25	0,00	0,22	0,02	0,00	0,35	3,65E-07	3,52E-06	True	▼ ama-Tb-CC
GO:0031975	envelope	0	25	0,00	0,22	0,02	0,00	0,35	3,65E-07	3,52E-06	True	▼ ama-Tb-CC
GO:0005856	cytoskeleton	0	23	0,00	0,20	0,02	0,00	0,39	1,47E-06	1,28E-05	True	▼ ama-Tb-CC
GO:0140513	nuclear protein-containing complex	0	22	0,00	0,19	0,02	0,00	0,41	2,99E-06	2,36E-05	True	▼ ama-Tb-CC
GO:0005635	nuclear envelope	0	20	0,00	0,17	0,03	0,00	0,46	1,26E-05	9,12E-05	True	▼ ama-Tb-CC
GO:0015630	microtubule cytoskeleton	0	15	0,00	0,13	0,04	0,00	0,65	0,00020519	0,00137317	True	▼ ama-Tb-CC
GO:1990904	ribonucleoprotein complex	32	18	0,38	0,16	3,26	1,68	6,33	0,00045481	0,0026379	True	▲ pro-Tb-CC
GO:0005840	ribosome	32	18	0,38	0,16	3,26	1,68	6,33	0,00045481	0,0026379	True	▲ pro-Tb-CC
GO:0022626	cytosolic ribosome	27	13	0,32	0,11	3,63	1,76	7,51	0,0005436	0,0029558	True	▲ pro-Tb-CC
GO:0005815	microtubule organizing center	0	13	0,00	0,11	0,04	0,00	0,77	0,00075652	0,00365653	True	▼ ama-Tb-CC
GO:0036064	ciliary basal body	0	13	0,00	0,11	0,04	0,00	0,77	0,00075652	0,00365653	True	▼ ama-Tb-CC
GO:0044391	ribosomal subunit	29	16	0,35	0,14	3,21	1,61	6,37	0,00097614	0,00446972	True	▲ pro-Tb-CC
GO:0030863	cortical cytoskeleton	6	0	0,07	0,00	19,13	1,06	344,42	0,00508506	0,02212003	True	▲ pro-Tb-CC
GO:0031080	nuclear pore outer ring	0	10	0,00	0,09	0,06	0,00	1,03	0,00560927	0,02323842	True	▼ ama-Tb-CC
GO:0043232	intracellular non-membrane-bounded organelle	52	48	0,62	0,42	2,25	1,27	3,98	0,00632962	0,02394248	True	▲ pro-Tb-CC
GO:0043228	non-membrane-bounded organelle	52	48	0,62	0,42	2,25	1,27	3,98	0,00632962	0,02394248	True	▲ pro-Tb-CC
GO:0005643	nuclear pore	4	20	0,05	0,17	0,26	0,09	0,75	0,00757201	0,02744854	True	▼ ama-Tb-CC
GO:0005730	nucleolus	22	13	0,26	0,11	2,73	1,30	5,75	0,00818144	0,02847141	True	▲ pro-Tb-CC
GO:0020016	ciliary pocket	0	9	0,00	0,08	0,07	0,00	1,16	0,01105847	0,03700335	True	▼ ama-Tb-CC
GO:0000313	organellar ribosome	5	0	0,06	0,00	15,98	0,87	293,12	0,01248737	0,04023709	True	▲ pro-Tb-CC
GO:0022627	cytosolic small ribosomal subunit	11	4	0,13	0,03	3,88	1,25	11,99	0,01426876	0,04433508	True	▲ pro-Tb-CC
GO:0030054	cell junction	0	8	0,00	0,07	0,07	0,00	1,32	0,02185381	0,06556144	False	▼ ama-Tb-CC
GO:0005829	cytosol	29	23	0,35	0,20	2,09	1,11	3,95	0,02328945	0,0675394	False	▲ pro-Tb-CC
GO:0015935	small ribosomal subunit	13	6	0,15	0,05	3,18	1,19	8,49	0,02566761	0,0720349	False	▲ pro-Tb-CC
GO:0030684	preribosome	4	0	0,05	0,00	12,91	0,69	243,19	0,03043797	0,07788539	False	▲ pro-Tb-CC
GO:0032040	small-subunit processome	4	0	0,05	0,00	12,91	0,69	243,19	0,03043797	0,07788539	False	▲ pro-Tb-CC
GO:0022625	cytosolic large ribosomal subunit	16	9	0,19	0,08	2,70	1,15	6,34	0,02878687	0,07788539	False	▲ pro-Tb-CC
GO:0070161	anchoring junction	0	6	0,00	0,05	0,10	0,01	1,79	0,04028468	0,09735464	False	▼ ama-Tb-CC
GO:0120119	flagellum attachment zone	0	6	0,00	0,05	0,10	0,01	1,79	0,04028468	0,09735464	False	▼ ama-Tb-CC
GO:0015934	large ribosomal subunit	16	10	0,19	0,09	2,42	1,05	5,56	0,05359644	0,12602407	False	▲ pro-Tb-CC
GO:0000315	organellar large ribosomal subunit	3	0	0,04	0,00	9,92	0,51	194,68	0,07365237	0,14416227	False	▲ pro-Tb-CC
GO:0020039	pellicle	3	0	0,04	0,00	9,92	0,51	194,68	0,07365237	0,14416227	False	▲ pro-Tb-CC
GO:0031428	box C/D RNP complex	3	0	0,04	0,00	9,92	0,51	194,68	0,07365237	0,14416227	False	▲ pro-Tb-CC
GO:0016604	nuclear body	3	0	0,04	0,00	9,92	0,51	194,68	0,07365237	0,14416227	False	▲ pro-Tb-CC
GO:0051286	cell tip	0	5	0,00	0,04	0,12	0,01	2,18	0,07456669	0,14416227	False	▼ ama-Tb-CC
GO:0060187	cell pole	0	5	0,00	0,04	0,12	0,01	2,18	0,07456669	0,14416227	False	▼ ama-Tb-CC
GO:0005732	sno(s)RNA-containing ribonucleoprotein complex	3	0	0,04	0,00	9,92	0,51	194,68	0,07365237	0,14416227	False	▲ pro-Tb-CC
GO:0015030	Cajal body	3	0	0,04	0,00	9,92	0,51	194,68	0,07365237	0,14416227	False	▲ pro-Tb-CC
GO:0101031	chaperone complex	2	0	0,02	0,00	7,00	0,33	147,74	0,17694533	0,32753711	False	▲ pro-Tb-CC
GO:0005832	chaperonin-containing T-complex	2	0	0,02	0,00	7,00	0,33	147,74	0,17694533	0,32753711	False	▲ pro-Tb-CC
GO:0032991	protein-containing complex	44	50	0,52	0,43	1,43	0,81	2,50	0,25069193	0,44253611	False	▲ pro-Tb-CC
GO:0000145	exocyst	0	3	0,00	0,03	0,19	0,01	3,73	0,26450434	0,44253611	False	▼ ama-Tb-CC
GO:0035859	Seh1-associated complex	0	3	0,00	0,03	0,19	0,01	3,73	0,26450434	0,44253611	False	▼ ama-Tb-CC
GO:0099023	vesicle tethering complex	0	3	0,00	0,03	0,19	0,01	3,73	0,26450434	0,44253611	False	▼ ama-Tb-CC
GO:0120118	flagella connector	0	3	0,00	0,03	0,19	0,01	3,73	0,26450434	0,44253611	False	▼ ama-Tb-CC
GO:0043226	organelle	74	107	0,88	0,93	0,56	0,22	1,45	0,31706498	0,52046515	False	▼ ama-Tb-CC
GO:0046930	pore complex	1	0	0,01	0,00	4,15	0,17	103,14	0,42211055	0,59231642	False	▲ pro-Tb-CC
GO:0044611	nuclear pore inner ring	1	0	0,01	0,00	4,15	0,17	103,14	0,42211055	0,59231642	False	▲ pro-Tb-CC
GO:0097165	nuclear stress granule	1	0	0,01	0,00	4,15	0,17	103,14	0,42211055	0,59231642	False	▲ pro-Tb-CC
GO:0043186	P granule	1	0	0,01	0,00	4,15	0,17	103,14	0,42211055	0,59231642	False	▲ pro-Tb-CC
GO:0045495	pole plasm	1	0	0,01	0,00	4,15	0,17	103,14	0,42211055	0,59231642	False	▲ pro-Tb-CC
GO:0060293	germ plasm	1	0	0,01	0,00	4,15	0,17	103,14	0,42211055	0,59231642	False	▲ pro-Tb-CC
GO:0031965	nuclear membrane	1	0	0,01	0,00	4,15	0,17	103,14	0,42211055	0,59231642	False	▲ pro-Tb-CC
GO:0031306	intrinsic component of mitochondrial outer membrane	1	0	0,01	0,00	4,15	0,17	103,14	0,42211055	0,59231642	False	▲ pro-Tb-CC
GO:0031307	integral component of mitochondrial outer membrane	1	0	0,01	0,00	4,15	0,17	103,14	0,42211055	0,59231642	False	▲ pro-Tb-CC
GO:0120260	ciliary microtubule quartet	0	2	0,00	0,02	0,27	0,01	5,67	0,50966956	0,70382939	False	▼ ama-Tb-CC
GO:0043229	intracellular organelle	74	104	0,88	0,90	0,78	0,32	1,90	0,64453848	0,87616949	False	▼ ama-Tb-CC
GO:0044297	cell body	0	1	0,00	0,01	0,45	0,02	11,23	1	1	False	▼ ama-Tb-CC
GO:0034663	endoplasmic reticulum chaperone complex	2	2	0,02	0,02	1,38	0,23	8,12	1	1	False	▲ pro-Tb-CC
GO:0034468	glycosome lumen	0	1	0,00	0,01	0,45	0,02	11,23	1	1	False	▼ ama-Tb-CC
GO:0042579	microbody	5	7	0,06	0,06	1,00	0,32	3,12	1	1	False	▲ pro-Tb-CC
GO:0034730	SmD-containing SMN-Sm protein complex	0	1	0,00	0,01	0,45	0,02	11,23	1	1	False	▼ ama-Tb-CC
GO:0031907	microbody lumen	0	1	0,00	0,01	0,45	0,02	11,23	1	1	False	▼ ama-Tb-CC
GO:0005622	intracellular anatomical structure	79	108	0,94	0,94	1,00	0,32	3,12	1	1	False	▼ ama-Tb-CC
GO:0005575	cellular component	80	109	0,95	0,95	1,06	0,31	3,65	1	1	False	▲ pro-Tb-CC
GO:0005788	endoplasmic reticulum lumen	2	2	0,02	0,02	1,38	0,23	8,12	1	1	False	▲ pro-Tb-CC
GO:0005782	peroxisomal matrix	0	1	0,00	0,01	0,45	0,02	11,23	1	1	False	▼ ama-Tb-CC
GO:0005777	peroxisome	5	7	0,06	0,06	1,00	0,32	3,12	1	1	False	▲ pro-Tb-CC
GO:0005634	nucleus	41	57	0,49	0,50	0,97	0,55	1,70	1	1	False	▼ ama-Tb-CC
GO:0000262	mitochondrial chromosome	0	1	0,00	0,01	0,45	0,02	11,23	1	1	False	▼ ama-Tb-CC
GO:0005938	cell cortex	6	6	0,07	0,05	1,39	0,45	4,30	0,7644025	1	False	▲ pro-Tb-CC
GO:0020015	glycosome	5	7	0,06	0,06	1,00	0,32	3,12	1	1	False	▲ pro-Tb-CC
GO:0020038	subpellicular network	1	1	0,01	0,01	1,37	0,14	13,42	1	1	False	▲ pro-Tb-CC
GO:0110165	cellular anatomical entity	80	109	0,95	0,95	1,06	0,31	3,65	1	1	False	▲ pro-Tb-CC
GO:0071024	SL snRNP	0	1	0,00	0,01	0,45	0,02	11,23	1	1	False	▼ ama-Tb-CC
GO:0044614	nuclear pore cytoplasmic filaments	0	1	0,00	0,01	0,45	0,02	11,23	1	1	False	▼ ama-Tb-CC
GO:0044615	nuclear pore nuclear basket	0	1	0,00	0,01	0,45	0,02	11,23	1	1	False	▼ ama-Tb-CC
GO:0044612	nuclear pore linkers	1	2	0,01	0,02	0,82	0,11	6,30	1	1	False	▼ ama-Tb-CC
GO:1905202	methylcrotonoyl-CoA carboxylase complex	0	1	0,00	0,01	0,45	0,02	11,23	1	1	False	▼ ama-Tb-CC
GO:1990130	GATOR1 complex	0	1	0,00	0,01	0,45	0,02	11,23	1	1	False	▼ ama-Tb-CC
