# Supplementary material for: Characterization of the dual functions of Leishmania CK1.2 in both the parasite and the macrophage
Source: PLoS One. 2026 Oct 5;21(10):e0356818. doi: 10.1371/journal.pone.0356818 (PMC13638044; doi:10.1371/journal.pone.0356818)
Supplement: Supplementary file 11 [file pone.0356818.s014.pdf]

Table S9

Name	Host interactors	Host substrates
telomere maintenance	-1,9348139	0,766549293
neutrophil activation involved in immune response	-1,06409668	1,04653331
nucleobase-containing compound metabolic process	-4,474226085	2,756079244
DNA metabolic process	-1,249143425	0,880637172
DNA repair	-1,313311473	0,907747276
rRNA processing	-1,119826454	0,746922101
regulation of translation	-1,576092264	1,72002012
negative regulation of protein kinase activity	-1,111297253	0,77004822
cellular aromatic compound metabolic process	-4,114351076	2,316057852
phosphate-containing compound metabolic process	-2,055181727	0,720233478
transport	-2,546566792	2,914804143
mitochondrial transport	-3,813069226	0,880637172
intracellular protein transport	-4,09247952	0,953249326
endoplasmic reticulum to Golgi vesicle-mediated transport	-0,994071646	0,845218416
nucleocytoplasmic transport	-1,111297253	1,382788331
apoptotic process	-1,77107694	1,775153119
activation of cysteine-type endopeptidase activity involved in apoptotic process	-1,090340034	0,880637172
response to stress	-1,985511634	2,128446941
cellular response to DNA damage stimulus	-0,963644771	1,320115007
response to oxidative stress	-1,090340034	0,783816643
organelle organization	-4,273815265	4,322388343
mitochondrial membrane organization	-1,599904077	0,841311336
cytoskeleton organization	-0,994115403	2,361354044
establishment or maintenance of cell polarity	-1,652489666	1,472136545
blood coagulation	-0,934806272	1,063301099
hemostasis	-0,934806272	1,04653331
protein localization	-2,71412985	1,606607553
metabolic process	-6,744240705	0,798566578
cell death	-1,610640321	1,854624176
response to abiotic stimulus	-1,510520134	1,30260113
response to endogenous stimulus	-1,509047168	1,021916132
positive regulation of metabolic process	-0,994213904	3,922164823
regulation of catabolic process	-1,269748789	1,04653331
regulation of signal transduction	-1,002644444	2,404042111
cellular process	-2,779638612	3,22229122
response to organic substance	-2,564307603	1,176299253
endomembrane system organization	-1,037179746	1,339464866
gene expression	-1,634887842	2,460234063
positive regulation of macromolecule metabolic process	-1,145402168	4,268266644
posttranscriptional regulation of gene expression	-1,304845855	2,089529265
regulation of cell communication	-0,938164218	2,361354044
regulation of mitochondrion organization	-0,907794473	0,770410283
regulation of cell death	-2,0688915	2,626942959
programmed cell death	-1,69050651	1,274899138
protein transport	-2,449384109	1,04653331
viral process	-1,258703451	5,174327076
cellular component organization	-2,260835325	3,573665564
rRNA metabolic process	-1,100489721	0,710291089
vesicle-mediated transport	-1,090340034	2,86826153
Name	Host interactors	Host substrates
endosomal transport	-1,111297253	2,162421064
modulation by virus of host process	-0,905695662	0,704825459
viral life cycle	-1,00227596	0,813815415
regulation of metabolic process	-0,97622163	3,786481581
hindbrain structural organization	-1,402586718	0,880637172
cerebellum structural organization	-1,402586718	0,880637172
cell cycle process	-0,896645575	0,833311258
cellular component assembly	-1,525021384	2,484297491
ribonucleoprotein complex biogenesis	-2,560094867	2,361354044
ribonucleoprotein complex assembly	-2,5318647	1,163390576
regulation of signaling	-0,934806272	2,887292554
establishment of cell polarity	-1,402586718	1,385555058
vesicle transport along actin filament	-0,91788087	0,919166372
regulation of cell migration	-1,202429197	1,883783994
intracellular receptor signaling pathway	-0,957019705	0,781400308
regulation of cellular metabolic process	-0,980248186	4,984655148
negative regulation of cellular metabolic process	-0,905695662	4,253352463
positive regulation of cellular metabolic process	-1,06851332	4,856080619
regulation of cellular catabolic process	-1,185948628	1,63160078
positive regulation of protein-containing complex assembly	-1,145199768	2,271649178
regulation of protein modification process	-1,291139549	1,638649082
regulation of cellular protein metabolic process	-1,792786769	1,987680848
negative regulation of cellular protein metabolic process	-1,090340034	1,882643424
positive regulation of protein polymerization	-1,067459669	0,880637172
DNA geometric change	-1,074494567	1,04653331
DNA duplex unwinding	-1,090340034	1,065709962
regulation of localization	-0,965334264	3,915548764
regulation of protein localization	-1,58587896	1,882218376

Table S9

Name	Host interactors	Host substrates
regulation of mast cell apoptotic process	-1,187667775	0,786949352
macromolecule localization	-2,048234417	2,00479335
regulation of organelle organization	-1,040045175	2,007827438
cellular response to stress	-1,390566284	2,50712294
negative regulation of kinase activity	-1,06409668	0,91389533
response to cytokine	-2,575657702	1,78186466
regulation of cellular amide metabolic process	-1,398609668	1,682870152
negative regulation of cellular amide metabolic process	-1,851131076	0,817822825
cellular protein localization	-3,448806736	1,848625507
cellular protein-containing complex assembly	-1,543762886	1,070458097
cellular nitrogen compound metabolic process	-6,554879796	1,895184814
synaptic vesicle recycling	-1,249143425	0,81251283
regulation of locomotion	-1,090340034	1,964095758
protein refolding	-2,003322778	0,889847495
ribosome biogenesis	-1,304845855	1,103463968
regulation of apoptotic process	-2,416491123	2,486892296
negative regulation of apoptotic process	-1,851131076	0,950339632
regulation of programmed cell death	-2,335757336	2,404042111
negative regulation of programmed cell death	-1,808115799	0,907281992
positive regulation of catalytic activity	-1,402586718	3,436103786
macromolecule metabolic process	-2,473239647	1,310651976
regulation of protein-containing complex assembly	-1,03740929	3,399584577
regulation of cysteine-type endopeptidase activity involved in apoptotic process	-1,090340034	1,030177867
protein-containing complex subunit organization	-1,145199768	1,750223329
cellular component biogenesis	-1,894896774	3,177135894
regulation of cellular component biogenesis	-1,187667775	1,667920723
positive regulation of molecular function	-1,831700393	4,503070427
cellular metabolic process	-6,822810781	1,106918236
primary metabolic process	-4,273815265	0,975691528
cellular macromolecule metabolic process	-3,181055231	0,795982104
protein import into mitochondrial intermembrane space	-1,249143425	0,786949352
establishment of protein localization	-2,716304913	0,880637172
heterocycle metabolic process	-4,26466829	2,415779047
intracellular transport	-4,665050422	4,749654522
negative regulation of biological process	-1,255734121	6,139556677
positive regulation of cellular process	-0,934806272	7,135942844
negative regulation of cellular process	-1,145199768	6,538901134
regulation of response to stimulus	-1,108753624	3,497787339
negative regulation of response to stimulus	-1,304258208	0,925270744
regulation of catalytic activity	-2,560094867	3,645302617
coagulation	-0,934806272	1,055705331
response to stimulus	-1,111297253	1,198121655
regulation of cellular component organization	-1,307199547	3,97043308
nuclear transport	-1,111297253	2,185393897
regulation of nitrogen compound metabolic process	-1,141913795	4,611193938
negative regulation of nitrogen compound metabolic process	-1,013174694	3,695421359
positive regulation of nitrogen compound metabolic process	-1,18945064	3,813428199
localization	-1,760741166	3,453573839
regulation of protein transport	-1,155319808	0,816757566
establishment of localization	-2,716304913	2,955433641
regulation of protein metabolic process	-2,258400634	1,57919871
negative regulation of protein metabolic process	-1,040451593	1,664139599
regulation of cellular component movement	-1,056924227	2,08273099
chromosome organization	-1,012850036	1,062818266
regulation of hydrolase activity	-2,277079615	3,719116748
positive regulation of hydrolase activity	-2,20899525	3,786481581
negative regulation of transferase activity	-0,987765556	1,029390683
cellular localization	-3,806217685	7,135257255
establishment of localization in cell	-4,265200274	7,135942844
regulation of macromolecule metabolic process	-1,037179746	4,613307221
regulation of cellular localization	-1,304845855	2,469518836
negative regulation of cell death	-2,000893044	1,183775495

Table S9

Name	Host interactors	Host substrates
membrane organization	-1,145199768	2,162421064
cellular response to chemical stress	-0,97622163	0,724901946
protein-containing complex assembly	-1,297080215	1,466784931
regulation of biological quality	-0,97622163	1,283605577
regulation of molecular function	-2,211166814	4,987492124
regulation of establishment of protein localization	-1,111297253	0,76517019
cellular macromolecule localization	-3,442064252	1,809729048
response to growth factor	-1,233567182	0,726028972
cellular response to chemical stimulus	-3,32003978	1,237306464
cellular response to abiotic stimulus	-0,905695662	3,637213233
cellular response to organic substance	-3,107748277	1,666977539
cellular response to cytokine stimulus	-2,999492267	1,896765808
cellular response to interleukin-4	-2,786058065	0,724901946
cellular response to growth factor stimulus	-1,277430942	0,798566578
cellular response to radiation	-1,249143425	2,241247753
cellular response to endogenous stimulus	-1,040451593	0,862578664
organic substance transport	-2,267499657	1,190916603
organic substance metabolic process	-4,273815265	0,724901946
nitrogen compound transport	-2,522660164	1,572660621
ribonucleoprotein complex subunit organization	-2,489050217	1,513370881
cellular component organization or biogenesis	-2,716304913	4,146825515
DNA biosynthetic process	-1,145199768	0,832320095
regulation of primary metabolic process	-1,225178693	4,702460646
regulation of response to stress	-1,402586718	1,135961236
regulation of cellular response to stress	-1,323504848	1,190916603
nucleic acid metabolic process	-1,249143425	4,199722647
cellular response to environmental stimulus	-0,905695662	3,637213233
regulation of cell-substrate junction organization	-1,187667775	0,722618488
organic cyclic compound metabolic process	-3,668611592	2,670234803
regulation of intracellular signal transduction	-0,896271073	2,396415003
positive regulation of protein localization	-1,288726193	1,04653331
response to methamphetamine hydrochloride	-1,402586718	0,880637172
regulation of protein localization to membrane	-1,144096393	1,944052281
positive regulation of protein localization to membrane	-1,548972844	1,822250214
response to leukemia inhibitory factor	-0,938963801	0,820928264
cellular response to leukemia inhibitory factor	-0,939264287	0,832320095
regulation of cysteine-type endopeptidase activity	-1,030577344	0,884006529
regulation of cell motility	-1,144096393	2,165901616
