# Supplementary material for: Characterization of the dual functions of Leishmania CK1.2 in both the parasite and the macrophage
Source: PLoS One. 2026 Oct 5;21(10):e0356818. doi: 10.1371/journal.pone.0356818 (PMC13638044; doi:10.1371/journal.pone.0356818)
Supplement: Supplementary file 12 [file pone.0356818.s015.pdf]

Table S10

**Name**	**Host**	***Leishmania***
nucleoside triphosphate metabolic process	-1,634887842	0,845331977
nucleoside triphosphate biosynthetic process	-2,003322778	0,911892141
purine nucleoside triphosphate metabolic process	-1,922303214	0,937490883
purine nucleoside triphosphate biosynthetic process	-2,260835325	0,937490883
ribonucleoside triphosphate metabolic process	-1,922303214	0,911892141
ribonucleoside triphosphate biosynthetic process	-2,171304355	0,911892141
purine ribonucleoside triphosphate metabolic process	-3,666283962	0,845331977
purine ribonucleoside triphosphate biosynthetic process	-2,277079615	0,937490883
nitrogen compound metabolic process	-4,582056065	1,81450507
cellular nitrogen compound metabolic process	-6,554879796	3,073679025
organic substance metabolic process	-4,273815265	1,651268279
primary metabolic process	-4,273815265	1,817208507
cellular metabolic process	-6,822810781	1,127283258
metabolic process	-6,744240705	0,967200494
amide biosynthetic process	-3,666283962	11,25767592
organonitrogen compound metabolic process	-5,356074596	3,799531597
cellular amide metabolic process	-4,399749773	10,81014482
macromolecule metabolic process	-2,473239647	2,090232071
biosynthetic process	-5,019714576	4,286573329
macromolecule biosynthetic process	-4,582056065	6,659381657
cellular macromolecule biosynthetic process	-4,605568491	6,659381657
peptide biosynthetic process	-4,273815265	11,47606679
cellular biosynthetic process	-4,869140561	4,790977609
organonitrogen compound biosynthetic process	-4,903912762	7,949668097
organic substance biosynthetic process	-5,127489825	4,567306043
cellular nitrogen compound biosynthetic process	-5,019714576	7,560669609
peptide metabolic process	-4,273815265	11,25767592
protein metabolic process	-3,05683474	4,567306043
cellular protein metabolic process	-3,285108093	5,267728267
cellular macromolecule metabolic process	-3,181055231	2,701439386
fructose 6-phosphate metabolic process	-0,957019705	0,911892141
nuclear transport	-1,111297253	2,148849947
nucleocytoplasmic transport	-1,111297253	2,148849947
vesicle-mediated transport	-1,090340034	1,54578369
protein transport	-2,449384109	2,359471648
intracellular protein transport	-4,09247952	0,911892141
intracellular transport	-4,665050422	1,863173798
peptide transport	-1,225178693	2,831006668
nitrogen compound transport	-2,522660164	2,241079364
organic substance transport	-2,267499657	1,841364076
transport	-2,546566792	1,651268279
establishment of localization	-2,716304913	1,644213334
establishment of localization in cell	-4,265200274	1,863173798
localization	-1,760741166	1,22366345
macromolecule localization	-2,048234417	2,332886628
cellular macromolecule localization	-3,442064252	0,839235467
protein localization	-2,71412985	2,241079364
cellular protein localization	-3,448806736	0,839235467
establishment of protein localization	-2,716304913	2,332886628
cellular localization	-3,806217685	1,651268279
translation	-4,357048852	11,47606679
gene expression	-1,634887842	6,659381657
protein folding	-2,779638612	3,168216442
protein refolding	-2,003322778	4,917207152
cellular process	-2,779638612	3,336585847
